# A Well-Kept Treasure at Depth: Precious Red Coral Rediscovered in Atlantic Deep Coral Gardens (SW Portugal) after 300 Years

**DOI:** 10.1371/journal.pone.0147228

**Published:** 2016-01-22

**Authors:** Joana Boavida, Diogo Paulo, Didier Aurelle, Sophie Arnaud-Haond, Christian Marschal, John Reed, Jorge M. S. Gonçalves, Ester A. Serrão

**Affiliations:** 1 CCMAR, Centro de Ciências do Mar, Universidade do Algarve, Campus of Gambelas, 8005-139, Faro, Portugal; 2 Aix Marseille Université, CNRS, IRD, Avignon Université, IMBE UMR 7263, 13007, Marseille, France; 3 Ifremer, UMR MARBEC (Marine Biodiversity, Exploitation and Conservation) Bd Jean Monnet, BP 171, F-34203, Sète, France; 4 Harbor Branch Oceanographic Institute, Florida Atlantic University, Fort Pierce, United States of America; University of Genova, ITALY

## Abstract

**Background:**

The highly valuable red coral *Corallium rubrum* is listed in several Mediterranean Conventions for species protection and management since the 1980s. Yet, the lack of data about its Atlantic distribution has hindered its protection there. This culminated in the recent discovery of poaching activities harvesting tens of kg of coral per day from deep rocky reefs off SW Portugal. Red coral was irregularly exploited in Portugal between the 1200s and 1700s, until the fishery collapsed. Its occurrence has not been reported for the last 300 years.

**Results:**

Here we provide the first description of an Atlantic red coral assemblage, recently rediscovered dwelling at 60–100 m depth in southern Portugal. We report a very slow growth rate (0.23 mm year^-1^), comparable to Mediterranean specimens. In comparison with most of the Mediterranean reports, the population reaches much larger sizes, estimated to be over one century old, and has a more complex coral branch architecture that promotes a rich assemblage of associated species, with boreal and Mediterranean affinities. Atlantic red coral is genetically distinct, yet mitochondrial analyses suggest that red corals from the Atlantic may have introgressed the Mediterranean ones after migration via the Algeria current. Our underwater surveys, using advanced mixed-gas diving, retrieved lost fishing gear in all coral sites. Besides illegal harvesting, the use and loss of fishing gears, particularly nets, by local fisheries are likely sources of direct impacts on these benthic assemblages.

**Conclusions:**

We extended the knowledge on the distribution of *C*. *rubrum* in the Atlantic, discovered its genetic distinctiveness, and reveal a rich deep-dwelling fauna associated to these coral assemblages. These findings support a barrier role of the Atlantic-Mediterranean transition zone, but reveal also hints of connectivity along its southern margin. The results highlight the genetic and demographic uniqueness of red coral populations from SW Iberia. However, we also report threats to these vulnerable populations by direct and indirect fishing activities and argue that its protection from any mechanically destructive activities is urgent as a precautionary approach. This study advances our understanding of phylogeographic barriers and range edge genetic diversity, and serves as a baseline against which to monitor future human and environmental disturbances to Atlantic *C*. *rubrum*.

## Introduction

The precious red coral, *Corallium rubrum* (Linnaeus, 1758), valued for its use in jewelry, has been highly exploited in the Mediterranean since Antiquity [1, 2]. It is listed under several conventions (SPAMI, Annex III; Bern Convention, Annex III; Habitats Directive, Annex V; Barcelona Convention) and under multi-government legislation that regulates its exploitation (e.g., fishing licenses; ban of fishing shallower than 50 m in some areas [3]), apart from national protection by some countries (e.g., MPAs in Spain, France, Italy, Croatia, Albania). It plays an important ecological role by providing structural habitat for other species (“habitat engineer” *sensu* [4]). This coral has been widely studied in the western Mediterranean, including in deeper mesophotic areas [5, 6]. Despite extensive progress in red coral research, including reproductive ecology (e.g., [7]), demography (e.g., [8]) and unmistaken data showing its overexploitation across the Mediterranean, management and enforcement have consistently failed thus far [3, 9].

**Fig 1:**
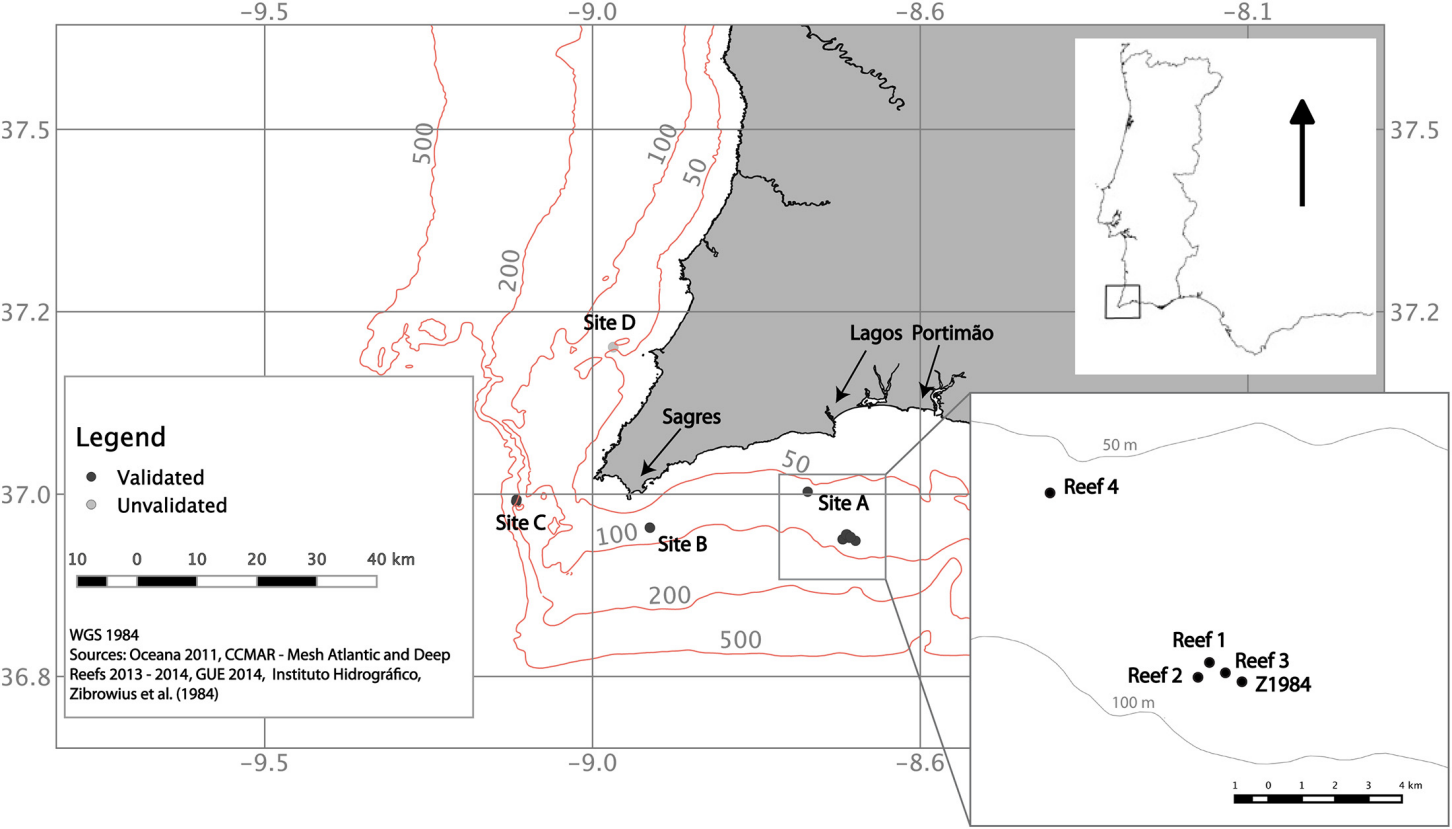
Southwestern Portugal red coral gardens (Atlantic). Known locations of *Corallium rubrum* along the Portuguese coastline. Black symbols—red coral occurrences validated in this study (Sites A-C). Grey symbol in Site D—unverified red coral location. Bathymetry—50, 100, 200 and 500 m. Site A—location of the 2014 survey dive sites (Reefs 1–4) and sampling station of [10] (code Z1984; dead fragments collected in 1979). Reefs 1–3—Portuguese Maritime Police coral capture area (see section 2).

Lack of data about its Atlantic distribution has rendered red coral an elusive species in this region. Historical references, notably summarized in [10] (in French), have indicated that coral fisheries led by Portugal existed in the Atlantic but appear to have been abandoned about 300 years ago (see Box 1). The previous occurrence of directed fisheries followed by hundreds of years without records of the species occurrence in the region suggests a "boom and bust" harvesting strategy that may have resulted in significant declines in catch and collapse of the stock, which would represent one of the oldest cases reported. This may have been the cause for the present generalized void of knowledge about the presence of this species in SW Iberia, despite having been an important fisheries resource in the distant past. This is a typical example of shifting baselines [11], reducing our expectations towards coastal ecosystems (e.g., [12]). Only small dead fragments were collected off the Atlantic coast of Iberia, once, in over 30 years [10]. The fact that the species was only reported from fisheries by-catch [13] and its absence from shallow areas, including submerged caves [14] where it is typically found in the Mediterranean, suggested that it was not widely distributed in this coast or that it was limited to deeper, more inaccessible areas. Given the geographic proximity, this difference from its Mediterranean counterparts is not readily explained but could be related to a range edge shift in depth distribution in SW Iberia. Red coral is also currently exploited along Atlantic coasts of Morocco [15], but the benthic ecology of *C*. *rubrum* in the Atlantic remains undocumented. Here we provide the first in-depth assessment of a recently rediscovered deep red coral habitat (60–100 m) in the Atlantic Ocean, filling significant knowledge gaps. We address the following questions and subjects: (1) Are these Atlantic red coral populations genetically differentiated from Mediterranean populations? (2) Do SW Iberian populations contain older and more complex individuals, as expected in pristine, undisturbed populations? (3) What are the main threats these deep red coral gardens face? We describe the assemblage of associated species of the deep red coral reefs in SW Iberia. Additionally, we review historical literature concerning ancient red coral fisheries and its collapse centuries ago, and discuss related observations gathered from multiple sources (a large red coral catch apprehended from poachers, deep manned underwater exploration and fisheries by-catch). We provide a map with new red coral locations in Portugal (Fig 1) and compare our findings with data reported in the literature for the Mediterranean. Information about red coral off SW Iberia are urgently needed and will be a key instrument for future research and conservation plans.

**Box1. *Historical overview and modern time occurrences of red coral in Portugal*.**

It is uncertain when corals started to be collected along the Portuguese coastline. Objective historical references of this fishery in Portugal go back to the mid 15th century where some information is available regarding the taxes applied on this fishery and its decline [16–20]. Others indicate that the coral fishery had begun in Lagos between the 13th and early 14th centuries after Italians established there [21] and that it remained important for the Portuguese trade with India in the 15th century [22]. A review about precious corals by [23] indicates that Lisbon (Portugal) was the main center for precious red coral jewelry manufacture between the 14th and 17th centuries, likely owing to the central position it took in maritime trading routes during this period. From this time to the early 18th century there are no records about any coral fishery or trade in Portugal and it is known to have declined dramatically during this period [17]. There was an attempt to reintroduce the coral trade in Algarve in 1711 [16, 17, 22]. However, this attempt must have been somewhat unsuccessful since in 1790 the Queen D. Maria I ordered a research about the causes of the decline in the coral fishery of Algarve [17]. By this time, fishermen had lost memory of that fishery, reporting that for over 70 years there had been no coral fishery in Algarve and that no one had ever seen any coral again since the introduction of the use of gears called “*covãos*” [22], local fishing gear similar to modern cylindrical traps used to catch demersal fish. The putative relation between the use of *covãos* and the decline of the coral fishery remains unclear. There is no further information about the fate of this fishery since then, and the amounts harvested are unknown, but the fishery appears to have been completely abandoned. The absence of red coral references in the Portuguese zoological literature of the time [10] indicates that the species would have been rare if at all present in locations accessible to researchers. Our investigation indicates that currently red coral occurs along the deep (mostly c.a. 100 m) southwestern coast of Portugal but may extend a little northwards to rocky banks located off an MPA north of Sagres (Site D in Fig 1).

Other Atlantic locations where Portuguese traders exploited red coral were from Cape Spartel to Azilah in North Morocco [16] and in Cape Verde, likely at Santiago island [10, 24], which were both Portuguese colonies at the time (15^th^ and 19^th^ centuries, respectively).

Clear reasons for abandoning the red coral fishery in the south of Portugal are unknown but are likely related with two factors: 1) the slow growth (<0.5 mm year^-1^) combined with limited, patchy, coral habitat and harvest might have led to a dramatic decrease in the number of corals which did not have time to recover to sustain the fishery, and 2) the inability to compete with Mediterranean traders, where large amounts of coral were more easily collected at shallower depths (S3 Fig). This case might thus represent one of the oldest historically documented collapses of directed fisheries, as Atlantic red coral off SW Iberia underwent fishery-related impacts for over five centuries culminating in the total absence of records of the species in the harvested area for the last three centuries. In fact, the historical references state precisely that “in the coasts of the kingdom of Algarve there was a coral fishery, which was lost by carelessness (*incúria*) of men, or by lack of resources (*cabedais*)” [22]. Given the substantial technological advances now existing relative to historical coral fisheries efforts, modern-time extractive activities directed at this species in SW Iberia could have dramatic implications resulting in a population collapse again, but much faster than it did centuries ago. Understanding historical perspectives remains valuable for managers to avoid repeating the mistakes of the past and to try to conciliate sustainable resource practices without being biased by the lack of knowledge of the real baseline [11, 25–28].

## Material and Methods

### Ethics Statement

All field surveys in Portugal were performed with authorisations issued by the Portuguese Navy and Nature Conservation Institute.

Data about the Atlantic red coral were obtained from several sources (see sections 2.1 and 2.2).

### 2.1. Study areas, sampling and associated fauna

Data on the occurrence of the red coral reefs and associated macrofauna of southwest Portugal were collected during three cruises. Data from the first two cruises were included here only for an updated distribution of the red coral along the southern coast of Portugal, whereas the third cruise focused on a detailed sampling, which we describe next. 1) In 2011 the OCEANA Ranger Expedition (eu.oceana.org) in collaboration with CCMAR used a remotely operated vehicle (ROV) off Sagres and documented the occurrence of the species at this location (Site C in Fig 1; see [29] for details). 2) In August 2013, ten exploratory ROV dives and sampling were performed off Lagos-Portimão, Reefs 1–3 at Site A (Fig 1), using a ROV from CCMAR. This ROV is equipped with a high-definition camera, lights and a grab, as described in [30]. The objective of these dives was to do a preliminary characterization of *C*. *rubrum* populations at the coordinates provided by the Portuguese Maritime Police (MP; see section 2.2). Four red coral samples were collected for genetic analysis (see section 2.5.). 3) In July 2014, during the Global Underwater Expedition 2014 (www.globalsubdive.com/expeditions), rebreather divers conducted five exploratory dives at four sites off Lagos-Portimão using advanced mixed-gas mixtures on board the vessel *Pacific Provider* (Site A, Reefs 1–4 in Fig 1). Twelve red coral samples were collected for genetic analysis (see section 2.5.). Benthic diver-operated video (DOV) transects about 100 m long (recorded with a transect line) at 60–100 m depth were dedicated to habitat characterization and species identification. Given the exploratory nature of the deep surveys and the decompression time constraints at these depths, the sampling strategy followed a detailed inspection at the reef scale: transects were made along the top of the reef with cameras positioned oblique towards the substrate to enable faunal observations, followed by a second, more detailed, transect to facilitate observation of smaller and incrusting taxa. In steeply sloping areas the camera was kept facing the vertical reef walls and on flat areas the camera was kept at a maximum of 50 cm facing the bottom. This complex video-transect design allowed a complete documentation of the assemblage at the rocky reefs. An additional 3 hours dive was made in the Triton submersible *Nemo* to complete benthic observations (for technical specifications see http://globalsubdive.com/resources/manned-submersibles).

Two video systems were used fixed to diver propulsion vehicles (DPV; Halcyon HDVexp): A Canon EOS 5D Mk3 or a GoPro Hero 3+ camera with either 1080 or 1536 lines of resolution and external LED lights 2x4000 lumen (Light & Motion Sola Video 4000). DPVs were used at approximately 10 m / s to enable identification of megafauna by video imagery.

One trained person performed all video analysis; taxa identifications were validated by senior taxonomists (see acknowledgments). The identification of benthic fauna was done to the lowest possible level for all organisms larger than about 5 cm (measured using the transect line). Given the uncertainties in taxa identification uniquely by means of video, we adopted conservative best practices based on knowledge from the nearby benthic communities [31]. However, it is not possible to identify many deepwater organisms solely from imagery (also noted by Reed et al. [32]). These were assigned to classification criteria such as “Demospongiae 1” or, for example, “cf. *Poecillastra compressa*” when the morphology was consistent with a species but a sample would be required for confidence in the identification (cf. = uncertain identity in Latin; S1 Table). An image-based catalog of taxa was compiled and is presented in S1 File.

Data from the video-transects and submersible dive were used to describe the habitat and to characterize the Atlantic red coral communities by compiling a species list. Species were categorized following an adaptation from [33] into characteristic species—species living strictly on red coral; accompanying species—species that live in the three dimensional net formed by the coral branches and on the rocky reefs; and co-occurring species—those occurring in the free space around the red coral reefs (S1 Table).

At each reef three stations separated by approximately 50 m were chosen along the transect line to make *in situ* measurements of basal diameter for the five most representative red coral colonies with a caliper. Additionally, at Reef 4, measurements of height and width of six representative colonies of the abundant species *Savalia savaglia* and *Paramuricea clavata* were made in one sampling station. The low number of measurements at each sampling station was due to bottom and decompression time constraints.

For each dive conductivity and temperature profile data were obtained with a Schlumberger CTD which was attached to the divers’ equipment (Schlumberger Water Services Technology; S1 Fig).

To identify if there is impact of fishing activities on the red coral habitat, lost fishing gears observed during video-transects were annotated and categorized according to types (Lines, Nets and Cables). A description of the main threats identified is provided.

### 2.2. Acquisition of dry samples

In April 2012 the Center of Marine Sciences at the University of Algarve (CCMAR), Portugal, was contacted by the Portuguese MP to identify an illegal coral catch apprehended off the Lagos-Portimão area, collected at 80–100 m depth by scuba divers (Fig 1 and S2 Fig). This catch consisted of 32 kg of live red coral (*C*. *rubrum*, confirmed by COI sequencing; see genetic results) with the substrates still attached to the basal holdfasts in most cases (349 colonies in total, of which 184 with holdfast, see section 2.3).

### 2.3. Population size structure

Since the coral catch described in section 2.2 presented many broken colonies, only those showing a basal holdfast were used for further analysis. From a total of 184 colonies the basal diameter size structure was determined using a caliper (precision ± 0.05 mm), as the average maximum and minimum diameter at the inflexion point (c.a. 5 mm above the basal holdfast). Juveniles, here defined as the size class <1 mm, were very abundant but not quantified due to handling difficulties owing to the colonies being dry (breakage). We indicate the percentage of colonies with basal diameter above the minimum legal harvestable basal diameter (7 mm [34]) following [35]. In order to provide an indication of three-dimensional complexity, the total number of branches of each coral colony was determined. This method was chosen instead of the classification system based on tributary/source (T/S) ratio and branching order described by [36] and used by [35, 37] in red corals, since colonies in this coral catch were severely broken and would give inaccurate estimations of these parameters. Axis protuberances of less than 3 mm in length were not considered as branches. Skewness of the distribution of branch numbers was tested in R version 3.1.1 (Agostino test, R Foundation for Statistical computing, 2014).

### 2.4. Colony age and growth rate

The growth rate was estimated with the organic matrix staining method [38]. This method was applied to a subsample of 25 colonies spanning the entire basal diameter range in our sample (the remaining colonies from the catch were not available for this study). From this subset, several (13) colonies showed excess of holes made by perforating polychaetes and boring sponges, irregular morphologies, fissures and bivalve shells inside the axis that hindered the growth rings and could not be used for age estimation (e.g., [7, 8]). From the initial subset, 12 colonies were retained for the final age and growth rate estimations. Sections were cut from the basal holdfasts of the colonies, decalcified, stained and photographed under a stereomicroscope. Images were processed in Adobe Photoshop CS5 Extended version 12.0 to improve image quality and contrast. After defining the medullar region, age was estimated as the average number of rings counted along a minimum of three transects drawn in different directions of the section. Because the first four years do not produce discernible growth rings [38, 39], the final age of each colony was estimated by adding four to the number of rings counted. The annual growth rate was calculated by dividing the basal diameter by colony age. This relationship was then applied to all red coral colonies. The correlation between age and the annual growth rate was determined with Pearson correlation in R version 3.1.1.

### 2.5. Preliminary analysis of genetic differentiation between Atlantic and Mediterranean

A portion of the mitochondrial DNA, the putative mitochondrial control region (mtC), from each of 16 Atlantic red coral samples collected in 2013 and 2014 was used for comparison with Mediterranean red coral sequences (Table 1). Total genomic DNA was extracted from individual colonies (two to four polyps per colony or with scrapped coenenchyme) using two methods: CTAB protocol [40] with purification by standard chloroform:isoamyl alcohol (24:1) followed by DNA precipitation, or Sambrock et al. [41] protocol. PCR conditions for amplification of the putative mitochondrial control region followed [6], using the primers ND618510CkonojF (59-CCATAAAACTAGCTCCAACTATTCC-39) and COI16CkonojR (59-GGTTAGTAGAAAATAGCCAACGTG-39) with minor modifications (using GoTaq polymerase). Sequencing was done in an ABIPRISM 3130 sequencer at CCMAR. Mediterranean sequences were obtained at IMBE and were sent at Eurofins^®^ company for purification and sequencing. Chromatograms were viewed in FintchTV v. 1.4.0 (Geospiza Inc., USA). Sequence alignment was done with ClustalW on Geneious v. 8.1.4 [42] and alignments (321 bp sequences) were revised manually. The relationships among haplotypes were inferred with the median-joining algorithm implemented in Network 4.6 (available online at http://www.fluxus-engineering.com; [43]).

**Table 1:** Location of samples, depth, haplotype group (see Results) and GenBank accession numbers of *Corallium rubrum* sequences.

Basin	Location	Haplotype group	Depth (m)	N	Reference; GenBank Accession number
Atlantic—Algarve	Portugal—Lagos-Portimão	S	90	16	This study; KU314517–KU314649
Mediterranean—Alboran	Spain—Ceuta	S and M-1	42	13	This study; KU314517–KU314649
Mediterranean—SW Mediterranean	Algeria—Tenes	S	33–47	19	This study; KU314517–KU314649
Mediterranean—Gulf of Lion	France—Elvin (Côte Bleue)	M-1	12	14	This study; KU314517–KU314649
Mediterranean—Gulf of Lion	France—Elvin (Côte Bleue)	M-1	8	4	This study; KU314517–KU314649
Mediterranean—Gulf of Lion	France—Méjean (Côte Bleue)	M-1 and M-2	40	10	This study; KU314517–KU314649
Mediterranean—Gulf of Lion	France—Figuier (Marseille)	M-1 and M-2	8	14	This study; KU314517–KU314649
Mediterranean—Ligurian	France—Galeria (Corsica)	M-2	40	4	This study; KU314517–KU314649
Mediterranean—Ligurian	France—Porto (Corsica)	M-2 and M-3	20	12	This study; KU314517–KU314649
Mediterranean—Catalonia	France—Banyuls	M-2	20	4	This study; KU314517–KU314649
Mediterranean—Ligurian	France—Corsica	M-2	Deep	4	This study; KU314517–KU314649
Mediterranean—Ligurian	France—Corsica	M-2	79	1	This study; KU314517–KU314649
Mediterranean—Ligurian	France—Corsica	M-2	171	1	This study KU314517–KU314649
Mediterranean—Ligurian	Italy—Punta dell'Atare (Portofino)	M-1	35	14	This study; KU314517–KU314649
Mediterranean—Thyrrenian	Italy—Punta Sant'Angelo (Ischia island)	A-1	100–118	1	[6]; KC597702
Mediterranean—Thyrrenian	Italy—Rete Bruno (Elba island)	M-1	84–88	1	[6]; KC597700
Mediterranean—Thyrrenian	Italy—Rete Bruno (Elba island)	I	83–86	1	[6]; KC597701
Mediterranean—Adriatic	Croatia—Garmenjak Veli Islet	A-1	57	4	This study; KU314517–KU314649
Mediterranean—Adriatic	Albania—Sazan	A-1 and A-2	58	4	This study; KU314517–KU314649

### 2.6. Ancient red coral fisheries

We retrieved historical references about coral fisheries related to Portuguese fishermen and traders. Since our historical data collection did not allow any quantification of catch or effort (quantitative data were not officially reported until the 20th century), we present the red coral fishery descriptions in Box 1. Only data referring to territories that correspond to the contemporary political borders of Portugal were included.

## Results

### 3.1. Habitat description and community

**Fig 2:**
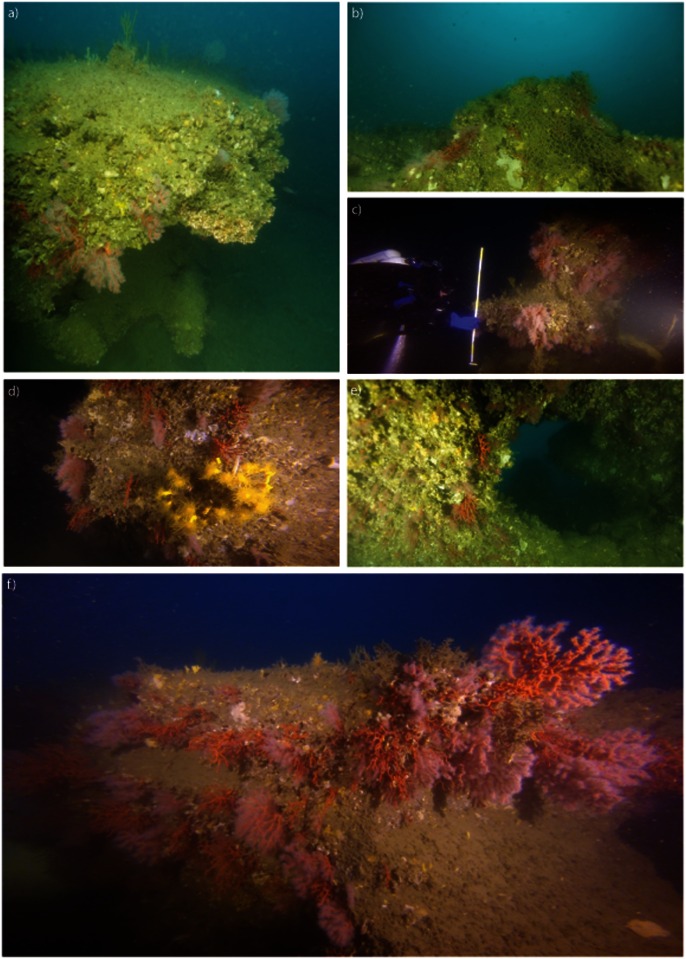
Aspects of the benthic assemblage of the red coral (*Corallium rubrum*) deep reefs off southern Portugal (Atlantic). Images were taken at Reefs 1–4 in Fig 1, from frames of the underwater video-transects by rebreather divers. **a)** Sparse red coral colonies over high relief rock with overhangs. Notice top of reef with low density of erect organisms (e.g., corals and sponges); **b)** Lost fishing net entangled on top of rock and corals; **c)** Diver measuring relief of substrate with a 1 m ruler (each yellow-white segment has 20 cm); **d)** Denser red coral patch and *Dendrophyllia cornigera* on vertical wall; **e)** Juveniles and small colonies in sheltered overhang; **f)** Higher density area over reef edges, note the larger colonies in this image; there is some distortion caused by the wide-angle and motion of the camera. Images kindly provided by the GUE Global Expedition 2014.

The region from the west to the south coast of Sagres (Sites B-D) presents hard substrate with steep slopes and a complex bottom including canyons, vertical walls and overhangs, as the land cliffs and capes extend into the ocean. Hydrodynamic forces are strong and depth at the surveyed sites reaches 130 m (e.g., [44]). The Lagos-Portimão area surveyed (Site A) has gentle slopes with rocky reefs of various sizes alternating with patches of muddy and detrital sand. Depth varies between 60 and nearly 100 m. At some sites, reefs present high roughness. Rocky outcrops can reach more than 2 m in height and width, rocks present multiple overhangs, caverns, fissures, arch and shelve structures which provide habitat for passive filter feeders and for sciaphillous species (Fig 2). Pronounced ripple marks were observed in the soft substrate produced by the prevailing W—E coastal longshore drift along the southern Portuguese coastline. The CTD temperature and conductivity profiles for each dive site showed water stratification with influence of the nearby Arade estuary at the surface and homogenization of the water column from about 10–20 m depth to the bottom (S1 Fig). At the seabed, where *C*. *rubrum* was found, the average temperature during the dives (July 2014; see section 2.1) was 14.0 ± 0.18°C and maximum salinity 36.4 PSU.

Red coral colonies were observed growing on hard substrates, predominantly rocky outcrops covered with dense patches of brachiopods (undetermined species) and deep-oysters (*Neopycnodonte cochlear*) that form deep-oyster banks over the rocks. Living colonies were red without epibionts and were mainly found on the crests and edges of rocks. Some colonies presented dead areas at the base, without coenenchyme and with epibiotic species. A total of 68 macro-benthic invertebrate and fish taxa were identified from the video surveys and 40 were tentatively identified to the species level (S1 Table) but 41 remain unidentified (data not shown). The complete taxa list is presented in S1 Table and an image-based catalog of taxa is provided in S1 File.

The most common species of the red coral community visible by video were, besides *C*. *rubrum*, a white cup bryozoan tentatively identified as *Reteporella* cf. *grimaldii* and the yellow cold-water coral *Dendrophyllia cornigera* (Fig 2). In the sample apprehended by the MP, the most common associated species, attached to the bases of nearly all colonies, was the oyster *Neopycnodonte cochlear* (less visible in the videos due to epibionts and their basal position), which carried also many red coral recruits < 1mm. In contrast to the lower abundance of fauna in the soft-bottom areas between rocky outcrops, the hard grounds were densely colonized by a diverse benthic assemblage that includes structuring species such as gorgonians, scleractinian corals; and cup, fan and massive demosponges. Fauna inhabiting the red coral community were similar to those living at the nearby shallower circalittoral reefs, e.g., *Paramuricea clavata*, *Eunicella* spp., *Savalia savaglia*, several sponges and echinoderms. Several *Astrospartus mediterraneus* were found attached to gorgonians or on crests of the rock outcrops. This basket starfish takes advantage of the height of the gorgonians to enhance the chance of getting food from currents. Flat top rocks were covered with sponges; notably white and yellow cup sponges such as *Phakellia ventilabrum*, fan sponges *P*. *robusta*, and a variety of incrusting and massive Demospongiae. The deep-sea lollipop Demospongiae *Stylocordyla* cf. *pellita* was observed for the first time in this coastal area and shallower than previously described (>400 m [45]). The ichtyofauna, composed by eight taxa (S1 Table) was dominated by the zooplanktivorous *Anthias anthias*. Others included large schools of unidentified clupeidae and the comber fish *Serranus cabrilla*. Few species of commercial relevance were observed: Lobsters (*Palinurus elephas*) were common in rock holes and gadidae pouting (*Trisopterus luscus*) formed large schools. While there was some variability in perceived abundance and species between video transects, the sheltered crevices and overhangs appeared to have a greater variety and density of organisms than more exposed surfaces of the hard substrates.

### 3.2. Population structure: Growth rate, age, colony size and branching pattern

**Fig 3:**
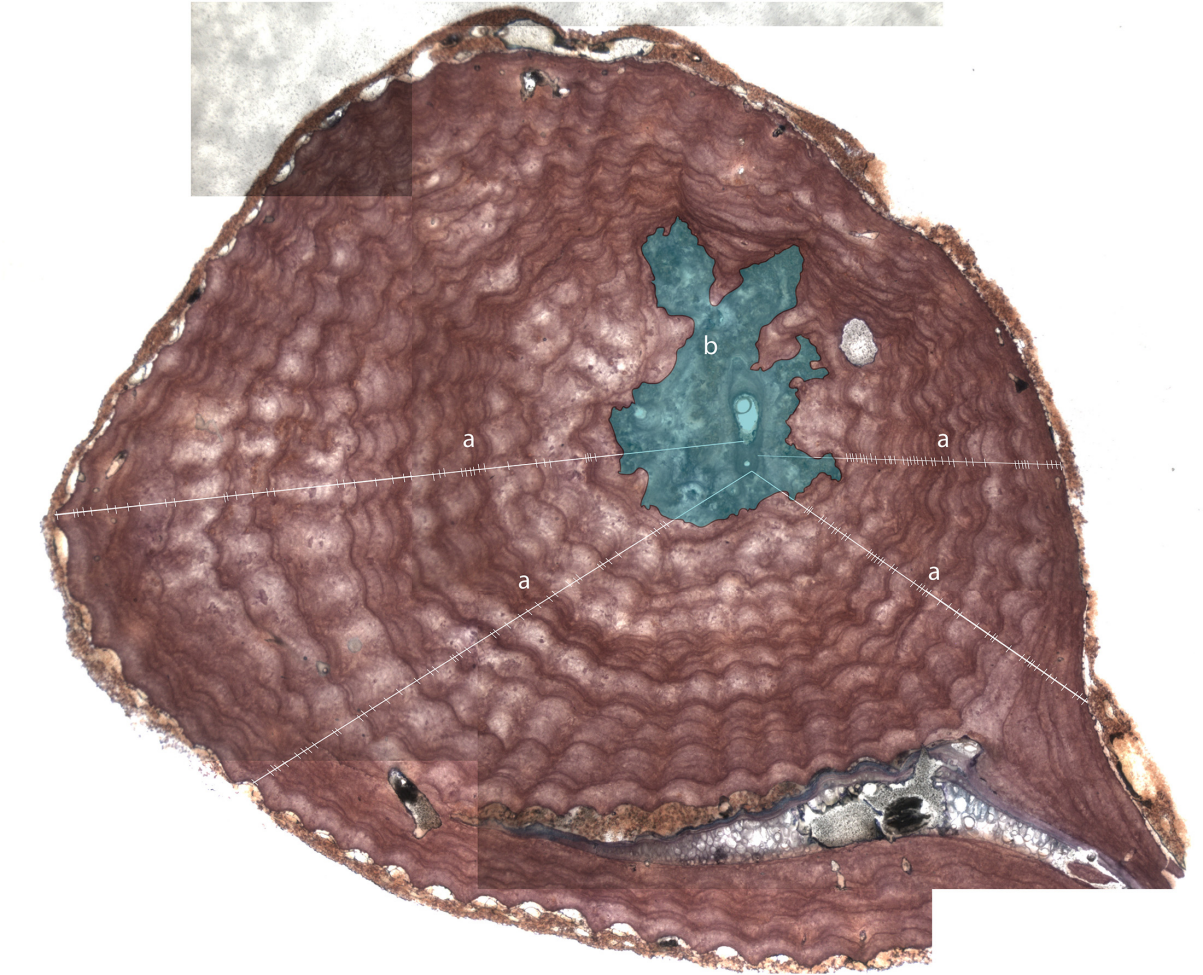
Section of a *Corallium rubrum* colony after decalcification and staining [38]. **a)** Lines corresponding to counting transects. Each dash indicates a circular ring and corresponds to a year. **b)** Medullar region. Image composed by five high-resolution photographs. This colony has an estimated age of 43.4 (± 5.37) years.

**Fig 4:**
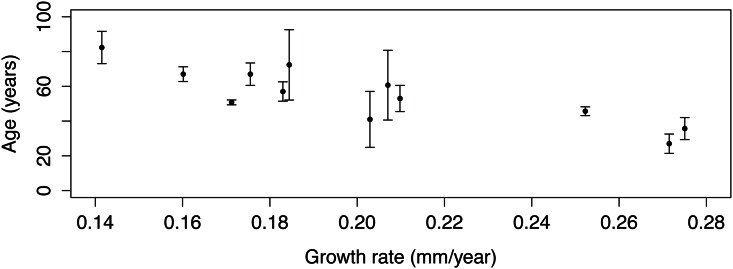
Relationship between age and annual basal diameter growth rate of *Corallium rubrum* from 12 colonies collected off Portugal (70-90m depth). Error bars represent standard deviation.

The estimated average growth rate obtained after growth rings count (Fig 3) was 0.23 ± 0.06 mm year^-1^ (mean ± SD, *n* = 12) but varied between 0.15 and 0.32 mm year^-1^ for individual colonies. A significant negative linear correlation was found between the estimated age and the colony annual growth rate (Pearson *r* = -0.85; *n* = 12; *p* <0.001; Fig 4), indicating a reduction of growth rate in older colonies.

**Fig 5:**
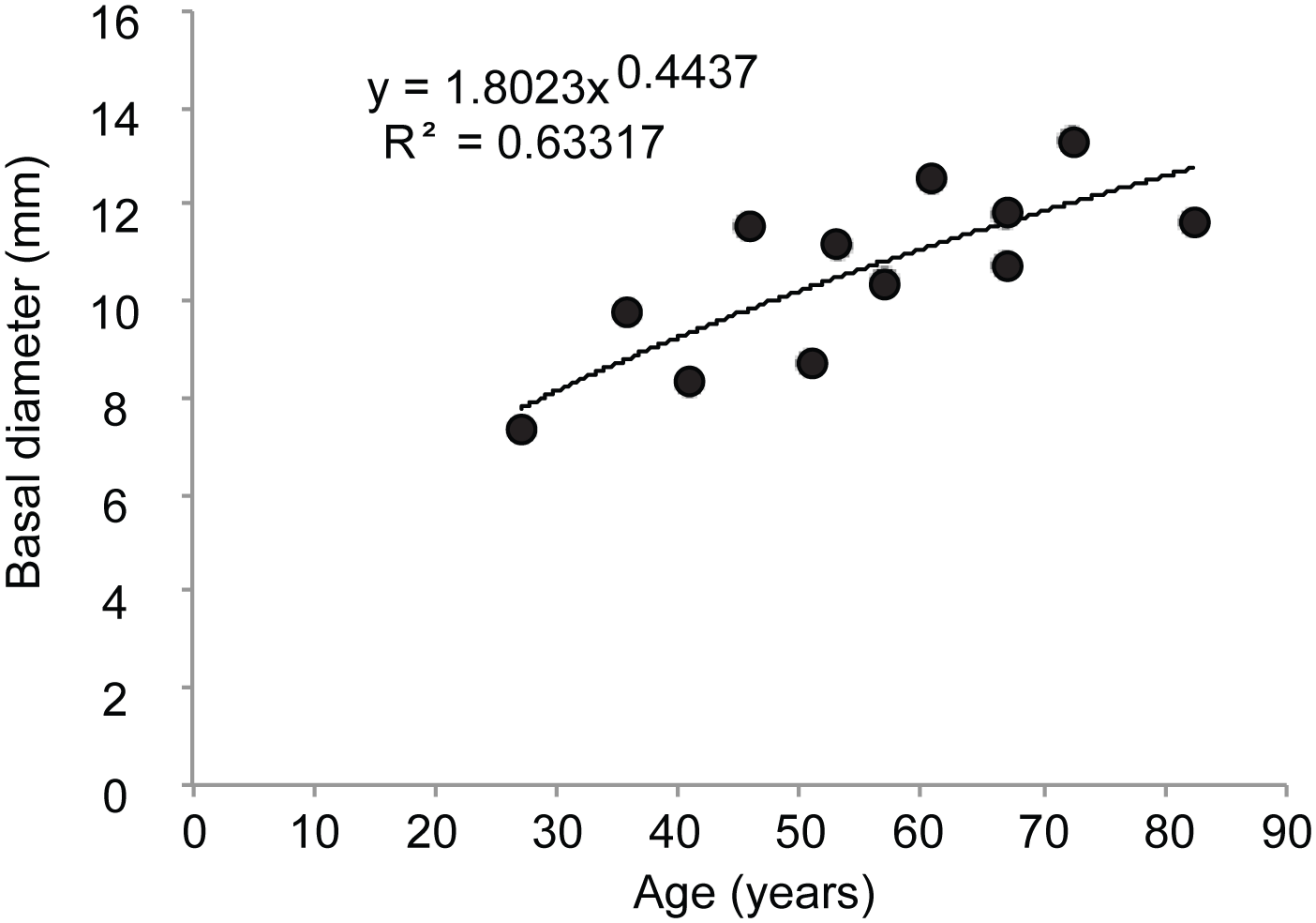
Relationship between estimated age and basal diameter of Atlantic populations of *Corallium rubrum* (*n* = 12).

**Fig 6:**
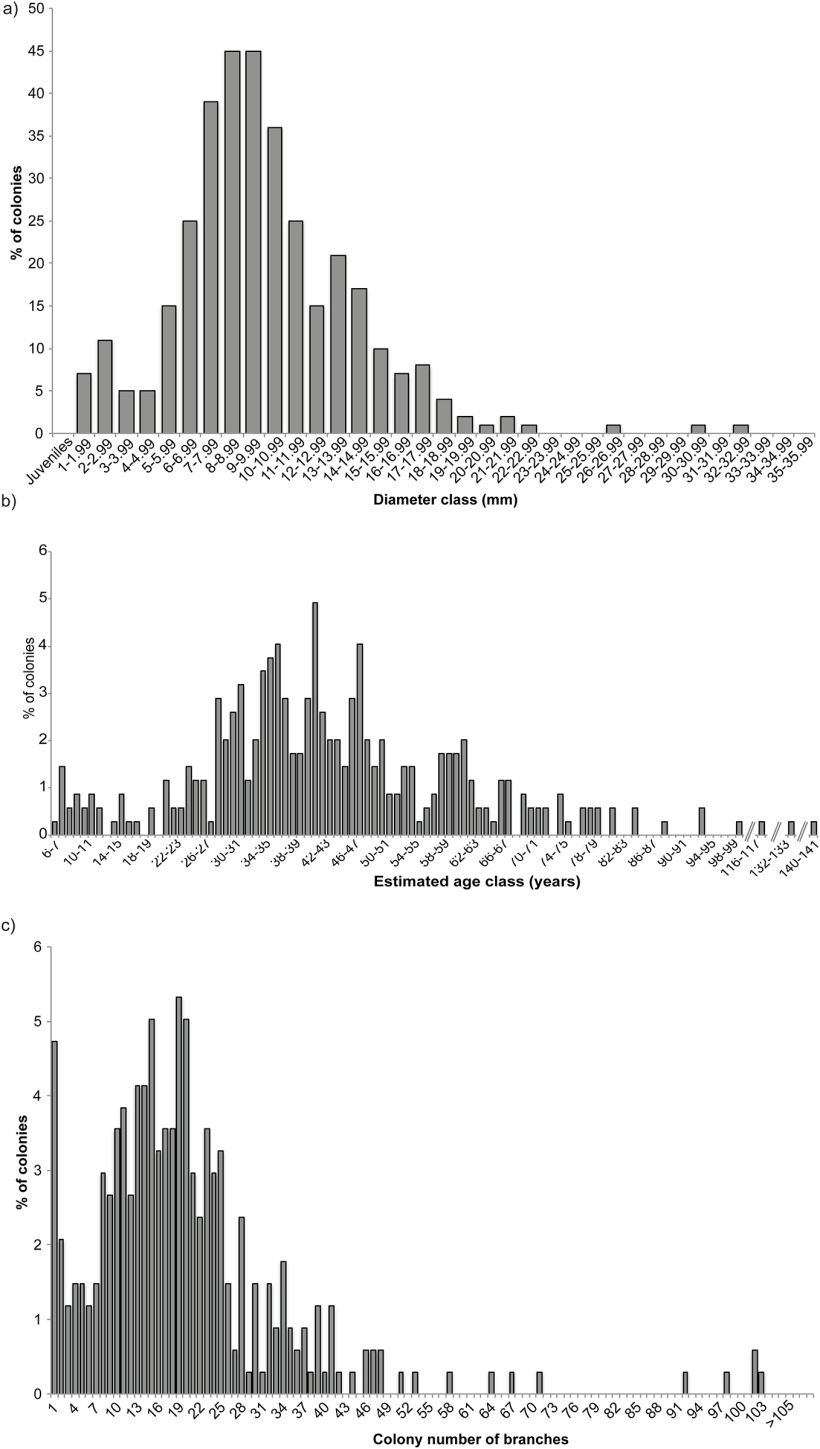
Biometric data from *Corallium rubrum* (SW Portugal, Atlantic). **a)** Size (basal diameter) and **b)** estimated age distributions of dry red coral colonies from the Atlantic (MP samples; *n* = 184). Juveniles (colonies with basal diameter <1 mm) were not included (see section 2.3). **c)** Frequency of the total number of branches in each red coral colony used in a) and b).

The average basal diameter of the dry coral sample was 10.6 ± 5.18 mm (mean ± SD; *n* = 184). Over 80% of the sample was above the 7 mm minimum legal harvestable size established by the General Fisheries Commission for the Mediterranean [34]. Based on the linear relationship between age and basal diameter (Fig 5) applied to the entire dry sample, the minimum and maximum age in the whole sample of dry colonies was 6.7 and 140.5 years, respectively (*n* = 184). The size and age distributions (Fig 6a and 6b) showed a significant positive skew due to the presence of very large and old colonies (skewness = 0.77, *z* = 3.99, *p*<0.001), but this also reflects a harvesting selectivity towards colonies larger than 1 mm. Very small colonies (juveniles < 1 mm and size classes < 6 years in Fig 6a and 6b) were very difficult to measure due to breakage, so the left section of the distribution curves likely does not represent the true population structure. From this sample 90% of colonies appeared to exceed 70 years of age.

*In situ* basal diameter measurements revealed that the most representative red coral colonies at sampling stations along the video-transects were on average 0.79 ± 0.525 mm, corresponding to recruits of about three years of age, and that the largest colony measured in the sampling stations was only 2.1 mm, estimated to be about nine years old. At Reef 4, the shallowest where red coral was discovered (60 m depth), only a few small (0.55 ± 0.212 mm of basal diameter; *n* = 2) red coral colonies were observed amongst a coral garden composed by *S*. *savaglia* (height: mean 65.7 ± 16.64 cm, maximum 87 cm; width: 51.5 ± 15.63 cm, maximum 70 cm) and *P*. *clavata* (height: mean 26.8 ± 4.12 cm, maximum 35 cm; width: mean 20.3 ± 1.97, and maximum 24 cm).

Although highly underestimated due to severe breakage from handling, the branching pattern of dry colonies (Fig 6c) indicated that most had about 20 branches. The branch number distribution is positively skewed (skewness = 2.07, *z* = 7.97, *p*<0.001) due to the presence of highly complex colonies (>60 branches). The high frequency of colonies with only one branch represents colonies damaged by handling.

### 3.3. Atlantic—Mediterranean genetic differentiation

**Fig 7:**
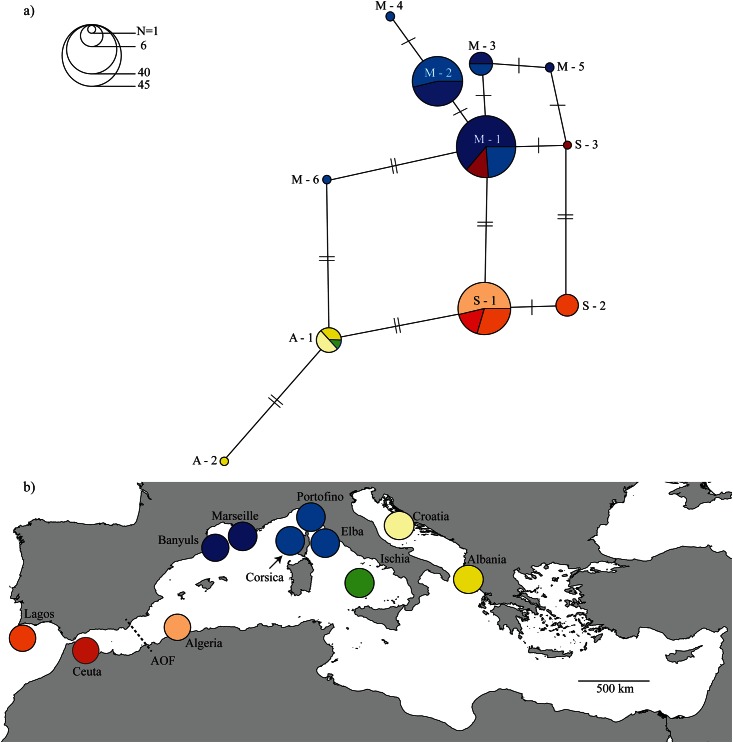
*Corallium rubrum* phylogeography. **a)** Haplotype network of the red coral with Atlantic and Mediterranean data (see Table 1). Haplotype identifiers are included and dashes refer to mutation sites. Size of circles indicates the number of sequences in each haplotype (also see Table 1). **b)** Haplotypes are color-coded according to sampling sites; size of circles in b) is not related to the number of sequences in each haplotype. AOF—Almeria-Oran Front.

There is a clear segregation of red coral populations across the Mediterranean and Atlantic regions forming three main groups of haplotypes. The Northwestern Mediterranean group includes six haplotypes (M-1 to M-6 in Fig 7a) found in Catalonia (Banyuls), Gulf of Lion (Marseille) and Ligurian Sea (Corsica, Portofino and Elba). The eastern group includes two haplotypes (A-1 and A-2) found in the Tyrrhenian (Ischia) and Adriatic (Croatia and Albania) Seas. The southern Mediterranean group corresponds to haplotypes S-1 to S-3, which are found in populations from Algeria, the Alboran Sea (Ceuta at the Gibraltar Strait) and Atlantic (Lagos).

Haplotype A-1 is present in deep (>100 m) populations of Southern Italy and in the Adriatic Sea. Haplotype S-1 shares two mutations with the A-1 haplotype from the Adriatic Sea and this one shares two mutations with haplotype M-6 from Elba in Northwest Italy, whereas it is more distant (4 mutations) from Northwestern Mediterranean haplotypes (M-1 to M-5). The most frequent haplotype from the Gulf of Lion (M-1) is shared with a southern, distant location: Ceuta (Alboran Sea). One Ceuta haplotype (S-3) is closer to haplotypes from the Gulf of Lion (M-1 and M-5) than to the Southern haplotypes (S-1 and S-2). One third (6) of Atlantic colonies display a private haplotype (S-2).

### 3.4. Threats

**Fig 8:**
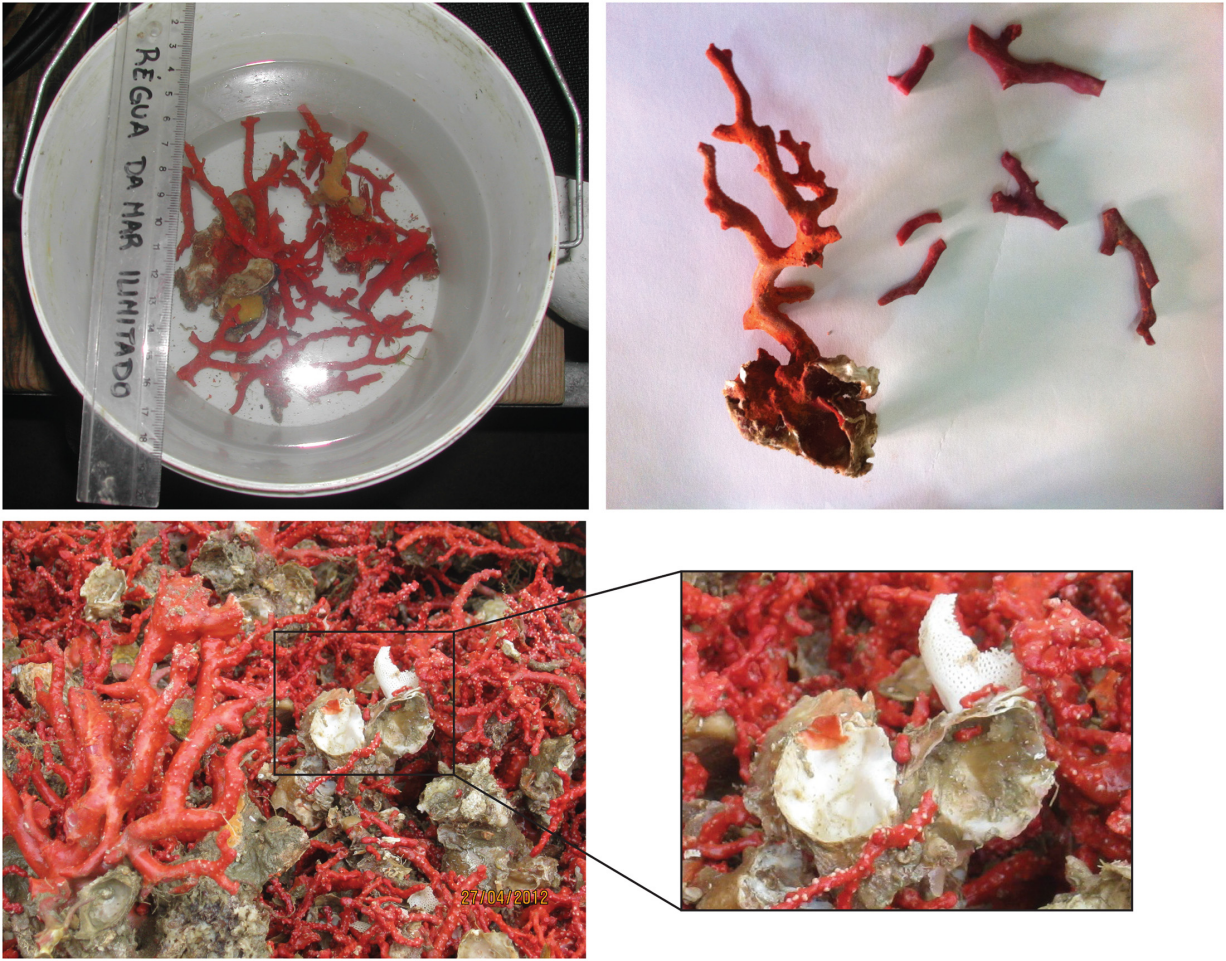
Precious red coral discarded by fishermen using nets (a and b, 2013) and illegal catch from poachers (c and d, 2012). **a)** Live fragments with substrate and **b)** dry fragments and colony with substrate still attached. **c)** Part of the live red coral catch apprehended by the Portuguese Maritime Police off the Algarve coast. Most coral colonies were collected with the oyster (*Neopycnodonte cochlear*) shell substrates attached. **d)** Arrows highlight three of the hundreds of recruits growing over the oyster shells that were the coral substrates. Photo credits: a by Nuno Padrão; b by Joana Boavida; and c by Portuguese Maritime Police.

Lost fishing nets and cables were observed in all video-transect sites, with a minimum of two lost fishing gears per site. Nets and cables were observed entangled on the top and reef flanks, including over corals (Fig 2b). Some nets presented sediment and several epibionts, such as hydroids, indicating non-recent entanglements. Red coral fragments and entire colonies with the substrates still attached are part of the invertebrate by-catch of southern Portuguese fisheries that operate with bottom or near-bottom nets (Fig 8a and 8b). Intact corals are sometimes kept for trophies, but most are discarded at sea or upon reaching the harbor during boat and net cleaning up. All coral colonies that were observed with an attached substrate had numerous recruits, particularly the enormous illegal catch from 2012 (Fig 8c and 8d).

## Discussion

Our study reveals the geographical and local distribution, population structure, associated community, and genetic uniqueness of the westernmost populations of precious red coral *Corallium rubrum*, outside the Mediterranean Sea. We also review all the historical evidence for the ancient presence of the species in SW Iberia, and its collapse likely associated to directed fisheries centuries ago. Besides, we report the current threats to these populations and highlight the need for conservation action.

The population biology parameters of SW Iberian red coral, namely growth rate, size and age structure, and population genetic traits, attest to its distinctness from the widely studied Mediterranean populations. We discovered high benthic diversity in deep-dwelling red coral reefs in the Atlantic, including many habitat-forming species with different biogeographical affinities. Our baseline status assessment reveals relatively undamaged red coral habitat, restricted in space to rocky reefs occurring along a muddy seabed, containing large and structurally complex colonies of very old red corals that co-occur with many other species. We report the high threat posed by illegal coral poachers and by fishing gears that operate near the bottom. Both coral poaching and by-catch remain unquantified in total impact per year, although in one single morning before being caught, a single boat had already harvested 32 kg (the MP sample used in this study, see also below).

### 4.1. Population structure and comparison with Mediterranean

Our biometric data shows that differences of deep red corals from SW Iberia relative to corals from the Mediterranean are striking: the deep population in the Atlantic appears to be in an almost unspoiled condition when compared with the Mediterranean Sea. However, there is no previous baseline for a point of comparison at these depths, despite the ancient records of overexploitation of red coral centuries ago, in shallow depths where it cannot be presently found. It is likely that the difficult access to these very deep sites ensured some natural protection against harvest. Yet, no rigorous benchmark is available for comparison and the known cases of poaching of large quantities of corals in these sites reveal that despite their much more mature population state in comparison with the Mediterranean, the present baseline is not a pristine undisturbed state.

The most frequent basal diameters, a measure highly correlated with age, is up to five-fold higher in SW Iberia compared to Mediterranean populations (Atlantic: 8–10.6 mm, this study; *versus* e.g., mean Spain: 4.8 mm [46]; mode Corsica: 2 mm [47]; mode Italy: 4–6 mm, 50–130 m depth [7, 35]). Over 80% of the Atlantic red coral colonies studied presented basal diameters above the Mediterranean's minimum legal harvest size of 7 mm, whereas even deep Mediterranean populations in good conditions present about 50% above that size [35]. Even corals growing in semi-protected areas in the Mediterranean present less than half the maximum number of branches than SW Iberian corals (42 [47]; *versus* >100 branches, this study). These studies show that currently, even deep-water populations in the Mediterranean are being homogenized towards communities dominated by smaller and simpler colonies. It is obvious from the abundant red coral literature that implementing MPAs is not enough for this slow growing species due to illegal catch [5]. Mediterranean MPAs with no harvesting or fishing activities present much smaller and younger colonies than colonies from the Atlantic (maximum basal diameters <15 mm and 110 years old [8]; versus 32 mm and 140 years, this study). Nowadays, only inaccessible deep populations (hence naturally more protected from illegal harvest) in the Mediterranean Sea still present complex branch architectures and colonies of 15 cm height, comparable to the ones found in the Atlantic [5, 7, 15, 35].

Dramatic shifts in population demographic traits can happen fast. For example in the most productive exploited population in the Mediterranean Sea (Cap de Creus, NE Spain), shallow corals in the 1980s showed a population size structure with mean basal diameter larger than the harvest limit of 7 mm; twenty years later only the nearby deep (>50 m) reefs presented corals >7 mm [5, 48]. In contrast with the fast rates of demographic decline, recovery takes a very long time. The very slow growth rate of the basal diameter appears to be relatively similar throughout its distribution range, including across depth. It has been shown to vary between 0.23–0.35 mm year^-1^ (this study; [7, 8, 38]) and it may slow even more for older corals, as suggested by the negative correlation between age and growth rate. A reduction of the rate at which corals grow as they age appears to be a common trait across species [8, 49–51]. Even in relatively well-preserved deep-water populations in the Mediterranean, few red coral colonies reach more than half a century (in depths comparable to this study [7] report only two between 69 and 93 years and [35] report one with a basal diameter of 24 mm likely some centuries old). Complex branching patterns, which greatly contribute to habitat-engineering, also take a long time to build. Red coral takes 22 years to develop only eight branches, a maximum recorded in Mediterranean caves [52]. Even accounting for the inherent sampling bias towards the largest colonies made by the poachers, the bell-shaped size distribution in Fig 6 together with the presence of substrates attached to the corals, point to a harvesting not so selective (probably due to the extreme depth, reduced bottom time and overall risk of the dive). If this sample is representative of the whole population, then as much as 30% of the SW Iberia red coral assemblage could have over half a century of age, figures only matched by descriptions of historical Mediterranean populations.

Recent studies have highlighted the importance of large coral size for the resistance and resilience of populations. Priori et al. [7] confirm that fecundity increases with colony size and age, meaning that very large corals are the main contributors for the reproductive output of a population. This is even more significant for self-seeding populations because they are more unlikely to have recruitment from other locations. Others have shown that smaller, younger, corals may be more sensitive to ocean acidification [53] and that population growth is far more sensitive to adult survival than to rates of reproduction and recruitment [54–58]. Hence, shifting population size structures towards smaller sizes, an unavoidable consequence of harvesting, leaves coral populations in a vulnerable stage for longer periods of time.

Aggressive harvesting intensities have proved to cause drastic shifts in shallow and deep populations of red corals throughout the Mediterranean [5, 8] and in other exploited organisms around the world (e.g., [12, 27]). Harvested coral populations, whether depleted or not, are dominated by small, young corals that present a very simple architecture. Changes in population size structure and tridimensional complexity of habitat-forming species following disturbances can cause sharp reductions in colony fertility, available reproductive biomass and reduction of associated species richness (e.g., [59]).

The large size of the Atlantic red coral colonies here reported indicates some degree of protection relative to the state of this species in the Mediterranean. This might be related to their deep and marginal location and typically more adverse conditions for diving (higher turbidity, storms, strong water currents, lower temperatures) relative to the Mediterranean Sea. Yet, with the rapid advance of underwater technologies and deep diving safety (e.g., ROVs, rebreathers, deep mixed-gas diving), deep reefs are no longer inaccessible. There is risk of a fast and dramatic decline of this unique population, mirroring the historical "boom and bust" harvests that appear to be responsible for the lack of reports of this species over the last three centuries (see Box 1).

Because populations of *C*. *rubrum* from different locations and depths may exhibit distinct demographic and genetic characteristics [5, 7, 52, 60–61], management strategies for the species should take into account these differences [8].

### 4.2. Zoogeography

Most of the sampled taxa associated with the present Atlantic red coral populations have broad Atlantic-Mediterranean ranges and some present boreal affinities (e.g., *Geodia* spp., *Reteporella* cf. *grimaldii*; [62–63]), suggesting that the red coral assemblage in SW Iberia is inhabited by a unique mixture of fauna from colder (north) and warmer (south and Mediterranean) regions, brought by the different water masses that join in this region [64–66]. Oceanographical contact zones may be relevant in enhancing benthic diversity [67]. Also, the occurrence of structuring species effectively enhances the associated species richness by promoting habitat by themselves [59, 68].

The cold-water coral *Dendrophyllia cornigera* is typically encountered in the lower mesophotic to upper bathyal zones [69], likely related to surface productivity and transport of food particles [70]. *D*. *cornigera* is not a major contributor to coral abundance but was found always in association with the red coral community, as it is absent from the upper mesophotic zone in Portugal [14, 30]. Benthic fauna composition (accompanying and co-occurring species, S1 Table) of the red coral community is remarkably similar in nearby mesophotic reefs at comparable depths, presenting many passive filter-feeders such as habitat-forming corals and sponges [30]. Given the occurrence of large numbers of suspension-feeding fauna at and between rocky reefs, our observations suggest the delivery of high concentrations of food particles via the nearby Arade river discharge and coastal upwelling [71]. The lost fishing gears observed (see also [72]) suggest that this area is an important fishing ground, but few species of commercial interest were observed. However, the high turbidity during video-surveys likely decreased the chances of recording fish that were either repelled or not attracted by divers.

Our results support the role of red coral as a keystone species (*sensu* [4]), based on the large number of associated species found. Red coral appears to determine the benthic community structure by providing habitat to species that in its absence would either not be present or would have reduced densities (e.g., *D*. *cornigera*, *Reteporella* cf. *grimaldii*, *A*. *mediterraneus*).

### 4.3. Atlantic—Mediterranean genetic differentiation

Populations from the Northwestern Mediterranean show haplotypes distinct from populations of the Atlantic, southern Mediterranean and Adriatic Sea, with an Atlantic-Mediterranean differentiation. However, our preliminary analysis suggests that contact zones occur at the Alboran Sea, at the Gulf of Lion and in Southwest Italy. The Atlantic influence extends into the Mediterranean Sea, going beyond the AOF, possibly along the African coast, as indicated by the mitochondrial haplotype S-1 shared by colonies from the Atlantic, Alboran and East of AOF (Fig 7). The presence of one haplotype from the Gulf of Lion (M-1) in Ceuta (Alboran) suggests there could be some admixture even among distant locations (>1000 km). At the Alboran Sea there are two anticyclonic gyres formed by the different Atlantic and Mediterranean water masses and part of this water flows eastward feeding the Algeria current [73–74]. The analyses of microsatellite data and nuclear intron sequences have suggested a lack of deep divergence across the AOF [75] or a possible introgression at the west of the AOF. Our results could point to differential introgression of vicariant diverged genomic backgrounds from the Atlantic to the Mediterranean Sea [76]. Mitochondrial DNA shows haplotypes that are shared between distant regions and have few mutation sites pointing to a relatively recent divergence of populations from these two regions, as previously reported with the nuclear EF1 [75].

Despite the low resolution of this marker we observe a geographically coherent spatial structure between the Atlantic, the Western and the Eastern Mediterranean, here shown for the first time including red coral from the Atlantic. These results confirm the strong genetic structure and limited gene flow of this key structuring species. Importantly, these data suggest the existence of different genetic units (e.g., a private haplotype from the Atlantic), a topic worth investigating in the future with samples across its entire geographical distribution and with more independent markers.

### 4.4. Threats

Effective Atlantic red coral habitat in SW Iberia appears to be spatially limited to deep (60–100 m depth) rocky reefs interspersed by soft substrate shelf. This Atlantic population is presently very attractive to coral poachers. An apprehension made by the Portuguese MP in April 2012, yielded 32 kg collected by a single diver in one single deep dive (from an apparent recreational boat with a decompression chamber on board). In comparison, the most productive population in the Mediterranean yields only 0.5–2 kg/diver per dive [37] and the red coral fishery on the Atlantic coast of Morocco (from Cape Spartel to Larache) yields on average 3.2–4.2 kg/fishing day per boat [15]. Deep red corals become larger than shallower ones, but in pristine locations corals may become even larger [37]. Our data on basal diameters, age estimation and underwater visual observations indicate that Atlantic red coral assemblages contain many colonies equivalent to the now extremely rare or absent largest specimens ever reported in the Mediterranean red coral literature (c.a. 20 cm S2 Fig; [46, 52]), including colonies >100 years old. A few months (Sept 2012) after the first apprehension of red coral by the Portuguese MP, another apparent recreational boat was caught in the same harvesting activity, indicating that it was not an isolated harvesting case. The illegal harvesting activity here reported for SW Iberia, removed not only a substantial amount of coral, but also the substrates where the corals grow (oysters, brachiopods and some rocks), including numerous recruits (Fig 8 and S2 Fig). The complex morphology of these substrates provides the critically limiting space available for colonization, the major controlling factor of recruitment in red coral [77]. Considering these ongoing impacts, one can hypothesize that the populations that we have studied might have been even denser and larger in the absence of such harvesting. Strong population declines in marine reserves with little police enforcement leave demographic and genetic footprints [78]. Yet, this may be reversed, although slowly, since effective enforcement of protected areas allows colonies to grow older and hence larger [7].

In the Mediterranean the species protection is considered strategic. It is subject to several national and international regulations (minimum sizes for catch set at 7–10 mm, bans on non-selective gear, fishing quotas and closures, limited number of licenses; e.g., Barcelona Convention Annex III), it is listed in Annex V of the European Union Habitats Directive and a proposal to include it on CITES Appendix II was made in 2007 [79]. On the Atlantic part of its distribution only the Habitats Directive is applicable, which excludes African countries (Morocco to Cape Verde). Results presented here underline the importance of this species and call for a reappraisal of the protection required to maintain the reefs in a healthy state.

Litter observed among the deep red coral gardens was composed exclusively by lost fishing gears that were either entangled on prominent structures (corals and rocks) or covering the seabed and fauna. This is consistent with what has been reported from the region. In southern Portugal several types of fishing take place (e.g., pelagic and demersal purse seiners, trammel and gill nets, traps and fish trawling), making lost fishing gear the most abundant type of litter [29, 72, 80]. Deep (mesophotic) reefs are increasingly recognized for the role they play in local fisheries. On one side they sustain local fishers especially once the shallow reefs are depleted; on the other, deeper reefs replenish some commercial (and non-commercial) species to the adjoining shallower reefs [81–82]. Yet, fisheries that contact the bottom have the potential to devastate large areas of structural biogenic habitat, such as coral gardens [32, 83–86].

Notwithstanding the slow growth and impacts upon the precious red coral since historical times, its relatively young age at sexual maturity (2–9 years [87, 88]) is a likely reason for the species not being closer to extinction despite the extensive harvesting [47]. However, synergies between diverse sources of impact are known to lead to more dramatic population declines [89]. Harvesting added to increased frequency of heat waves in the future may cause extreme reductions of coral density [8]. Ocean acidification, which has been increasing in North Atlantic waters for three decades [90], affects red and cold-water corals [53, 91]. The species is particularly susceptible to acidified conditions due to the much higher solubility of its Mg-rich calcite skeleton compared to calcite or aragonite, which typically form skeletons of calcified organisms (e.g., scleractinian corals and bryozoans [92]). Since several other species colonize the substrate where it settles, there is a strong risk of sharp reductions in density resulting in local extinctions. Furthermore, range edge populations, like the Atlantic red coral, may present high and distinct genetic diversity [93], providing invaluable genomic resources for species adaptation to future environmental changes.

## Conclusions

Marine conservation faces the paradigm of knowing what should be done yet being unable to implement it effectively. The historical past shows that recovery from damage in these unique Atlantic red coral populations might take centuries. Immediate and effective protection measures are needed to ensure its conservation, including enforcement. Specifically, we suggest the classification of SW Iberian red coral sites within the Natura 2000 framework, which aims to harmonize uses and values of an area, and its inclusion in a MPA. This can be achieved by regulating fishing activities that operate at the coral sites, such as imposing a minimal distance from the rocky patches. Despite uncertainties, protecting this unique Atlantic red coral is certainly the first step to take.

## Supporting Information

S1 Table Taxa observed in the Atlantic red coral (*Corallium rubrum*) benthic assemblage. (PDF)

S1 Fig Temperature and conductivity profiles kindly provided by Project Baseline during the Global Underwater Expedition 2014 (http://projectbaseline.org; http://globalsubdive.com/expeditions). (PDF)

S2 Fig Images of large precious red coral (*Corallium rubrum*) colonies from the Atlantic population. **a)** ROV view of large (c.a. 15 x 20 cm) colony off SW Iberia; the two laser lights correspond to 5 cm. **b)** Largest red coral colony obtained from the illegal collection off Lagos-Portimão (Site A, SW Iberia, Atlantic). This approx. 20 cm height colony presented all branches broken, so this is likely an underestimation of the actual height. **c)** View of the 32 kg red coral catch in 2012 by the Portuguese Maritime Police apprehended between Reefs 1–3 in Site A (Fig 1). Photo credits: a) CCMAR; b) Nelson Coelho; c) Portuguese newspaper Correio da Manhã 02 November 2014.
(PDF)

S3 Fig Nineteenth century engraving representing a coral fishery using the St. Andrew's Cross fishing device, also known as *ingegno*. From this representation it appears that fishermen would dive to the sea bottom to collect the coral suggesting a harvest restricted to shallow depths. Source: Drassana magazine from the Barcelona Maritime Museum No. 2 1994 (with permission); Credits: R. Prudêncio (http://blog-de-historia.blogspot.pt/2008/02/pesca-do-coral-em-portugal.html).
(JPG)

S1 File Image-based taxa catalog from an Atlantic red coral assemblage off Portugal. Names correspond to names in the manuscript. Whenever possible a description of major characteristics was included. Images edges present distortion of the field of view caused by the wide-angle camera and motion. Arrows in the images indicate the taxa of interest. Images were extracted from the underwater video frames obtained during field surveys in the Global Underwater Expedition 2014 (http://projectbaseline.org; http://globalsubdive.com/expeditions) in collaboration with project Deep Reefs (http://www.deepreefs.com). Document has 44 pages (>100MB). Can be found at http://www.figshare.com under Doi: 10.6084/m9.figshare.1544524. URL: https://figshare.com/s/8350279a5bb411e5b61406ec4b8d1f61.
(PDF)

## Abbreviations

AOF: Almeria-Oran Front
CCMAR: Center of Marine Sciences, University of Algarve
DOV: Diver-operated video
DPV: Diver propulsion vehicle
GUE: Global Underwater Explorers
MPA: Marine Protected Area
MP: Maritime Police
ROV: Remotely operated vehicle
