# Correction: A Well-Kept Treasure at Depth: Precious Red Coral Rediscovered in Atlantic Deep Coral Gardens (SW Portugal) after 300 Years

**DOI:** 10.1371/journal.pone.0150654

**Published:** 2016-02-26

**Authors:** Joana Boavida, Diogo Paulo, Didier Aurelle, Sophie Arnaud-Haond, Christian Marschal, John Reed, Jorge M. S. Gonçalves, Ester A. Serrão

There are errors in the Funding section. The correct funding information is as follows: This research was supported with funding from the Inaqua Conservation Fund by Oceanário de Lisboa and National Geographic Channel through project Deep Reefs (http://www.deepreefs.com), a National Geographic/Waitt grant (no. W153-11), MeshAtlantic project 2013 (AA ERDF 2009-1/110), OCEANA (Expedition Portugal 2011), EXCL/AAGGLO/0661/2012 (Fundação para a Ciência e a Tecnologia), additional support for field expenses by Project Baseline/Global Underwater Expedition 2014. JB was supported by a doctoral grant (SFRH/72501/2010) awarded by “Fundação para a Ciência e a Tecnologia” (FCT) and JMSG by UID/Multi/04326/2013 (Fundação para a Ciência e a Tecnologia). The funders had no role in study design, collection, analysis, interpretation of data, writing and in the decision to publish.
