# Supplementary material for: A Well-Kept Treasure at Depth: Precious Red Coral Rediscovered in Atlantic Deep Coral Gardens (SW Portugal) after 300 Years
Source: PLoS One. 2016 Jan 22;11(1):e0147228. doi: 10.1371/journal.pone.0147228 (PMC4730840; doi:10.1371/journal.pone.0147228)
Supplement: S1 Table — (PDF) [file pone.0147228.s001.pdf]

Supporting Information

A well-kept treasure at depth: Precious red coral rediscovered in Atlantic deep coral gardens (SW Portugal) after 300 years

Joana Boavida, Diogo Paulo, Didier Aurelle, Sophie Arnaud-Haond, Christian Marschal, John Reed, Jorge MS Gonçalves, Ester A Serrão

S1 Table. Taxa observed in the Atlantic red coral (*Corallium rubrum*) benthic assemblage.

Species or Taxa	Group
**Characteristic species of Atlantic red coral communities (Reefs 1-3)**	
Corallium rubrum (Linnaeus, 1758)	Anthozoa
Dendrophyllia cornigera (Lamarck, 1816)	Anthozoa
Reteporella cf. *grimaldii* (Julien, 1903)	Bryozoa
**Accompanying species**	
Axinella sp. (Schmidt, 1862)	Porifera
Axinellidae (Carter, 1875)	Porifera
cf. *Geodia* sp. (Bowerbank, 1858)	Porifera
cf. *Pachastrella monilifera* (Schmidt, 1868)	Porifera
cf. *Poecillastra compressa* (Bowerbank, 1866)	Porifera
cf. *Tedania (Tedania) urgorri* (Cristobo, 2002)	Porifera
cf. *Tethya* sp. (Lamarck, 1815)	Porifera
Demospongiae (Sollas, 1885) 1	Porifera
Demospongiae (Sollas, 1885) 2	Porifera
Demospongiae (Sollas, 1885) 3	Porifera
Demospongiae (Sollas, 1885) 4	Porifera
Demospongiae (Sollas, 1885) 5	Porifera
Demospongiae (Sollas, 1885) 6	Porifera
Demospongiae (Sollas, 1885) 7	Porifera
Desmacidon fruticosum (Montagu, 1814)	Porifera
Phakellia robusta (Bowerbank, 1866)	Porifera
Phakellia ventilabrum (Linnaeus, 1767)	Porifera
Stylocordyla cf. *pellita* (Topsent, 1904)	Porifera
Hydrozoa (Owen, 1843)	Hydrozoa
Halecium halecinum (Linnaeus, 1758)	Hydrozoa
Aglaopheniidae (Marktanner-Turneretscher, 1890)	Hydrozoa
Alcyonium glomeratum (Hassal, 1843)	Anthozoa
Alcyonium sp. (Linnaeus, 1758)	Anthozoa
Dendrophyllia ramea (Linnaeus, 1758)	Anthozoa
Eunicella sp. (Verrill, 1869)	Anthozoa
Eunicella verrucosa (Pallas, 1766)	Anthozoa
Paramuricea clavata (Risso, 1826)	Anthozoa
Parazoanthus axinellae (Schmidt, 1862)	Anthozoa
Sabella sp. (Linnaeus, 1767)	Polychaeta
Sabella spallanzanii (Gmelin, 1791)	Polychaeta
Serpulidae (Rafinesque, 1815)	Polychaeta
Palinurus elephas (Fabricius, 1787)	Crustacea
Neopycnodonte cochlear (Poli, 1795)	Bivalvia
Brachiopoda (Duméril, 1805)	Brachiopoda

Myriapora truncata (Pallas, 1766)	Bryozoa
Astrospartus mediterraneus (Risso, 1826)	Echinodermata
Centrostephanus longispinus (Philippi, 1845)	Echinodermata
Echinaster (Echinaster) sepositus (Retzius, 1783)	Echinodermata
Holothuria (Panningothuria) forskali (Delle Chiaje, 1823)	Echinodermata
Holothuria sp. (Linnaeus, 1767)	Echinodermata
Marthasterias glacialis (Linnaeus, 1758)	Echinodermata
Ascidiacea (Nielsen, 1995)	Tunicata
Halocynthia papillosa (Linnaeus, 1767)	Tunicata
Acantholabrus palloni (Risso, 1810)	Pisces
Anthias anthias (Linnaeus, 1758)	Pisces
Actinopteri	Pisces
Scorpaena sp. (Linnaeus, 1758)	Pisces
Serranus cabrilla (Linnaeus, 1758)	Pisces
Trisopterus luscus (Linnaeus, 1758)	Pisces
**Co-occurring species**	
Alcyonium palmatum (Pallas, 1766)	Anthozoa
cf. Alcyoniina	Anthozoa
cf. *Veretillum cynomorium* (Pallas, 1766)	Anthozoa
Ellisella paraplexauroides Stiasny, 1936	Anthozoa
Eunicella filiformis (Studer, 1879)	Anthozoa
Octocorallia (Haeckel, 1866)	Anthozoa
Bonellia viridis (Rolando, 1821)	Echiura
Clupeidae (Cuvier, 1817)	Pisces
Diplodus vulgaris (Geoffroy Saint-Hilaire, 1817)	Pisces
**Characteristic species of gold coral communities (Reef 4)**	
Savalia savaglia (Bertoloni, 1819)	Anthozoa
cf. *Geodia barretti* (Bowerbank, 1858)	Porifera
Pawsonia saxicola (Brady & Robertson, 1871)	Echinodermata
