# Supplementary material for: A Well-Kept Treasure at Depth: Precious Red Coral Rediscovered in Atlantic Deep Coral Gardens (SW Portugal) after 300 Years
Source: PLoS One. 2016 Jan 22;11(1):e0147228. doi: 10.1371/journal.pone.0147228 (PMC4730840; doi:10.1371/journal.pone.0147228)

Supporting Information

A well-kept treasure at depth: Precious red coral rediscovered in Atlantic deep coral gardens (SW Portugal) after 300 years

Joana Boavida, Diogo Paulo, Didier Aurelle, Sophie Arnaud-Haond, Christian Marschal, John Reed, Jorge MS Gonçalves, Ester A Serrão

S1 Fig. Temperature and conductivity profiles. Kindly provided by Project Baseline during the Global Underwater Expedition 2014 (http://projectbaseline.org; http://globalsubdive.com/expeditions).

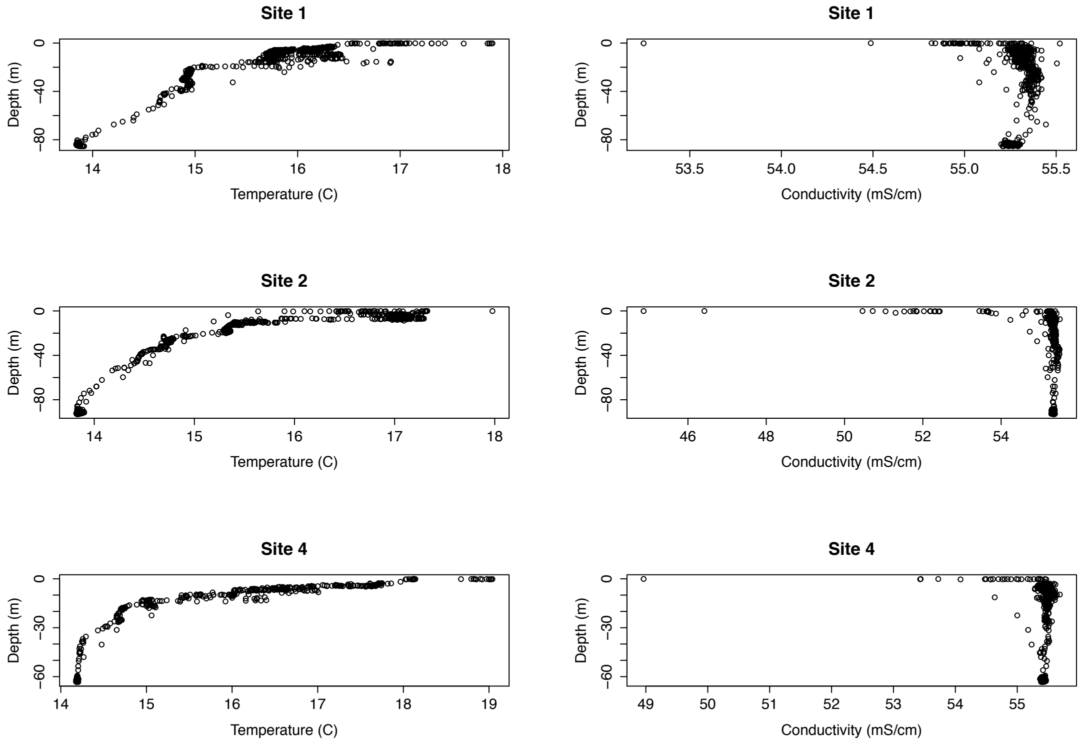

Supplement: S1 Fig — (PDF) [file pone.0147228.s002.pdf]
