# Supplementary material for: A Well-Kept Treasure at Depth: Precious Red Coral Rediscovered in Atlantic Deep Coral Gardens (SW Portugal) after 300 Years
Source: PLoS One. 2016 Jan 22;11(1):e0147228. doi: 10.1371/journal.pone.0147228 (PMC4730840; doi:10.1371/journal.pone.0147228)

# Supporting Information

## A well-kept treasure at depth: Precious red coral rediscovered in Atlantic deep coral gardens (SW Portugal) after 300 years

**Joana Boavida, Diogo Paulo, Didier Aurelle, Sophie Arnaud-Haond, Christian Marschal, John Reed, Jorge MS Gonçalves, Ester A Serrão**

**S2 Fig. Images of large precious red coral (*Corallium rubrum*) colonies from the Atlantic population. a)** ROV view of large (c.a. 15 x 20 cm) colony off SW Iberia; the two laser lights correspond to 5 cm. **b)** Largest red coral colony obtained from the illegal collection off Lagos-Portimão (Site A, SW Iberia, Atlantic). This approx. 20 cm height colony presented all branches broken, so this is likely an underestimation of the actual height. **c)** View of the 32 kg red coral catch in 2012 by the Portuguese Maritime Police apprehended between Reefs 1-3 in Site A (Fig. 1). Photo credits: a) CCMAR; b) Nelson Coelho; c) Portuguese newspaper Correio da Manhã 02 November 2014.

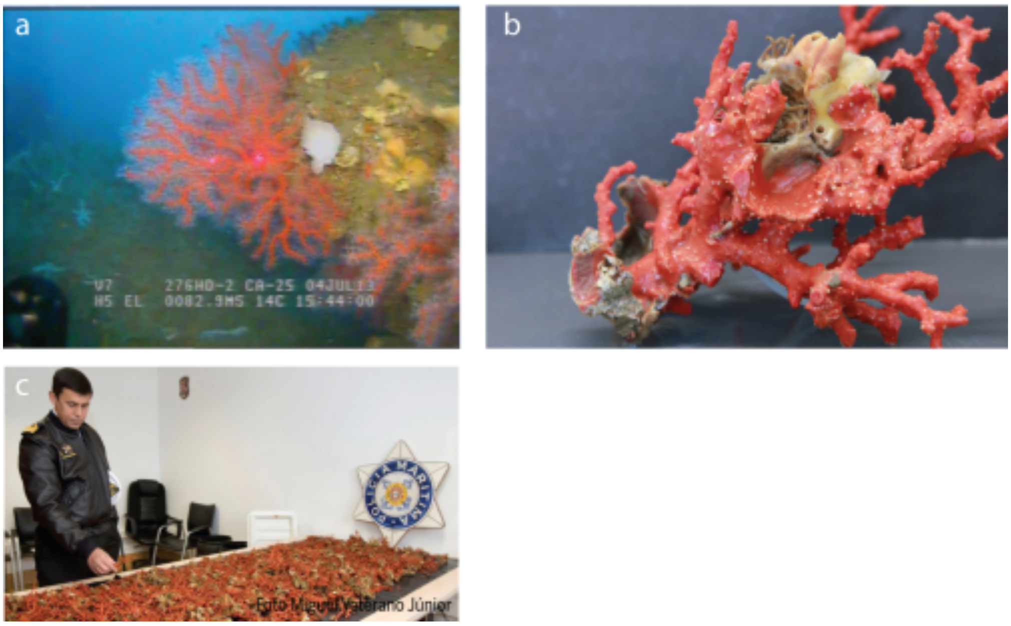

Supplement: S2 Fig — a) ROV view of large (c.a. 15 x 20 cm) colony off SW Iberia; the two laser lights correspond to 5 cm. b) Largest red coral colony obtained from the illegal collection off Lagos-Portimão (Site A, SW Iberia, Atlantic). This approx. 20 cm height colony presented all branches broken, so this is likely an underestimation of the actual height. c) View of the 32 kg red coral catch in 2012 by the Portuguese Maritime Police apprehended between Reefs 1–3 in Site A (Fig 1). Photo credits: a) CCMAR; b) Nelson Coelho; c) Portuguese newspaper Correio da Manhã 02 November 2014. (PDF) [file pone.0147228.s003.pdf]
