# Supplementary figures and images for: A Well-Kept Treasure at Depth: Precious Red Coral Rediscovered in Atlantic Deep Coral Gardens (SW Portugal) after 300 Years
Source: PLoS One. 2016 Jan 22;11(1):e0147228. doi: 10.1371/journal.pone.0147228 (PMC4730840; doi:10.1371/journal.pone.0147228)

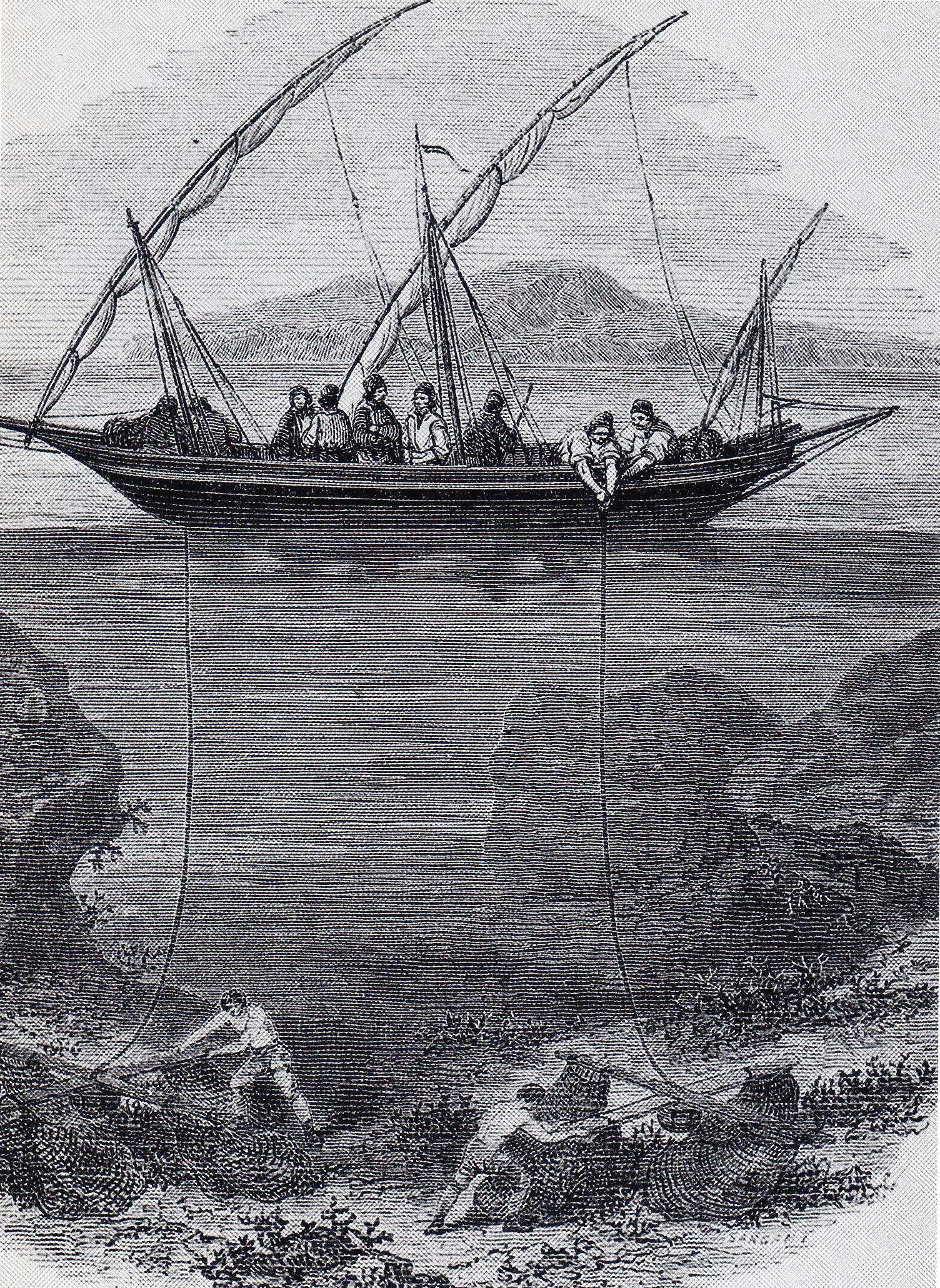

Supplement: S3 Fig — From this representation it appears that fishermen would dive to the sea bottom to collect the coral suggesting a harvest restricted to shallow depths. Source: Drassana magazine from the Barcelona Maritime Museum No. 2 1994 (with permission); Credits: R. Prudêncio (http://blog-de-historia.blogspot.pt/2008/02/pesca-do-coral-em-portugal.html). (JPG) [file pone.0147228.s004.jpg]
