# Supplementary material for: A Well-Kept Treasure at Depth: Precious Red Coral Rediscovered in Atlantic Deep Coral Gardens (SW Portugal) after 300 Years
Source: PLoS One. 2016 Jan 22;11(1):e0147228. doi: 10.1371/journal.pone.0147228 (PMC4730840; doi:10.1371/journal.pone.0147228)

A well-kept treasure at depth: Precious red coral rediscovered in Atlantic deep coral gardens (SW Portugal) after 300 years - Supplementary material S2

Joana Boavida, Paulo D, Aurelle D, Arnaud-Haond S, Marschal C, Reed J, Gonçalves JMS, Serrão EA. 2015

S2 - Image-based taxa catalog from an Atlantic red coral assemblage off Portugal. Names correspond to names in the manuscript. Whenever possible a description of major characteristics was included. Images edges present distortion of the field of view caused by the wide-angle camera and motion. Arrows in the images indicate the taxa of interest. Images were extracted from the underwater video frames obtained during field surveys in the Global Underwater Expedition 2014 (http://projectbaseline.org; http://globalsubdive.com/expeditions) in collaboration with project Deep Reefs (http://www.deepreefs.com).

Table of Contents

Porifera **3**
Astrophorida **3**
cf. *Geodia barretti* 3
cf. *Geodia* sp. 4
Halichondrida **4**
Axinella sp. 4
Axinellidae 5
Phakellia robusta 5
Phakellia ventilabrum 6
Poecilosclerida **7**
Desmacidon fruticosum 7
cf. *Tedania urgorri* 7
Suberitida **8**
Stylocordyla cf. pellita 8
Tethyida **8**
cf. *Tethya* sp. 8
Tetractinellida **9**
cf. *Pachastrella monilifera* 9
cf. *Poecillastra compressa* 10
Unidentified **11**
Demospongiae 1 11
Demospongiae 2 11
Demospongiae 3 12
Demospongiae 4 12
Demospongiae 5 13
Demospongiae 6 13
Demospongiae 7 14

Cnidaria 15
Hydrozoa 15
Hydrozoa/Aglaopheniidae 15
Halecium halecinum 16
Anthozoa - Octocorallia, Alcyonacea 16
Alcyonium glomeratum 16
Alcyonium palmatum 17
Alcyonium sp. 17
Paramuricea clavata 18
Corallium rubrum 18
Eunicella verrucosa / *Eunicella* sp. 19
Eunicella filiformis 19
Ellisella paraplexauroides 20
cf. Alcyoniina 21
Octocorallia 21
Anthozoa - Octocorallia, Pennatulacea 22
Veretillum cynomorium 22
Anthozoa - Hexacorallia, Scleractinia 23
Dendrophyllia cornigera 23
Dendrophyllia ramea 23
Anthozoa - Hexacorallia, Zoantharia 24
Parazoanthus axinellae 24
Savalia savaglia 25

Bryozoa 26
Gymnolaemata 26
Myriapora truncata 26
Reteporella cf. *grimaldii* 27

Annelida 27
Polychaeta 27
Sabella sp. / *Sabella spallanzani* 27
Serpulidae 28

Arthropoda (Crustacea) 28
Malacostraca 28
Pallinurus elephas 28

Mollusca 29
Bivalvia 29
Neopycnodonte cochlear 29

Brachyopoda 30
Brachyopoda 30

Echinodermata 30
Asteroidea 30
Echinaster sepositus 30
Marthasterias glacialis 31
Ophiuroidea 32
Astrospartus mediterraneus 32
Echinoida 33
Centrostephanus longispinus 33
Holothuroidea 34
Holothuria forskali / *Holothuria* sp. 34
Pawsonia saxicola 35

Echiura 36

Echiuroidea **36**
Bonellia viridis 36

Chordata **37**
Ascideacea **37**
Ascidiacea 37
Halocynthia papillosa 37
Actinopteri (Fish) **38**
Acantholabrus palloni 38
Anthias anthias 39
Scorpaena sp. 40
Serranus cabrilla 41
Trisopterus luscus 42
Diplodus vulgaris 42
Actinopteri 43
Clupeidae 44

Porifera

Astrophorida

cf. *Geodia barretti*

Juvenile cf. *G. barretti* in the image below. Presents a distinct osculum on the flat top region. Colour is usually pale to brownish and may present some fine sediment settled on the top surrounding the osculum. Smooth surface. Juveniles are small and relatively rounded. Was only observed in the upper circalittoral from 40 to 60 m depth; absent from the deeper red coral sites. Bottom image shows two specimens collected.

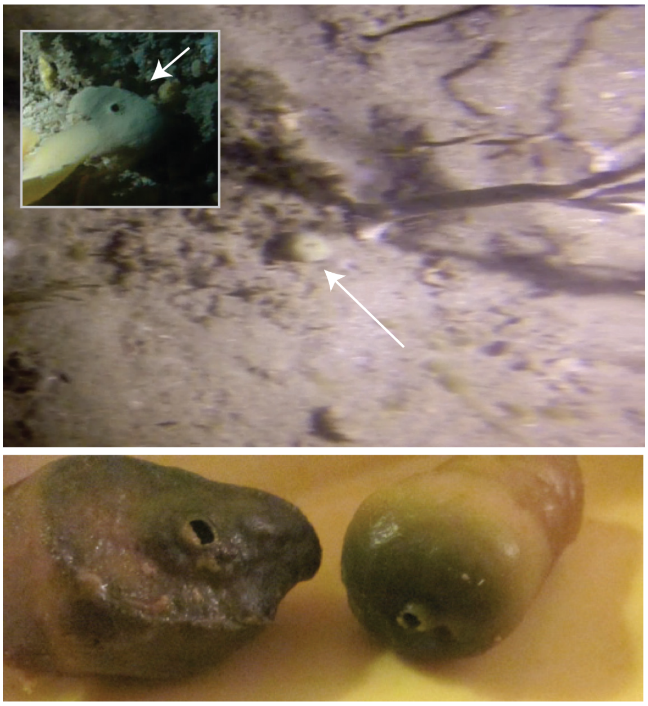

cf. *Geodia* sp.

Massive white sponge.

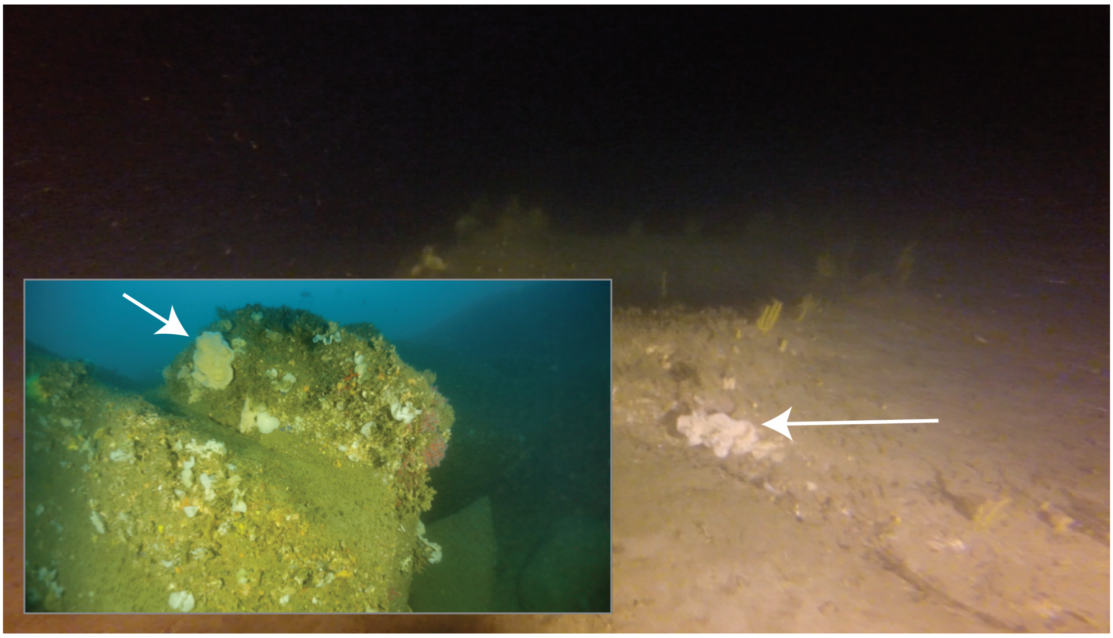

Halichondrida

Axinella sp.

Possibly *A. vaceleti*. This is a small, yellow, stalked fan-shape, sponge. Apical margin of lamina may be frayed or irregularly lobate.

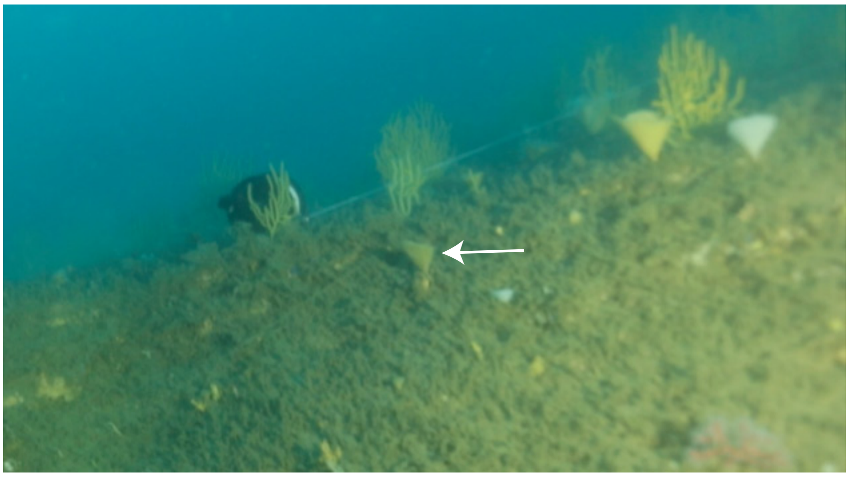

Axinellidae

Unidentified cup sponge with the apical margin of lamina appearing relatively thick and distinctive yellow color. Possibly *A. infundibiliformis*. Common throughout the circalittoral from about 40 m depth down to al least 100 m. About 15 cm tall.

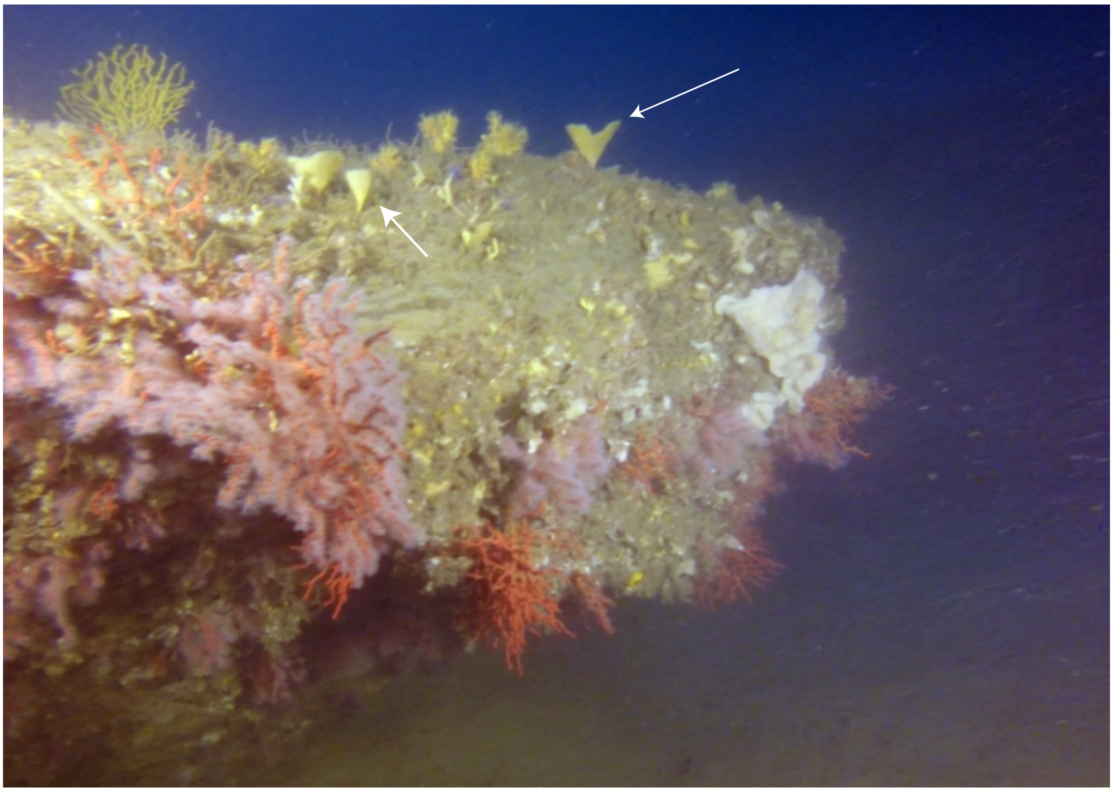

Phakellia robusta

Flabellate, laminate pale sponge with visible "veins".

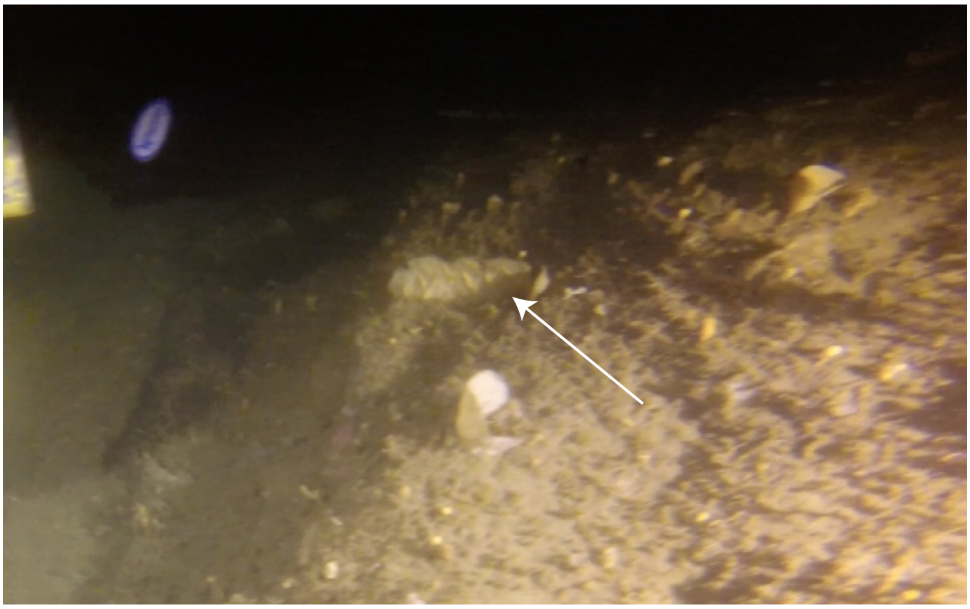

Phakellia ventilabrum

Fan-shape, laminate or cup-shape, pale sponge. Apical margin of lamina are thin and vein-like structures may be visible to the naked-eye (in video imagery). Cup-shaped specimens are stalked. This species has been identified by samples collected previously (cup-shaped only).

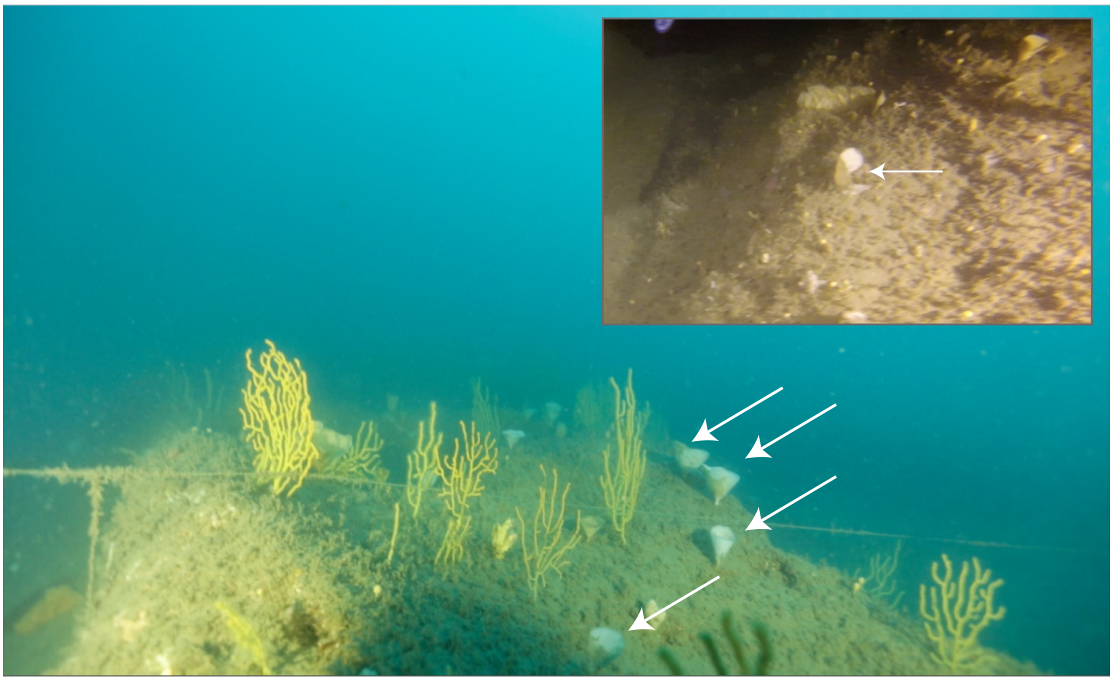

Poecilosclerida

Desmacidon fruticosum

This is a relatively common fisheries by-catch species in the region. Massive pale sponge.

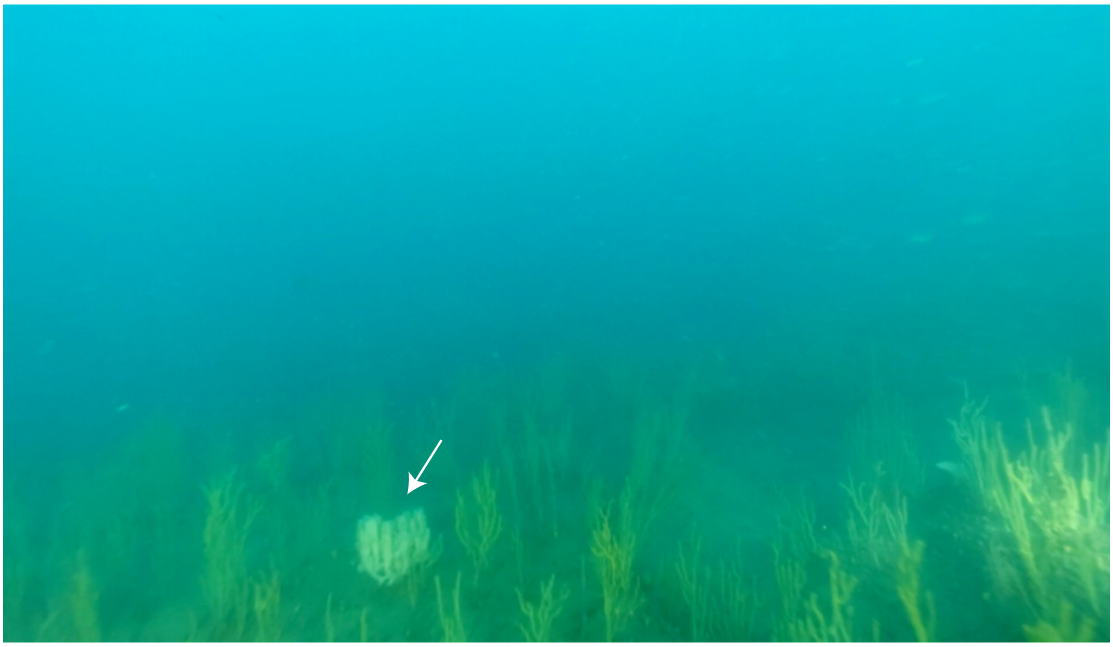

cf. *Tedania urgorri*

Yellow, cup-shape sponge with a thick base; very large.

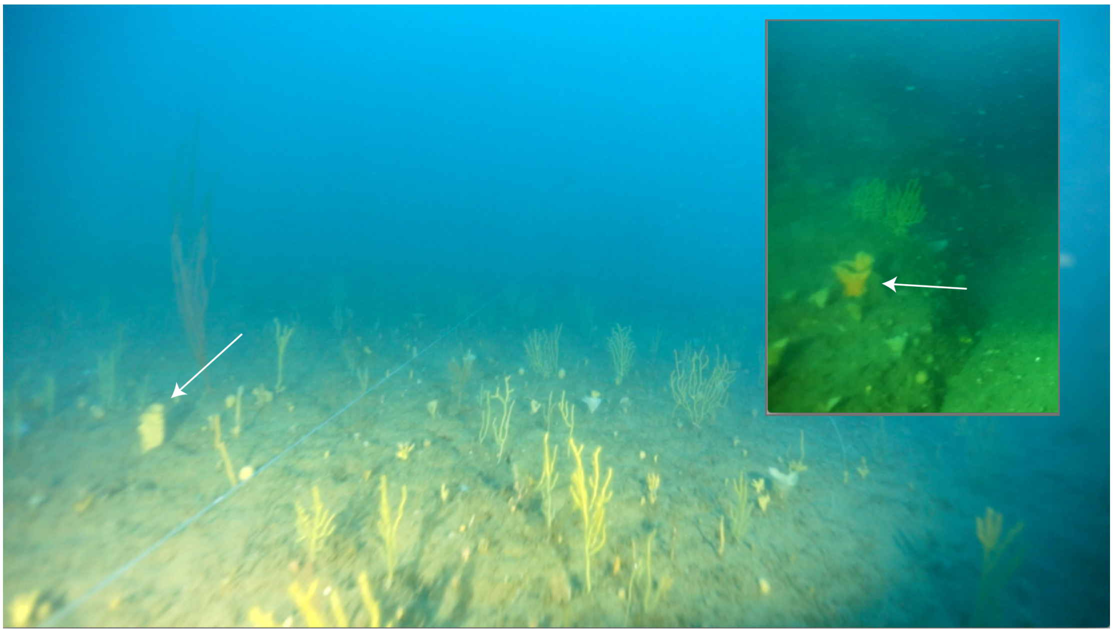

Suberitida

Stylocordyla cf. pellita

Yellow lollipop sponge, small, with delicate stalk.

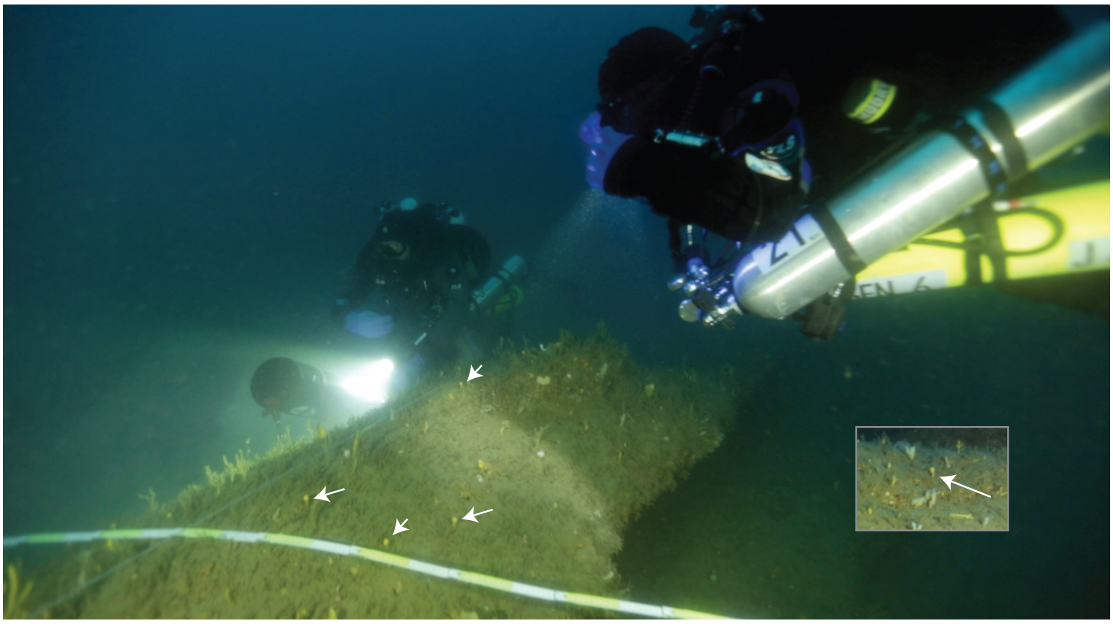

Tethyida

cf. *Tethya* sp.

Small, yellow, spherical sponge with visible oscula.

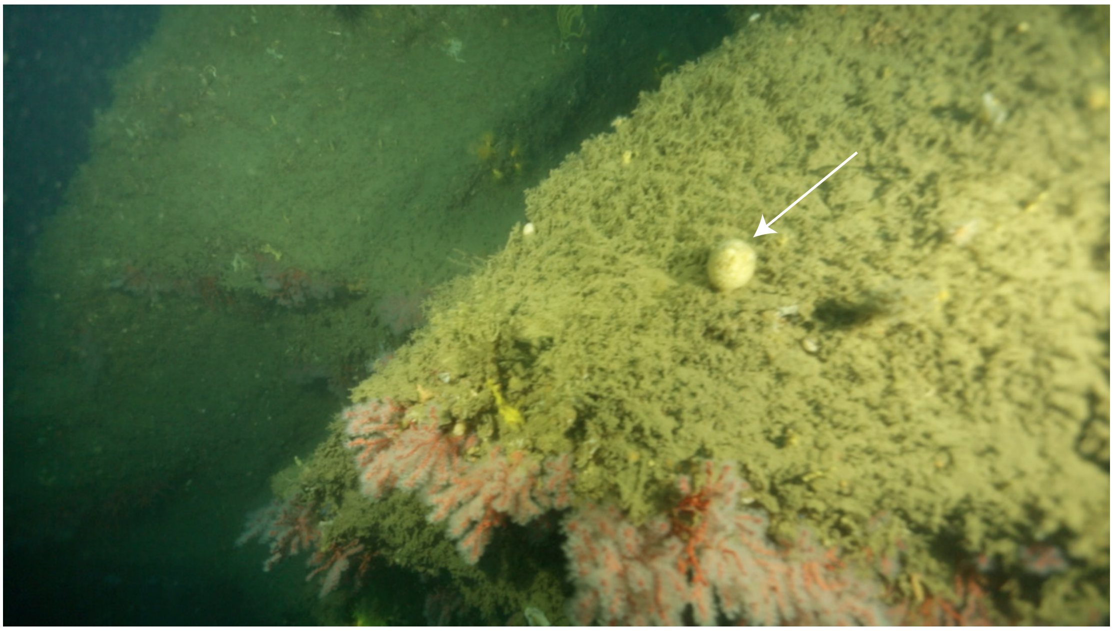

Tetractinellida

cf. *Pachastrella monilifera*

Massive pale sponge with a thick, slightly curled, apical margin of lamina; presents an irregular morphology that may be almost cup-shaped, flabellate or laminate.

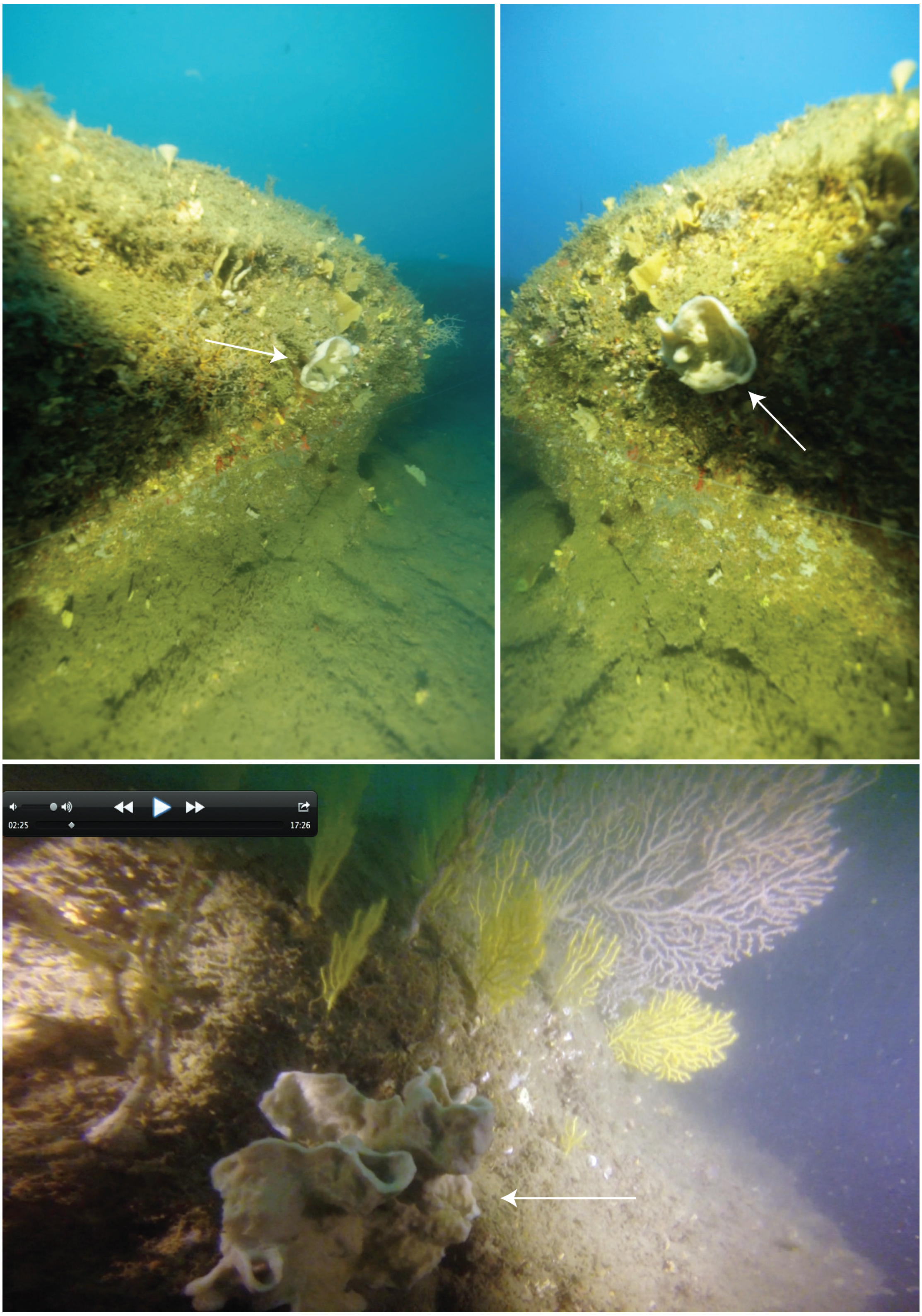

cf. *Poecillastra compressa*

Flabellate, yellowish or orangish sponge. A diver is visible on the upper left of the image and a white transect line goes along the rocky substrate.

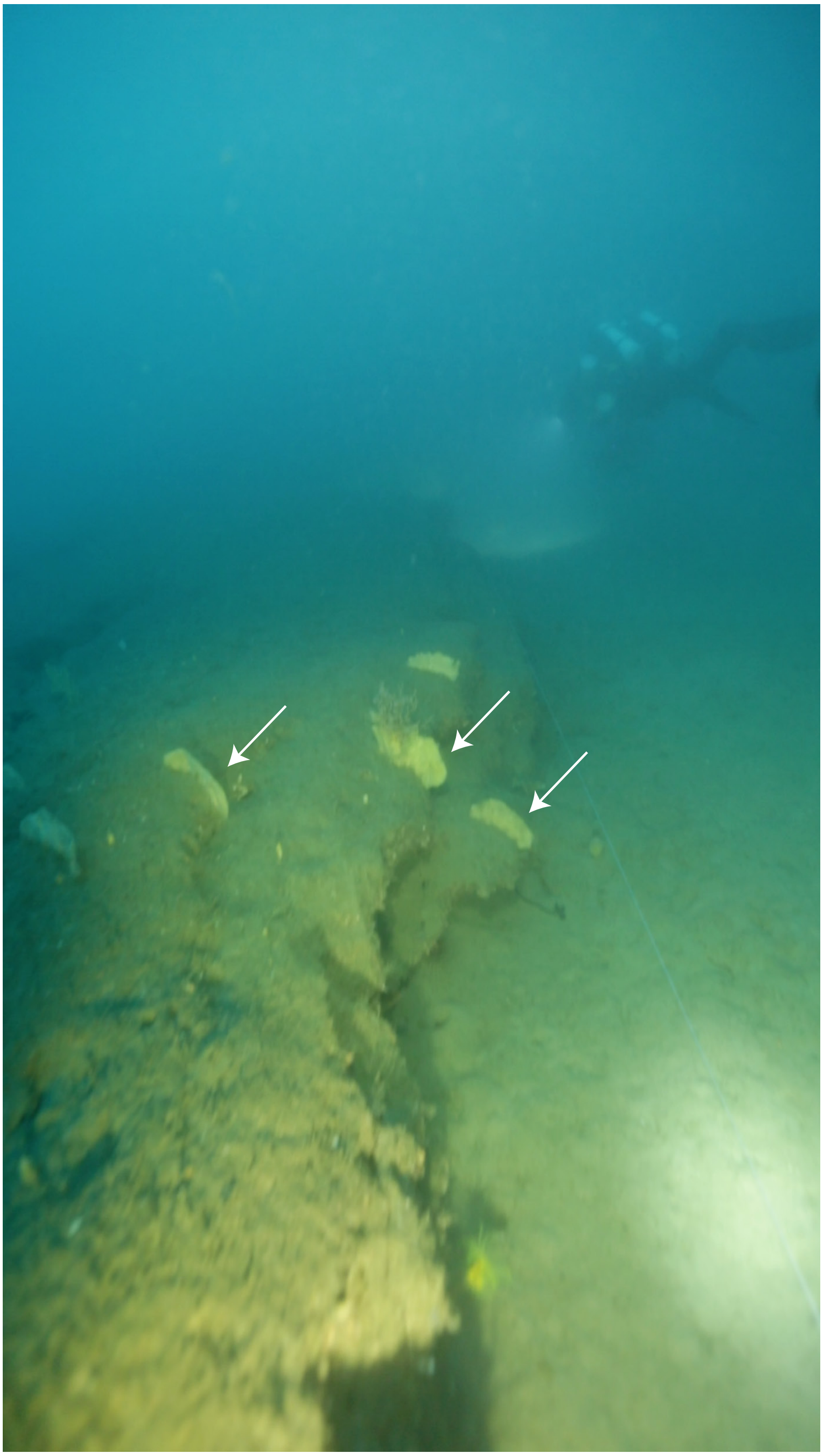

Unidentified

Demospongiae 1

Incrusting orange sponge with visible channels.

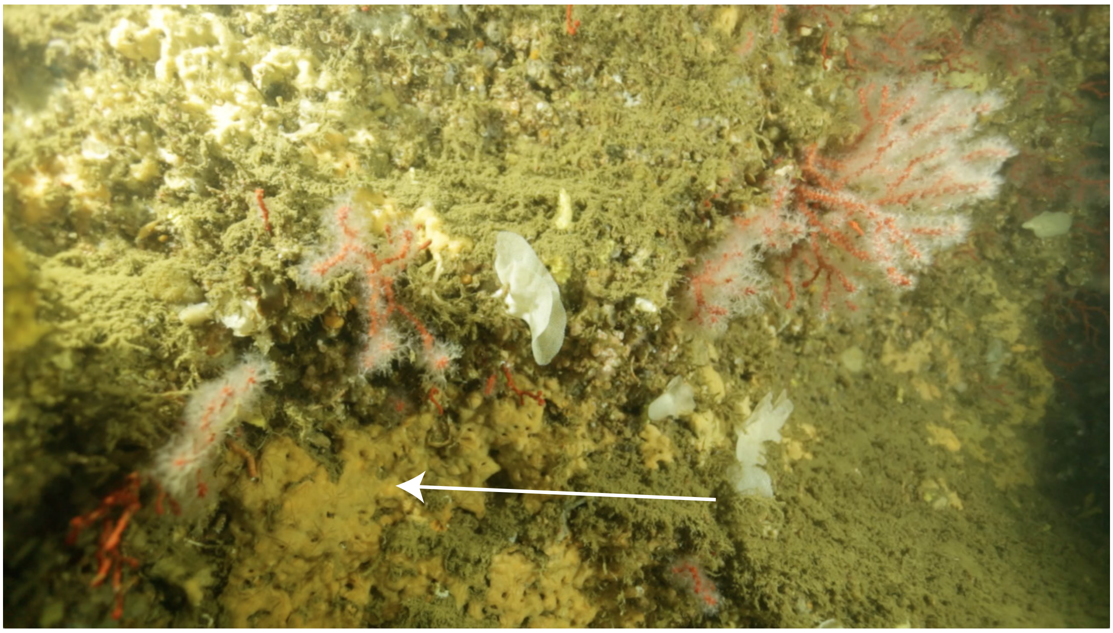

Demospongiae 2

Greyish cup sponge.

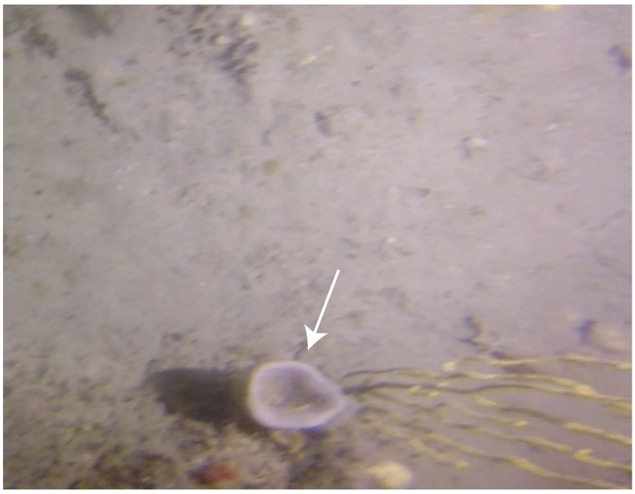

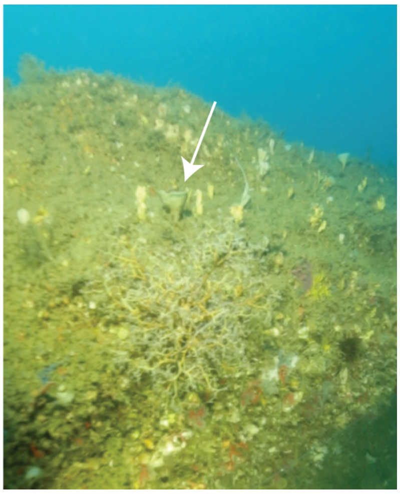

Demospongiae 3

Incrusting purple sponge with visible channels. In the upper left part of the image is a diver.

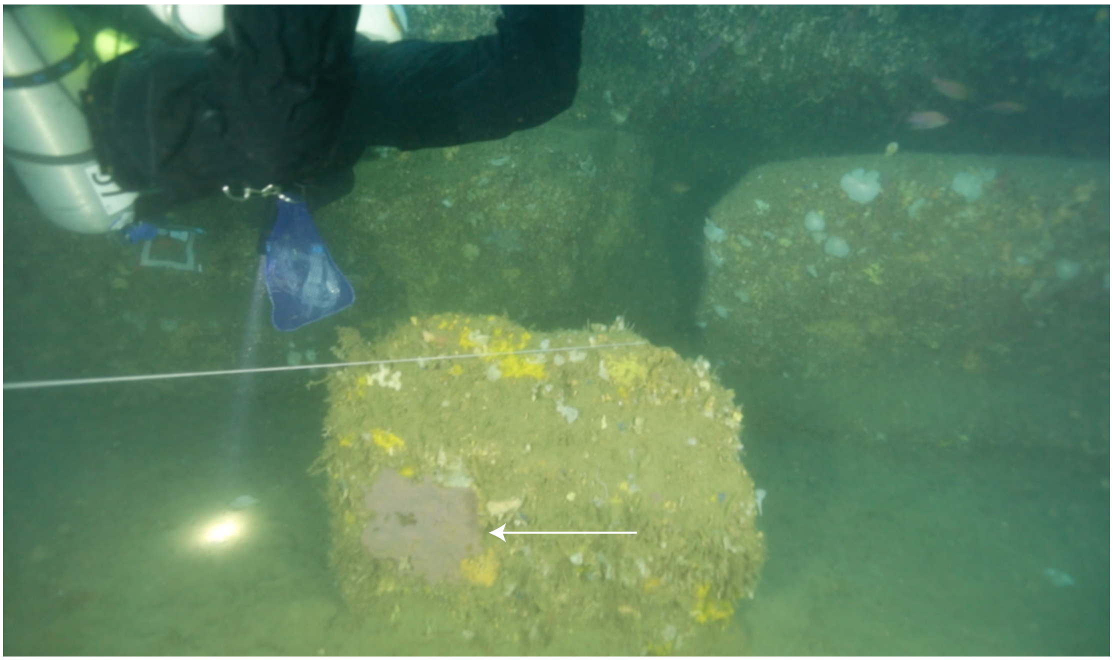

Demospongiae 4

Incrusting white sponge.

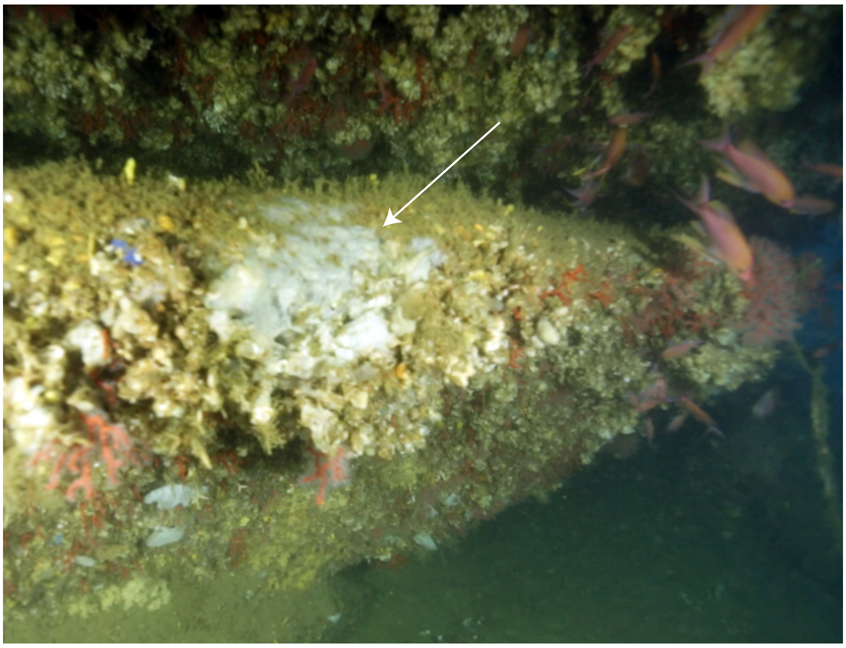

Demospongiae 5

Incrusting yellow sponge with irregular surface. Common.

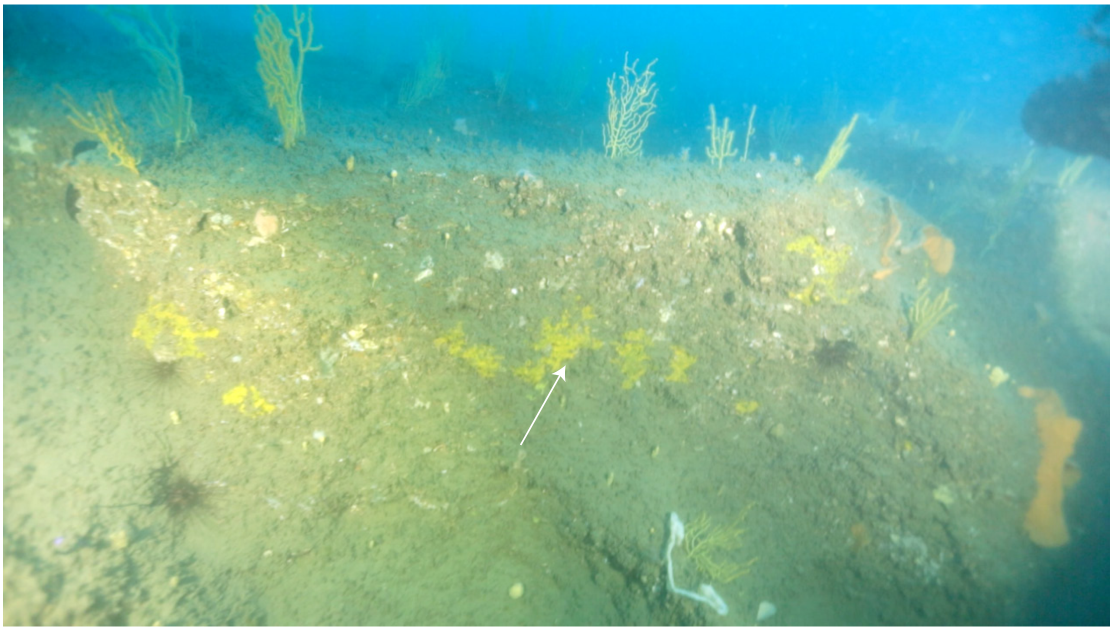

Demospongiae 6

Very large incrusting, pale blueish/purple sponge.

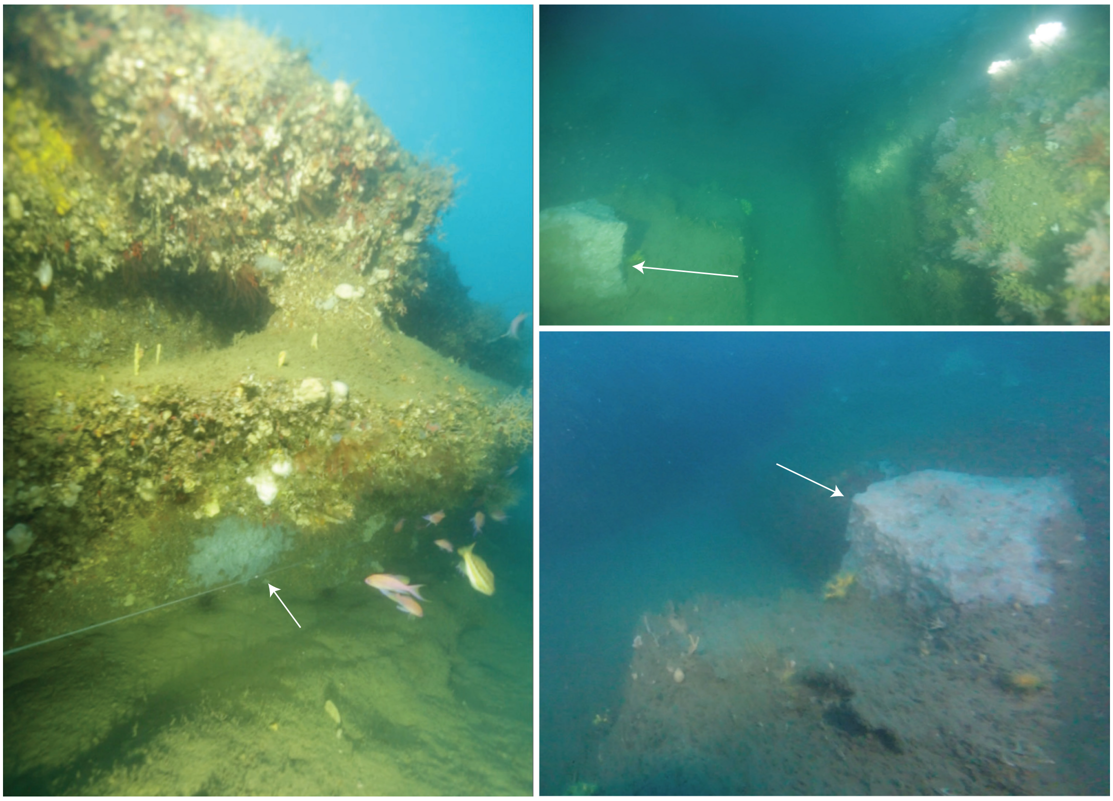

Demospongiae 7

Very large white cup sponge with thick base.

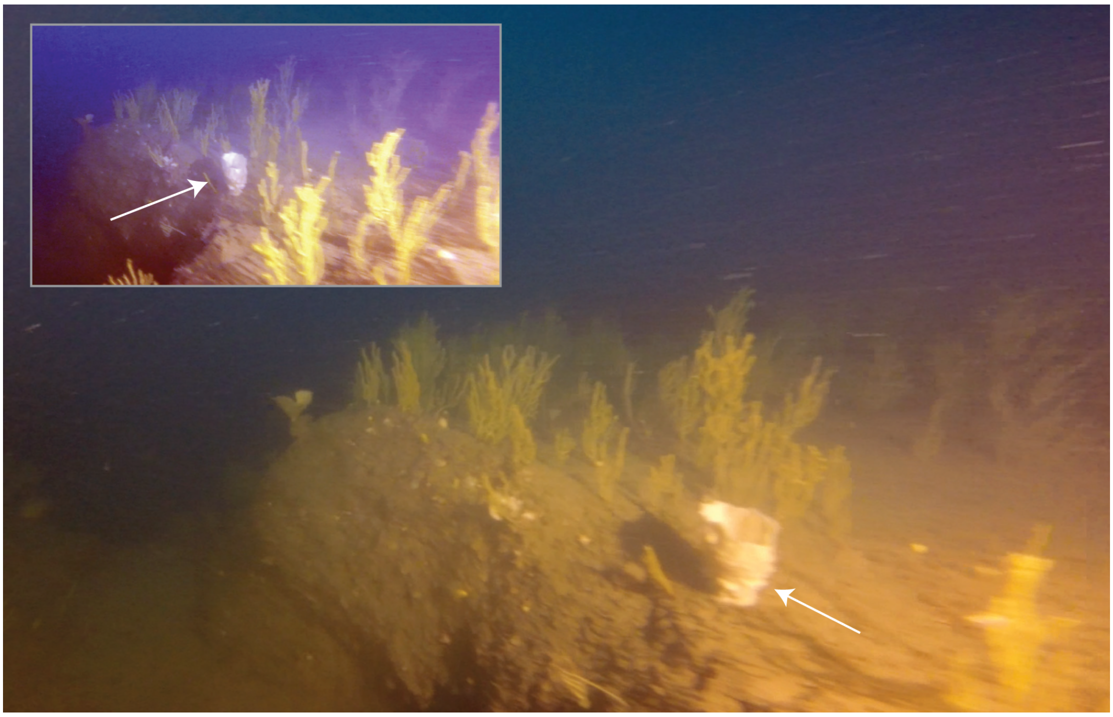

Cnidaria

Hydrozoa

Hydrozoa/Aglaopheniidae

Hydrozoa - Unidentified tall hidroid in dense aggregation (Hy). Aglaopheniidae - Feather-like brownish hidroid (Ag) with distinctive pinnate growth form.

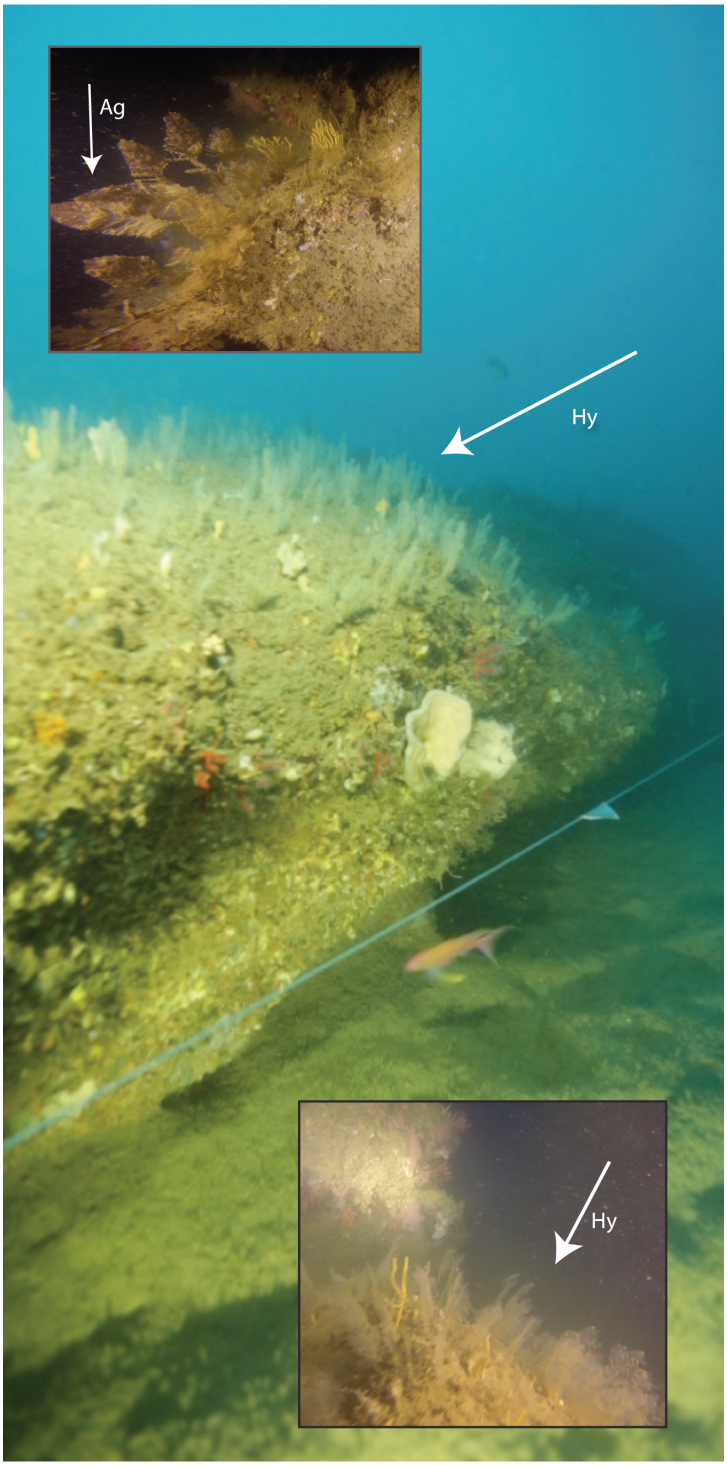

Halecium halecinum

Conspicuous oposed branching and characteristic dusty appearance. Well known species from local fisheries by-catch. May cover almost completely rocky outcrops in the upper circalittoral zone (~40 m). A piece of fishing net entangled over the rock is visible in the lower left side of the right image below.

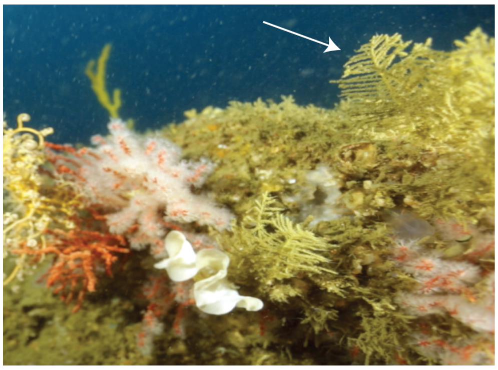

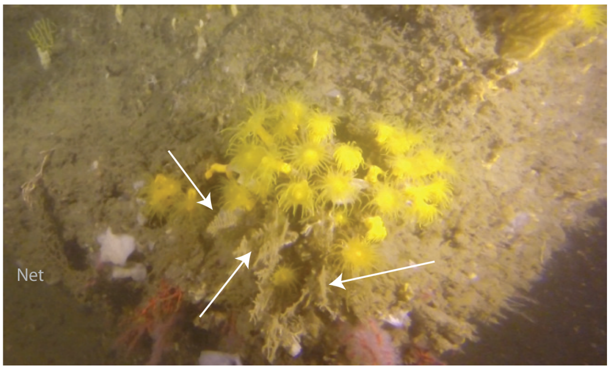

Anthozoa - Octocorallia, Alcyonacea

Alcyonium glomeratum

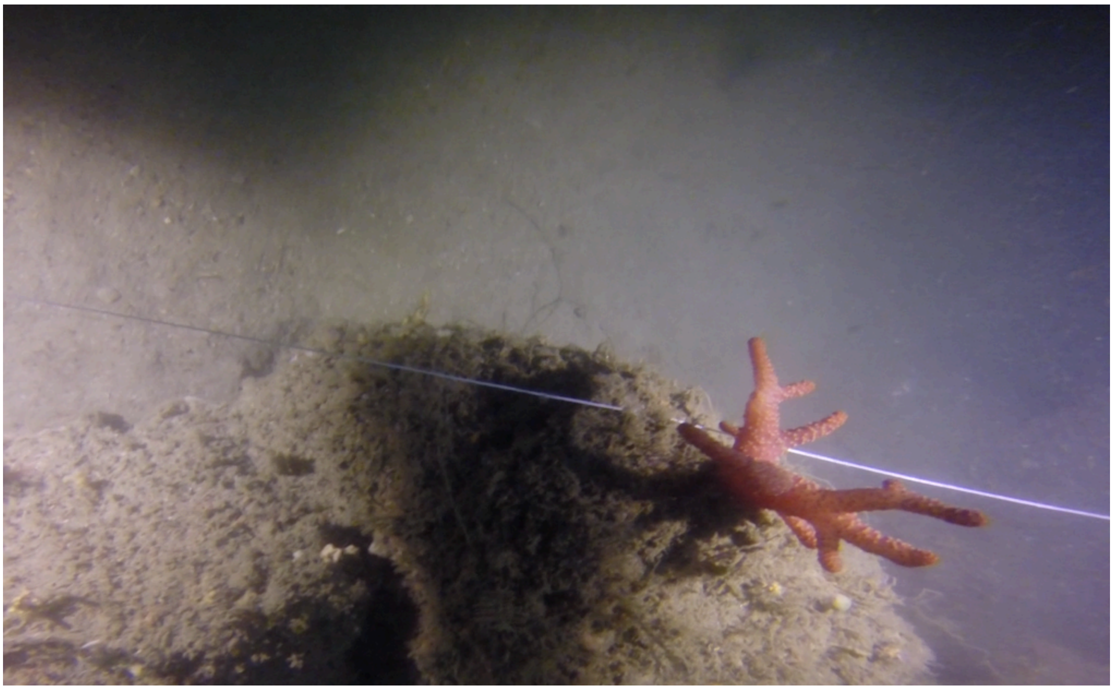

Alcyonium palmatum

Usually pale pink resembling a hand (palm) shape; apical region of branches thiner than base.

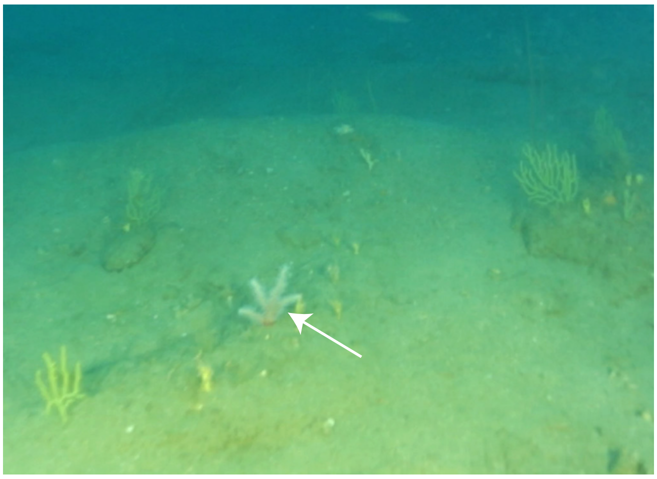

Alcyonium sp.

Pale (almost white) *Alcyonium* colony.

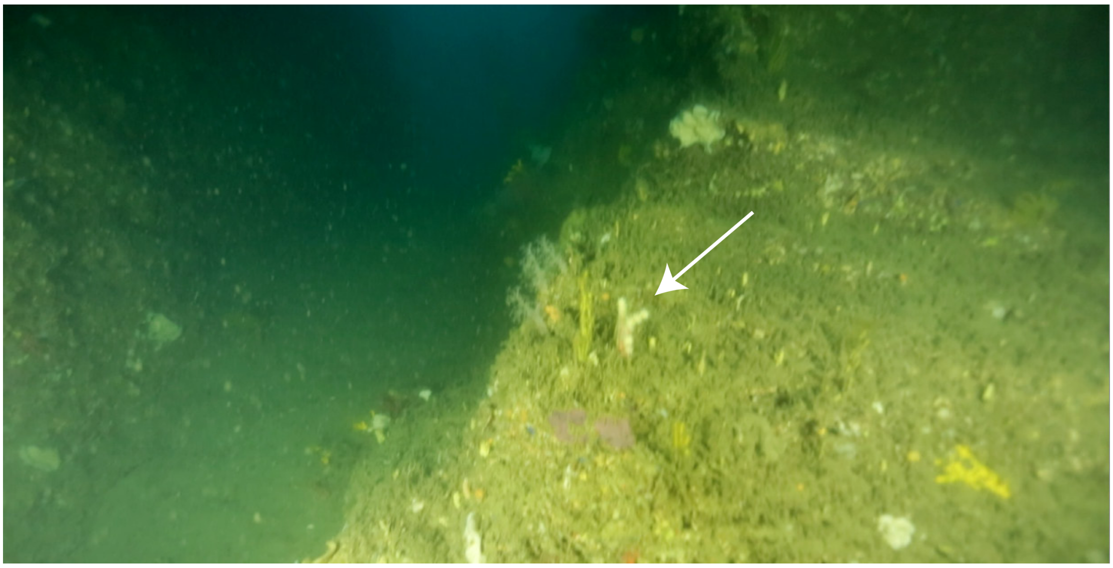

Paramuricea clavata

Most *P. clavata* in southern Portugal are entirely yellow. The red colortype is rare in the south coast but predominant at some locations in the west coast. They form very dense aggregations throughout the circalittoral and sciaphillous environments such as submerged caves. This is a flexible gorgonian and bends with moderate water current.

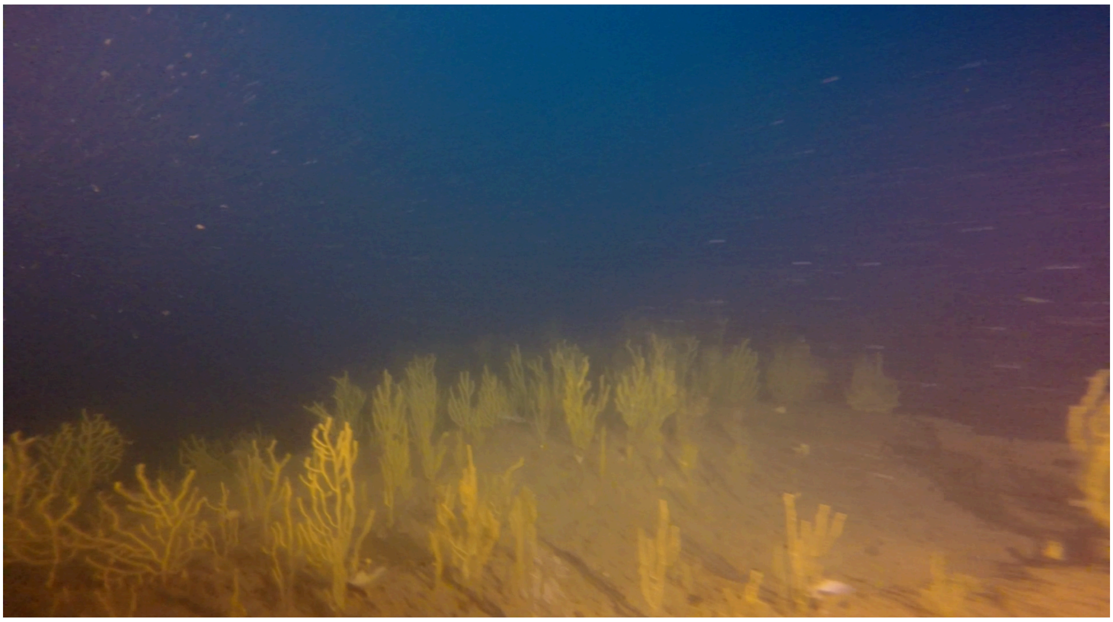

Corallium rubrum

Left arrow points to a colony with open polyps and right arrow points to a colony showing retracted polyps.

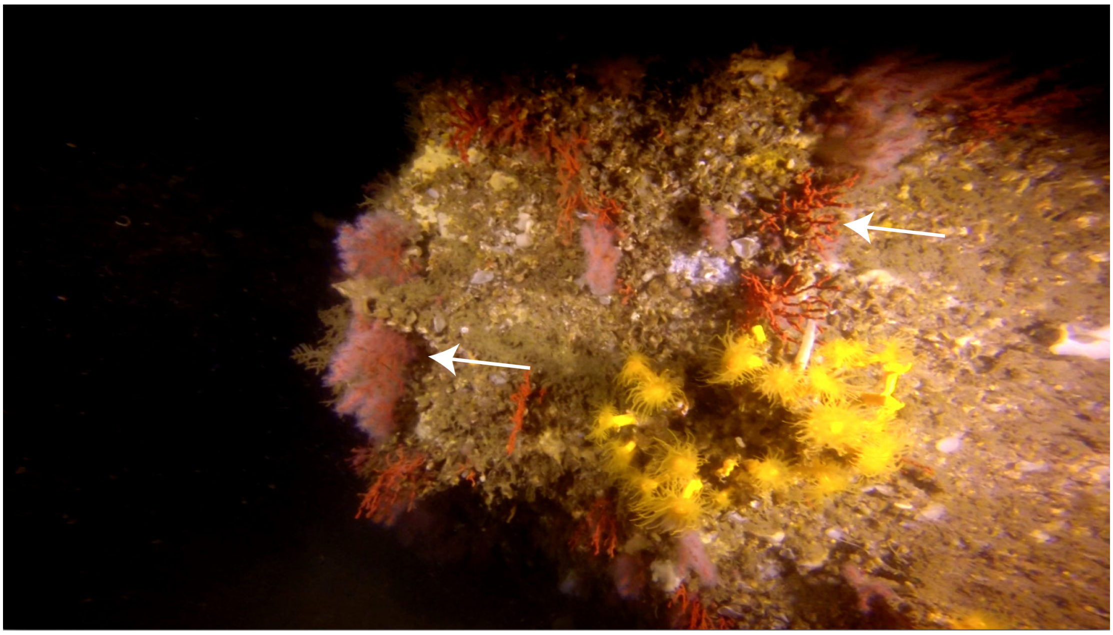

Eunicella verrucosa / *Eunicella* sp.

E. verrucosa (Ev) is white with a visible stalk and prominent calices. Whenever stalk, calices or color was not clear it was assigned to *Eunicella* sp (Esp).

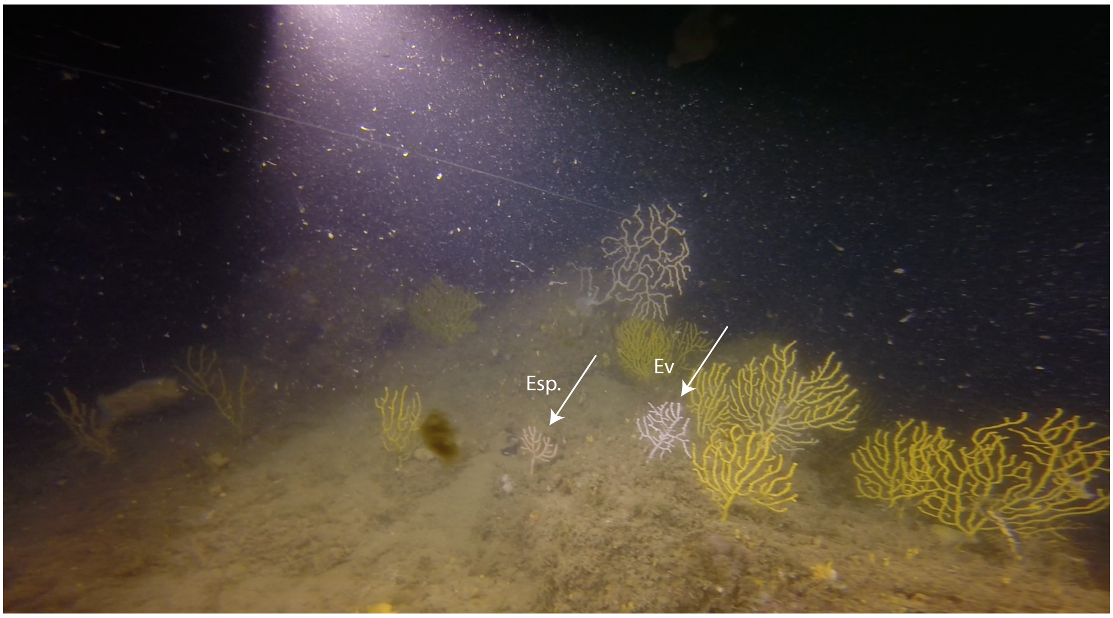

Eunicella filiformis

Left arrow points to an unbranched colony whereas right arrow points to a branched colony. Colonies of *E. filiformis* have been previously identified with samples collected in this region.

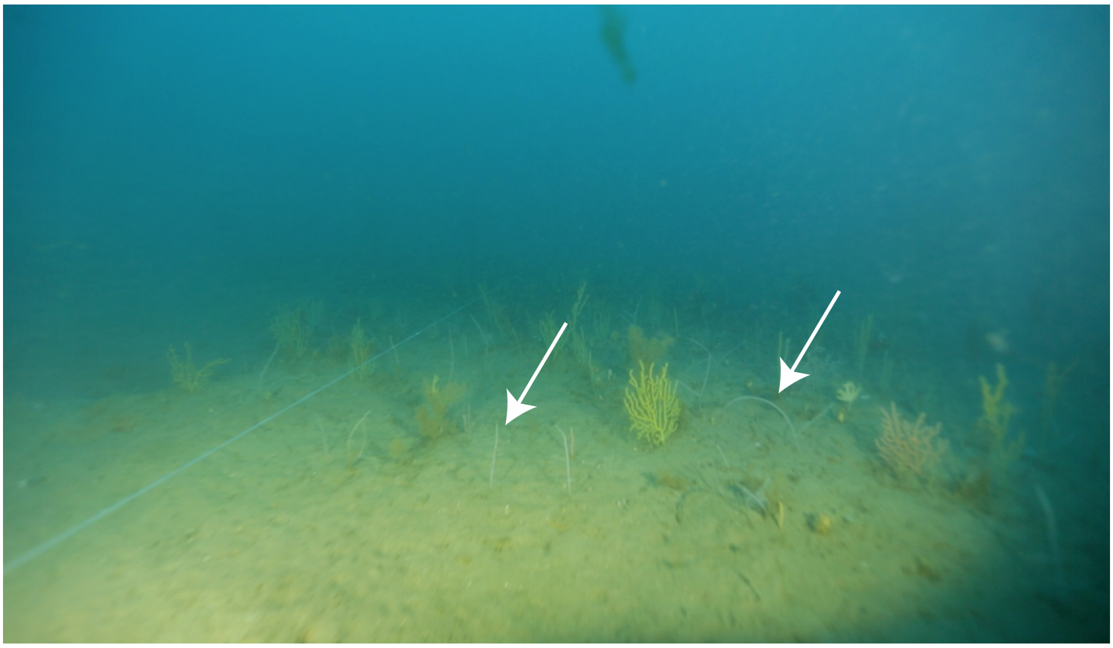

Ellisella paraplexauroides

Giant orange gorgonian; polyps are white but very small and usually only visible if seen from a short distance if not retracted. Distinctive branching pattern in a candelabrum shape, that is, with branches growing upwards in several planes, with little ramification. Very unflexible, proteinaceous skeleton is very hard; does not bend with oceanic currents. Usually between 1 m and 2.5 m tall in the region. The tallest (oldest) colonies present more branches and usually present epibionts. Occurs in low density over flat hard substrates with layers of fine sediment. Sciaphillous. Arrows in the image below point to several *E. paraplexauroides* in the point of highest density so far observed.

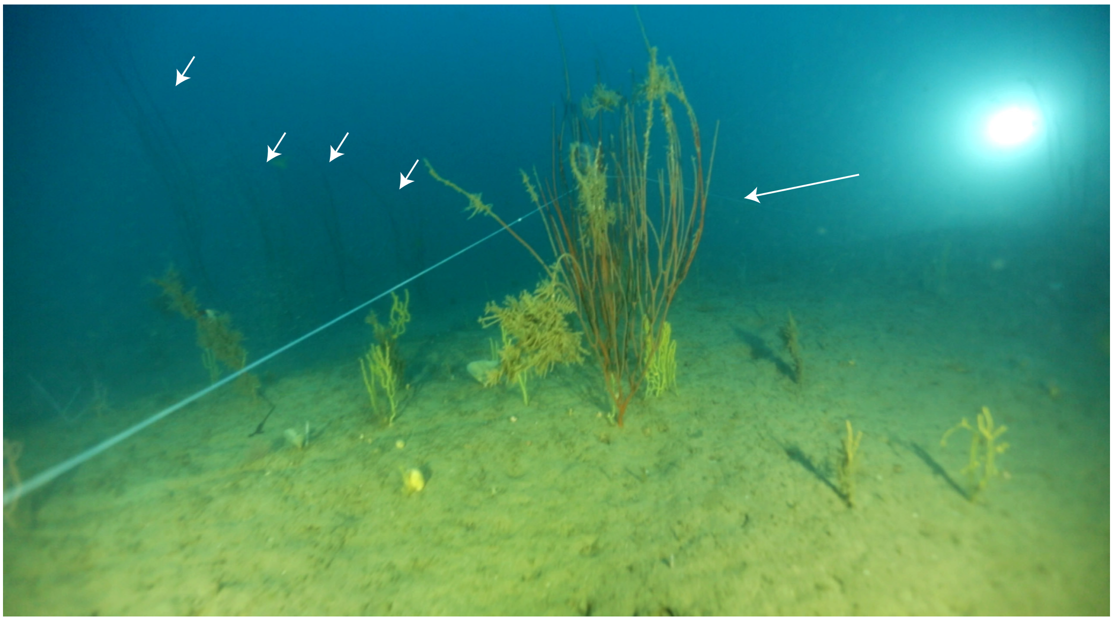

cf. Alcyoniina

These colonies occur in patches and form dense aggregations over hard substrates. Could be a Nidaliidae or *Paralcyonium.*

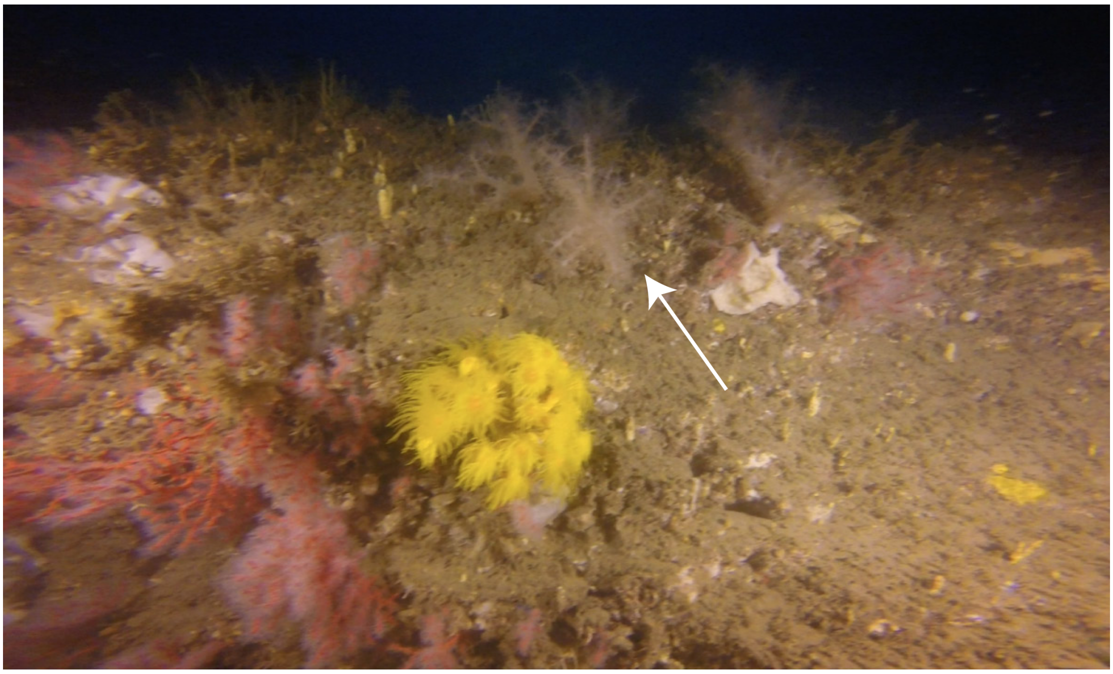

Octocorallia

Whip white coral: White, erect, unbranched octocoral, thicker than *E. filiformis*. Under moderate water current all *E. filiformis* bend with the flow but colonies of this unidentified octocoral don't.

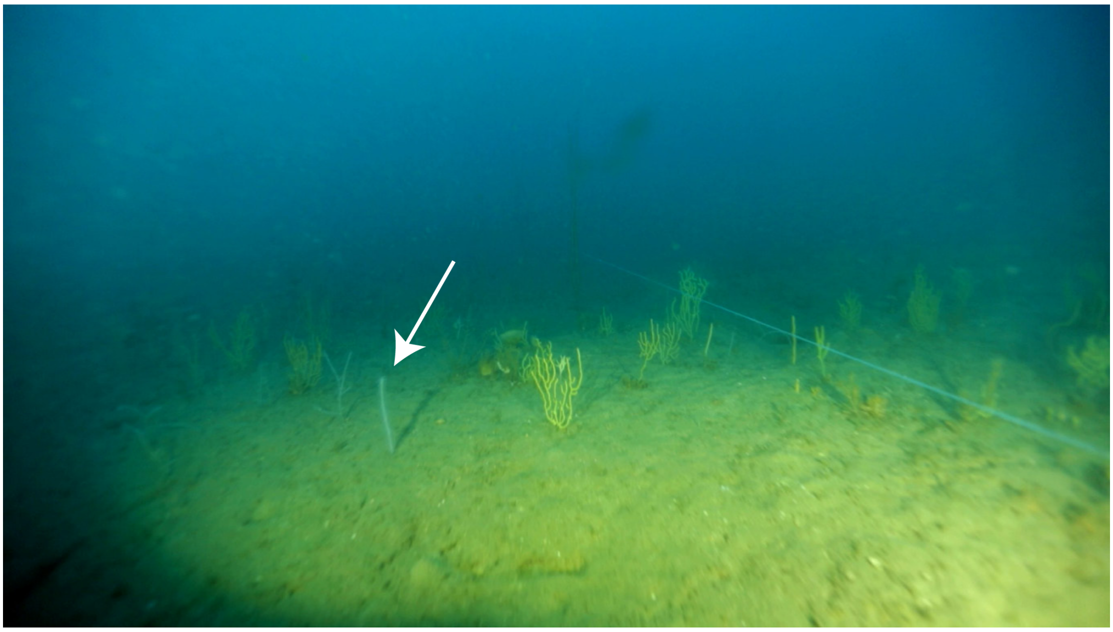

Anthozoa - Octocorallia, Pennatulacea

Veretillum cynomorium

Typical from soft substrates; usually solitary; usually presents an orange coenenchyme. Whitish-transparent polyps are visible when not retracted. Image below is from the ROV survey off Portimão, Portugal.

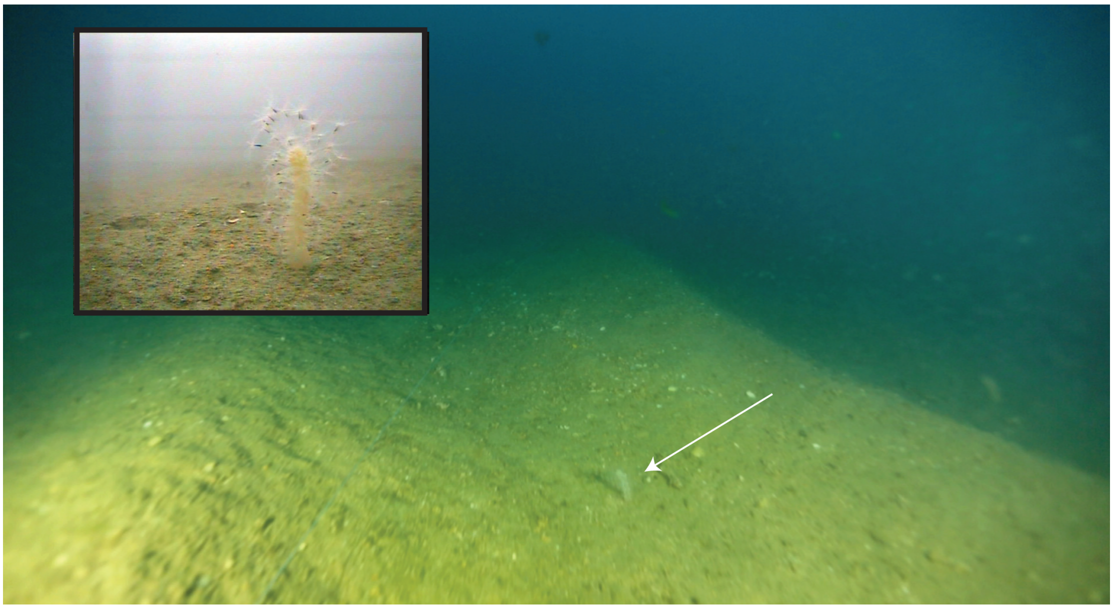

Anthozoa - Hexacorallia, Scleractinia

Dendrophyllia cornigera

Yellow colonial scleractinian coral.

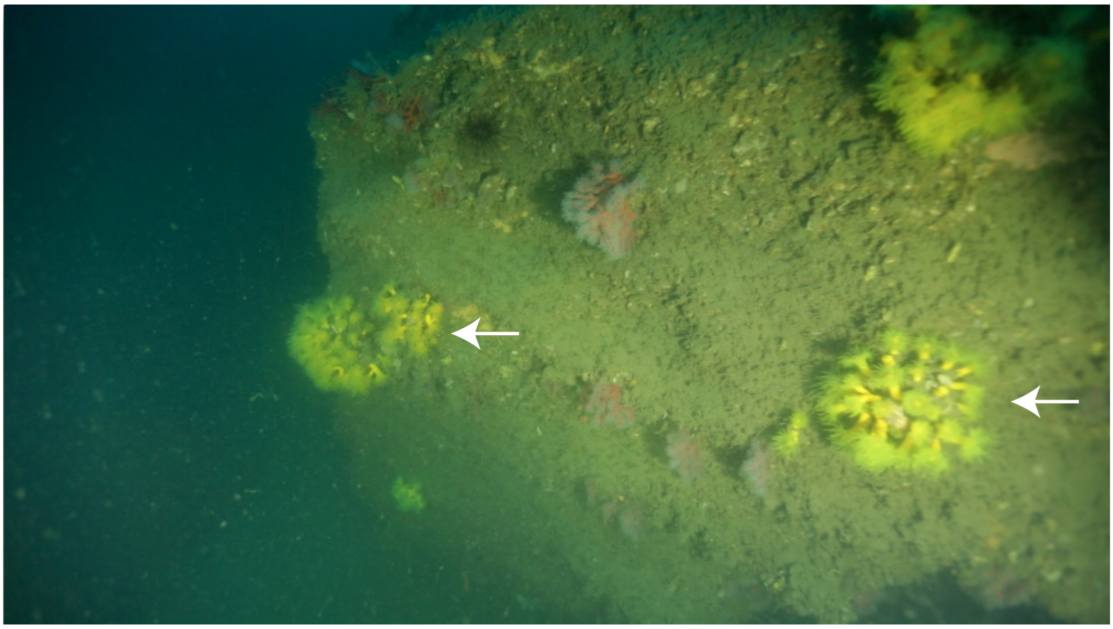

Dendrophyllia ramea

Orange colonial scleractinian coral with white polyps.

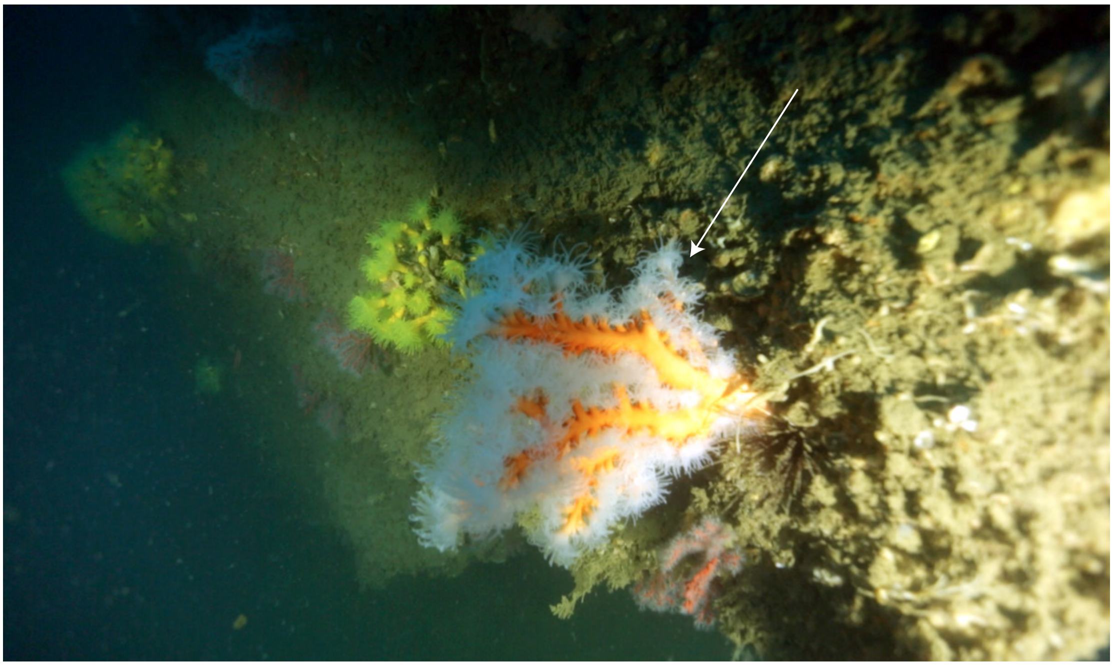

Anthozoa - Hexacorallia, Zoantharia

Parazoanthus axinellae

P. axinellae may form dense aggregation covering the top of circalittoral rocky reefs.

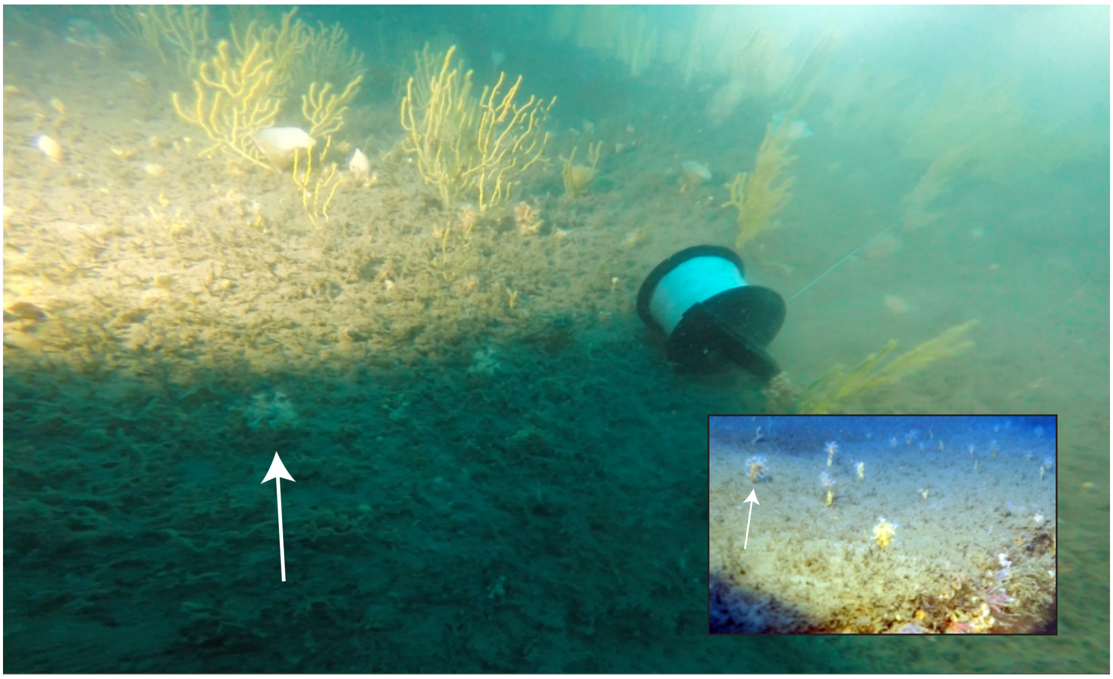

Savalia savaglia

Colonies may be white, light pink or yellow (see image below). They have been observed growing only over *P. clavata*. The white line visible in the image is the transect line.

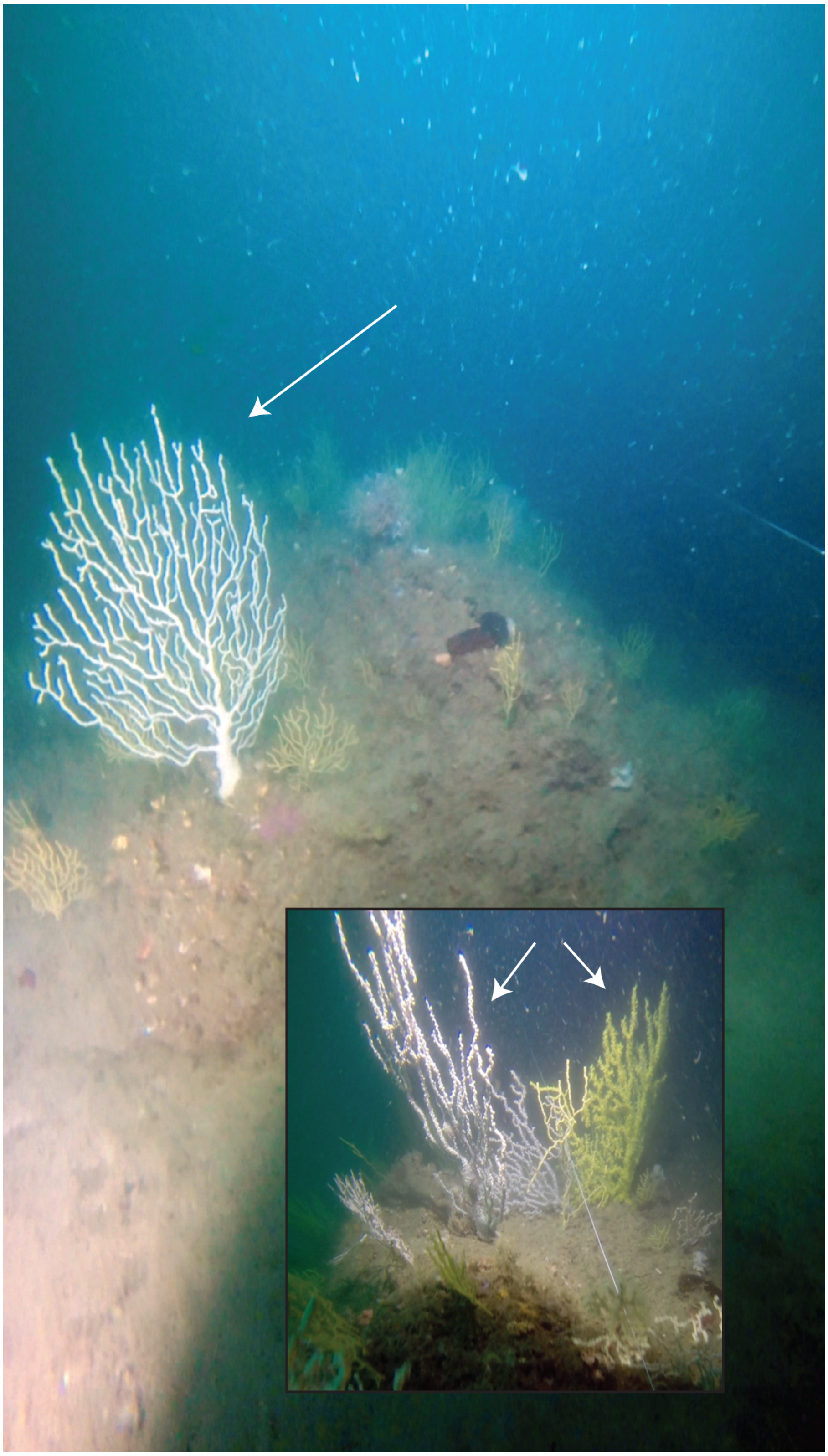

Bryozoa

Gymnolaemata

Myriapora truncata

Smaller image shows dense aggregations on rock overhangs. Vernacular name is false red coral due to a strong red coloration that fades after death of the colony. The colonies from this deep reef recorded exhibit a much lighter color. Presents dichotomic branching pattern and fragile skeleton. May form very dense aggregations over vertical walls and overhangs, as is shown in the lower right image.

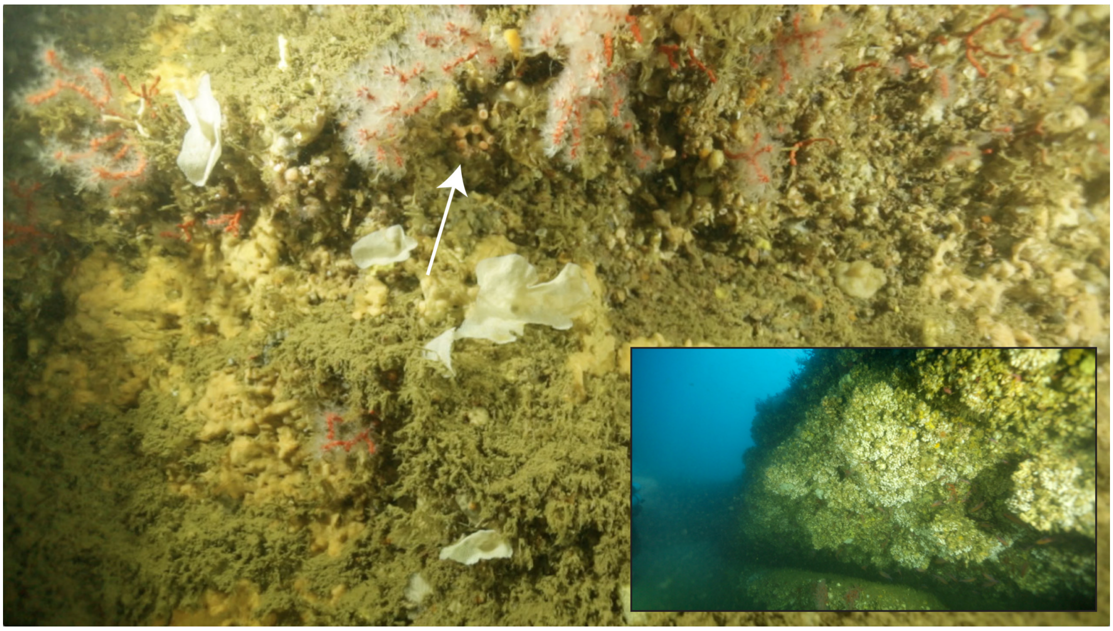

Reteporella cf. *grimaldii*

Cup-shape, white *Reteporella*. Very abundant at the deeper sites (90-100 m). Sample kindly identified by Javier Souto from the University of Vienna.

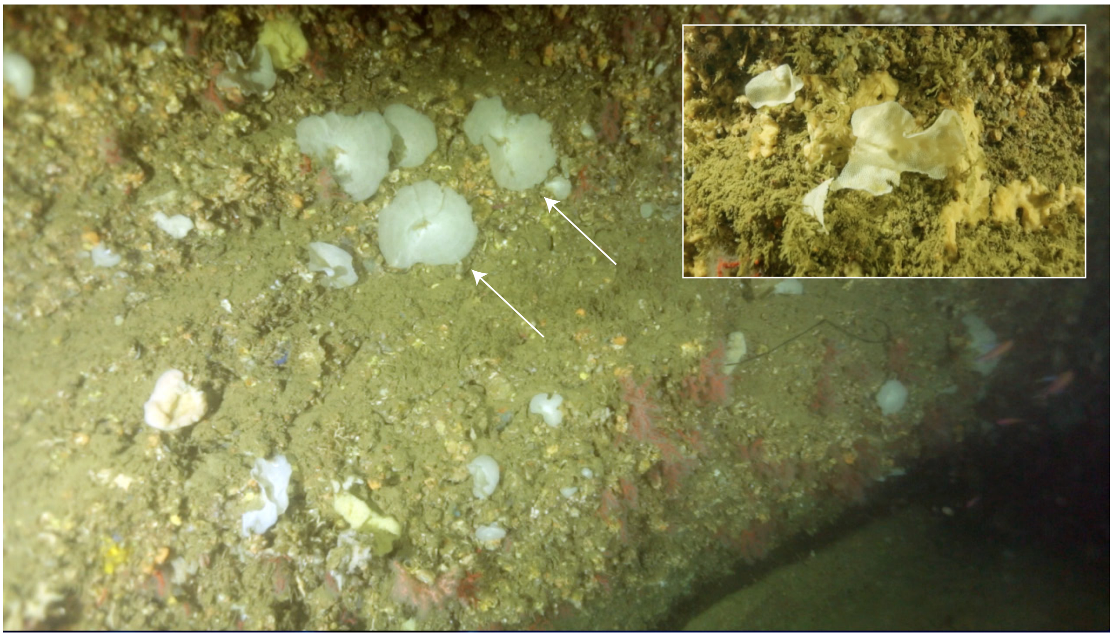

Annelida

Polychaeta

Sabella sp. / *Sabella spallanzani*

The image below is *Sabella* sp. *S. spallanzani* presents a two-layered crow of feeding tentacles, usually darker with yellowish bands but color varies.

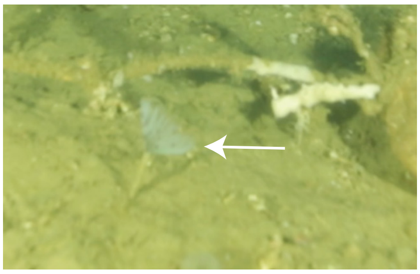

Serpulidae

Image below shows the same individual, left with red tentacles out and right with tentacles rectracted inside the tube.

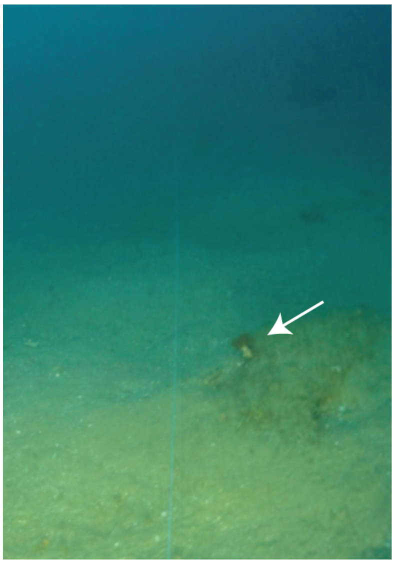
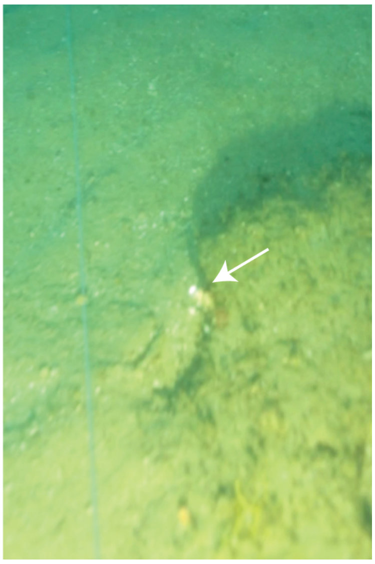

Arthropoda (Crustacea)

Malacostraca

Pallinurus elephas

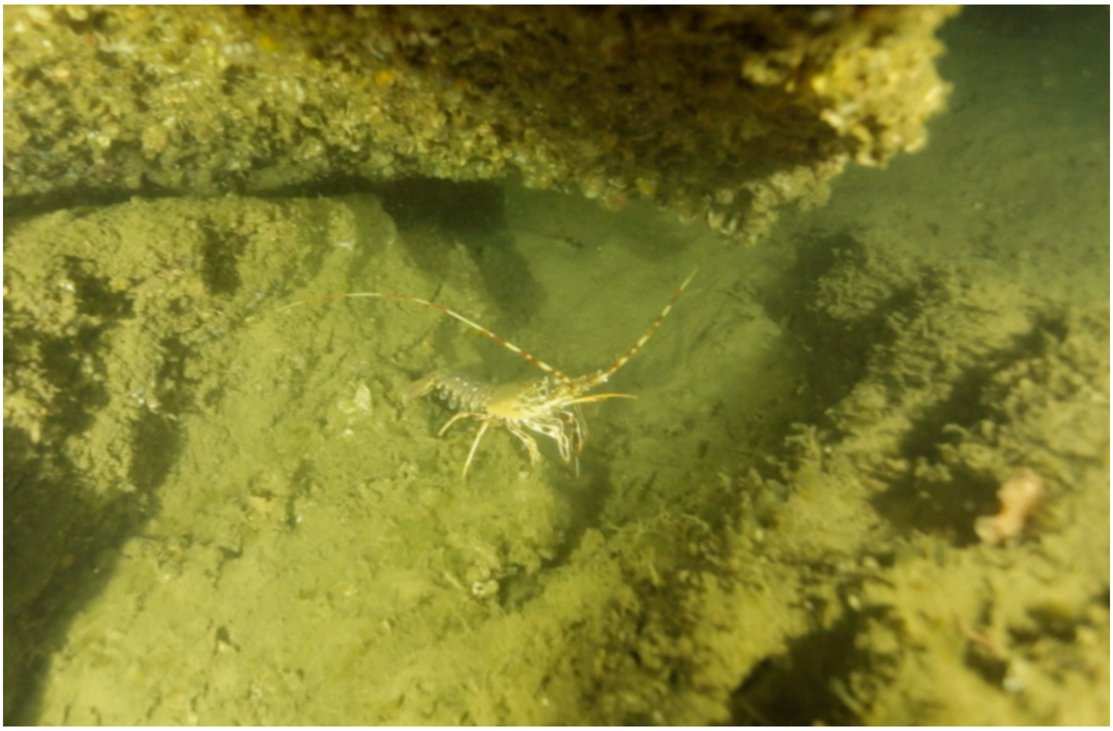

Mollusca

Bivalvia

Neopycnodonte cochlear

In the image bellow the red coral colonies are growing over the deep oysters (*N. cochlear*) that cover the rocky reef. Samples were collected and identifyed by expert (Carlos Afonso, University of Algarve). A lost fishing line is seen in the image entangled over the rocks and corals; it presents several epibionts, mainly hidroids *H. halecinum*.

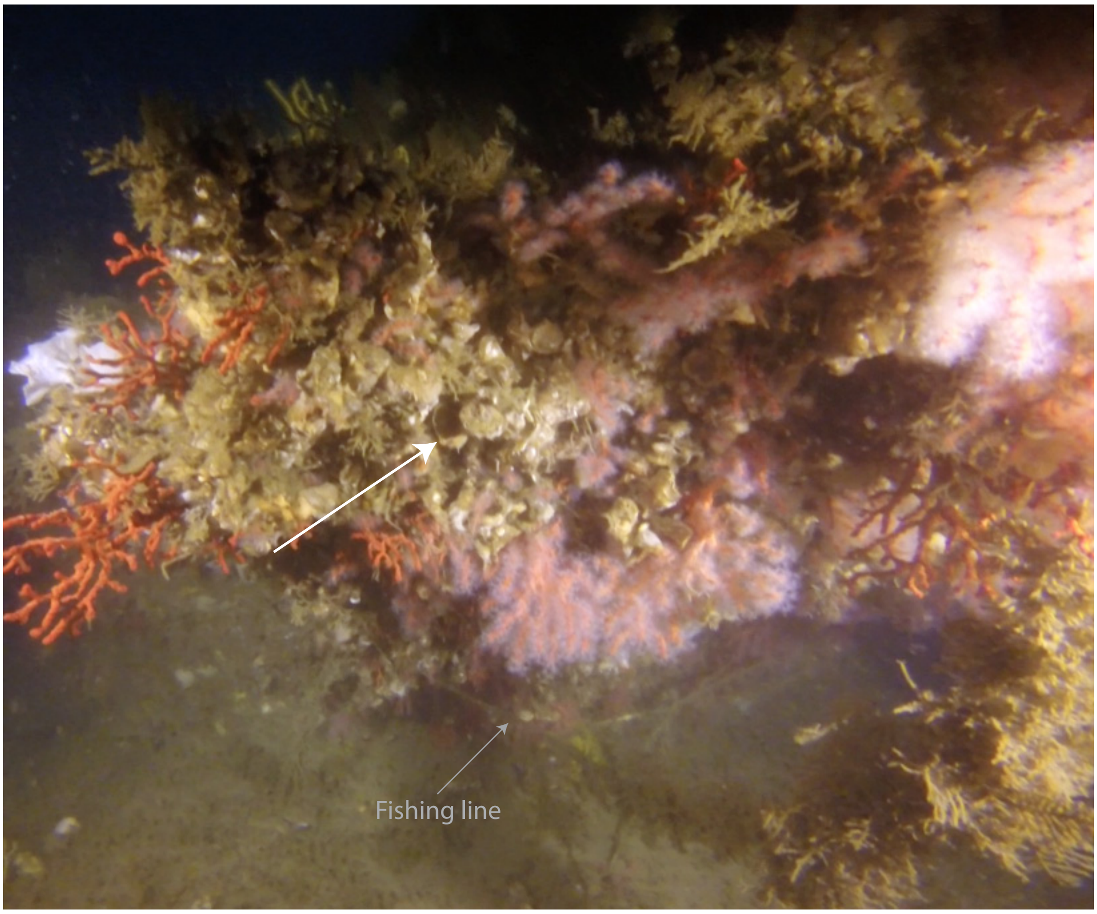

Brachyopoda

Brachyopoda
Unidentified brachiopod. Samples were collected for future identification.

Echinodermata

Asteroidea

Echinaster sepositus

Distinctive red sea star; may be found over hard ground or over the soft substrate that occurs between rocky reefs. The image below presents distortion caused by motion and wide angle camera.

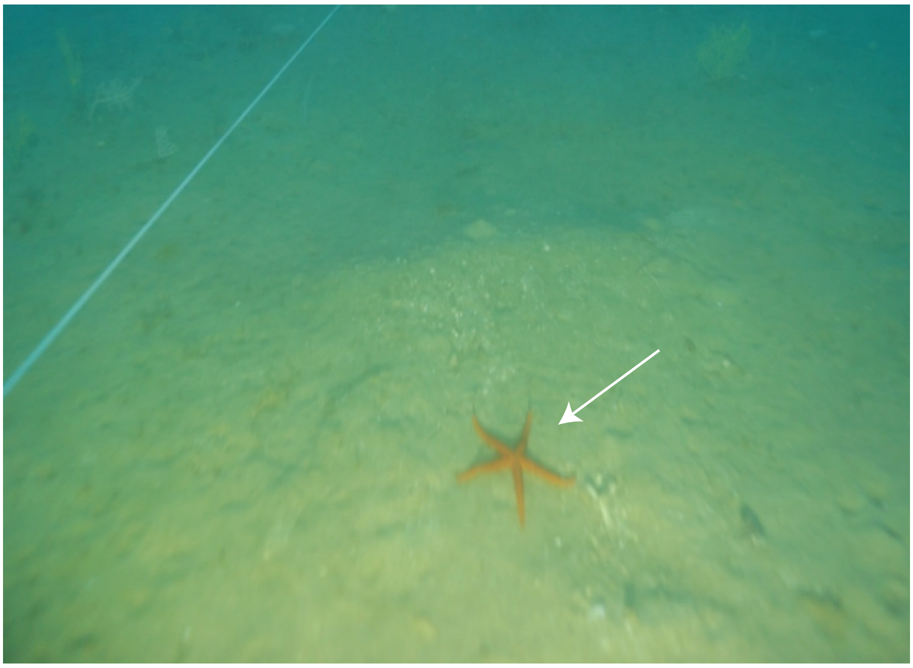

Marthasterias glacialis

May present different colors as seen below, but papillae are usuallu very raised and white or pale. A lost fishing line entangled over the reef is visible in the first image.

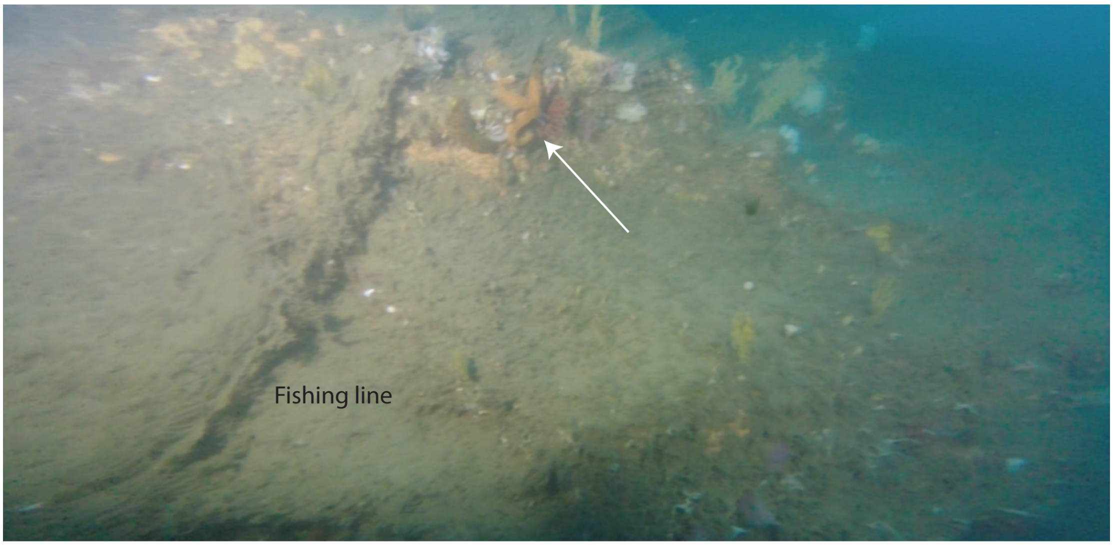

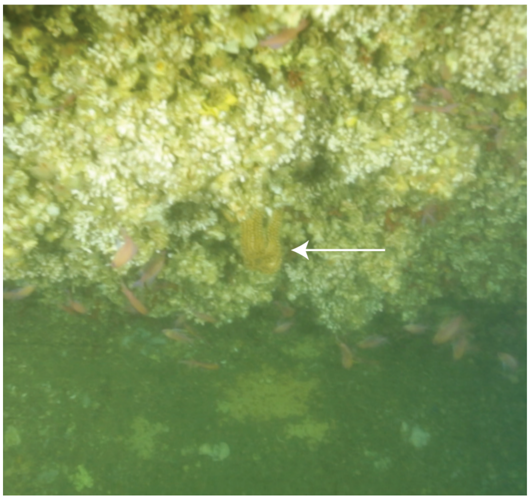

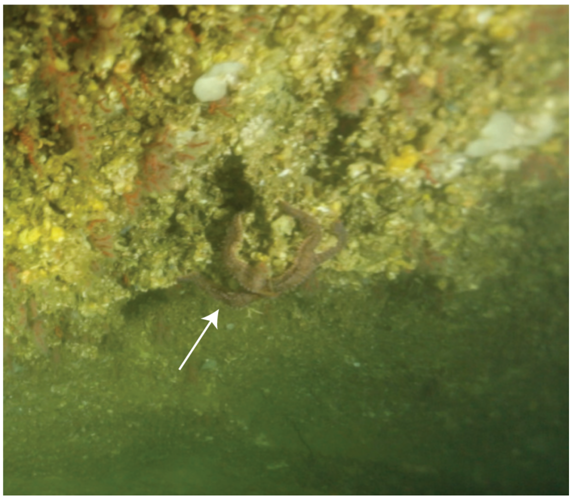

Ophiuroidea

Astrospartus mediterraneus

Conspicuous circalittoral basket starfish. Small picture shows one over a red coral colony. Arms are extended in the typical feeding position.

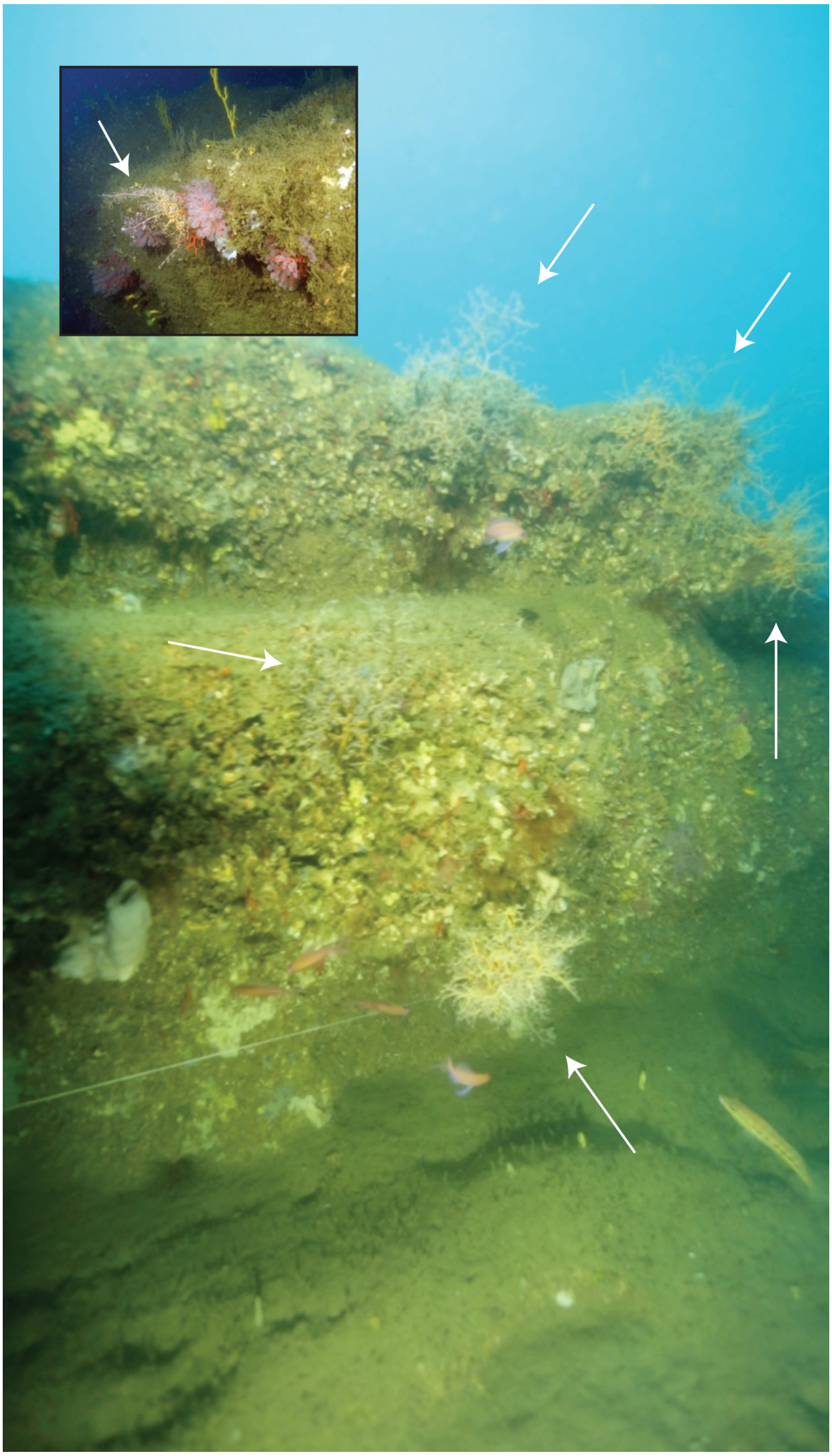

Echinoida

Centrostephanus longispinus

Black sea urchin with distinctive long spines. Usually found inside holes or depressions over the hard substrate; spines are visible outside of the holes. Younger individuals may present a more or less visible (in vdeo imagery) anular pattern along the spines.

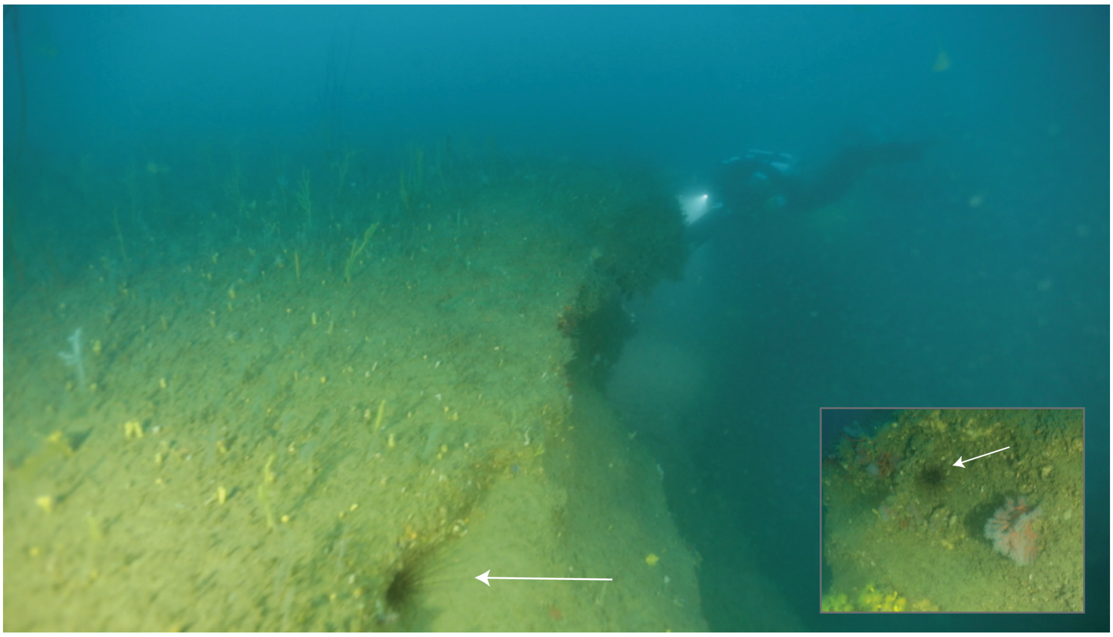

Holothuroidea

Holothuria forskali / *Holothuria* sp.

Dark or dark and yellow sea cucumber with flattened ventral area of a pale brown color, sometimes yellow; body dorsal surface of a rough appearance owing to the raised conical papillae, usually presenting a white or pale dot. When these features (distinctive yellow spots, ventral yellowish side and papillae) were not clearly visible, the organism was assigned to the genus (*Holothuria* sp.). In the lower right side of the image a large net and lines are visible entangled over the rocky reef.

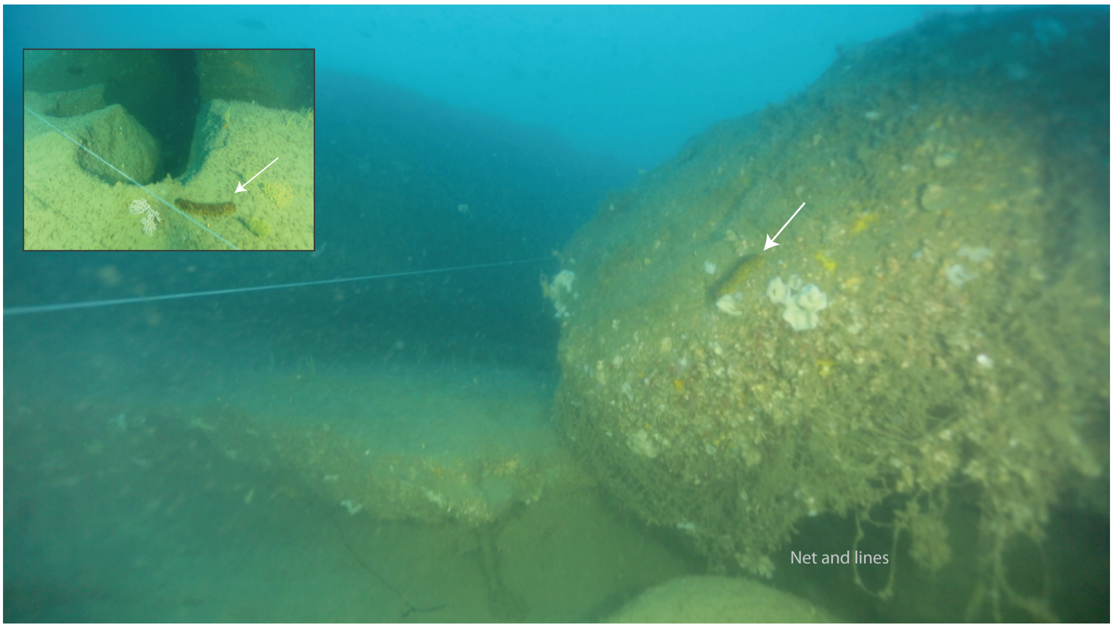

Pawsonia saxicola

Only the tree-like feeding tentacles are visible, the rest of the body (white) is inside a hole. The 10 feeding tentacles are darkish purple.

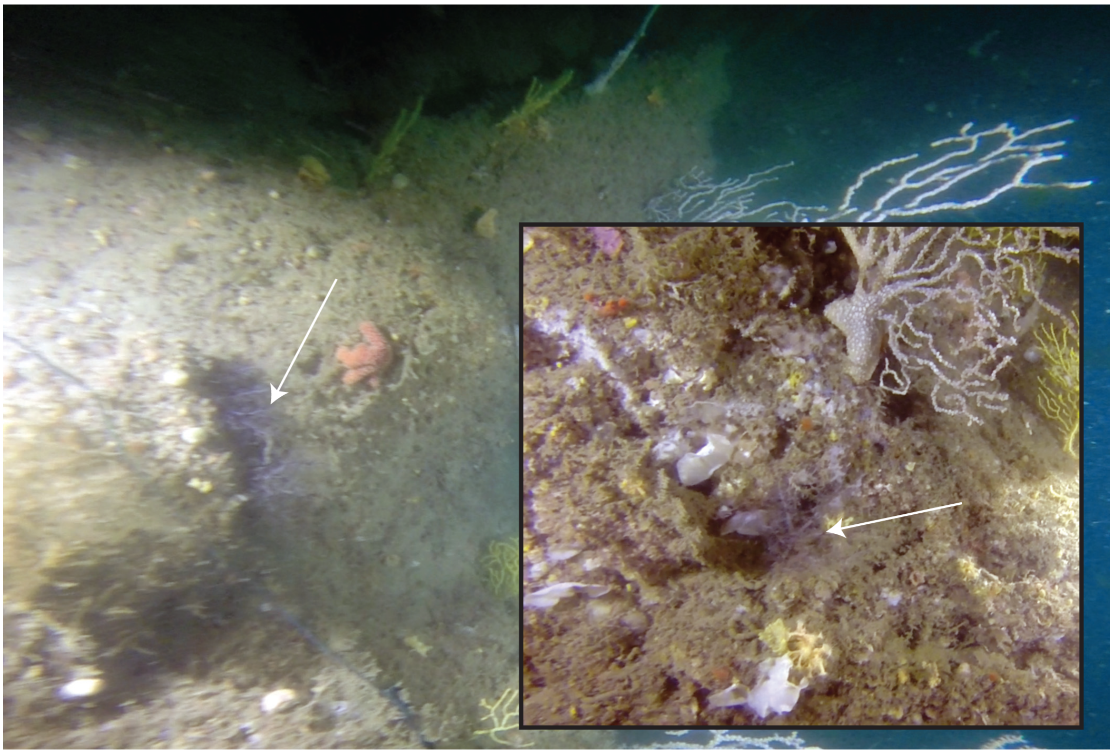

Echiura

Echiuroidea

Bonellia viridis

Common in the region, from shallow waters to the deeper circalittoral; sciaphillous. Black worm, very long (up to about 2 m in length), that ends in a conspicuous T shape. Is always observed between rocks and soft sediment; lives inside the hole and quickly retracts into it if disturbed. Image is distorted due to wide-angle and motion of camera.

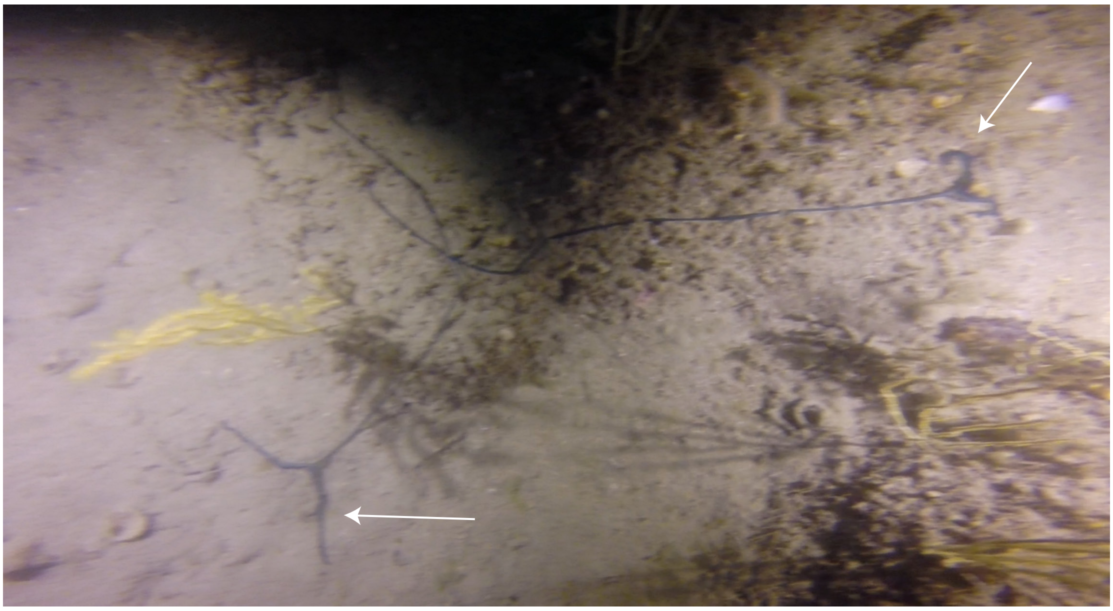

Chordata

Ascideacea

Ascidiacea

Colonial, transparent tunicate. Could be *Clavelina lepadiformis* or *Diazona violacea*.

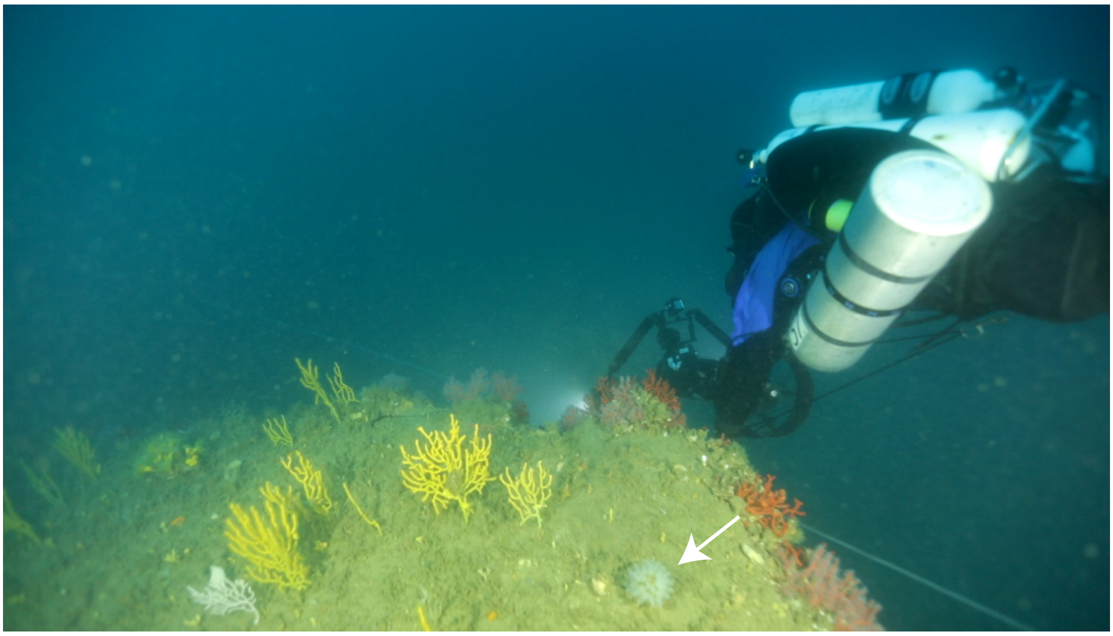

Halocynthia papillosa

Presents a distinctive T-shape caused by perpendicular position of atrial syphon.

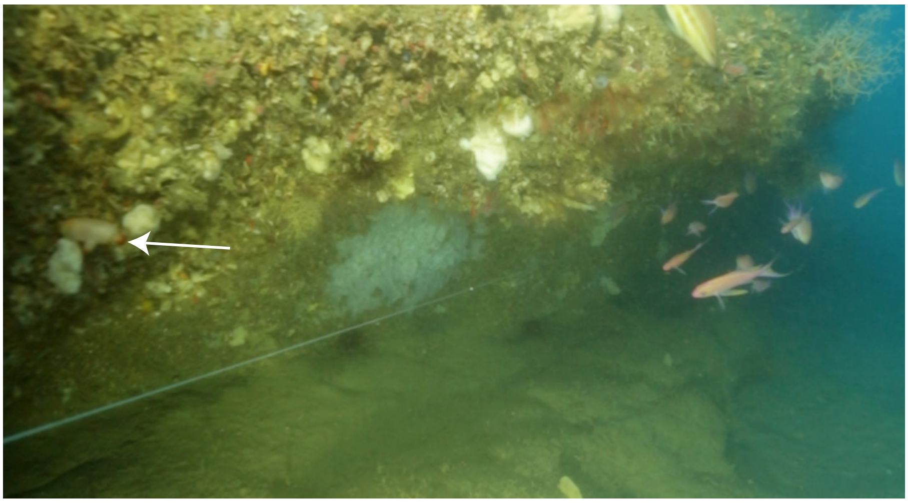

Actinopteri (Fish)

Acantholabrus palloni

Solitary reef fish. Has a dark spot on upper part of caudal peduncule and white spots on the upper flank.

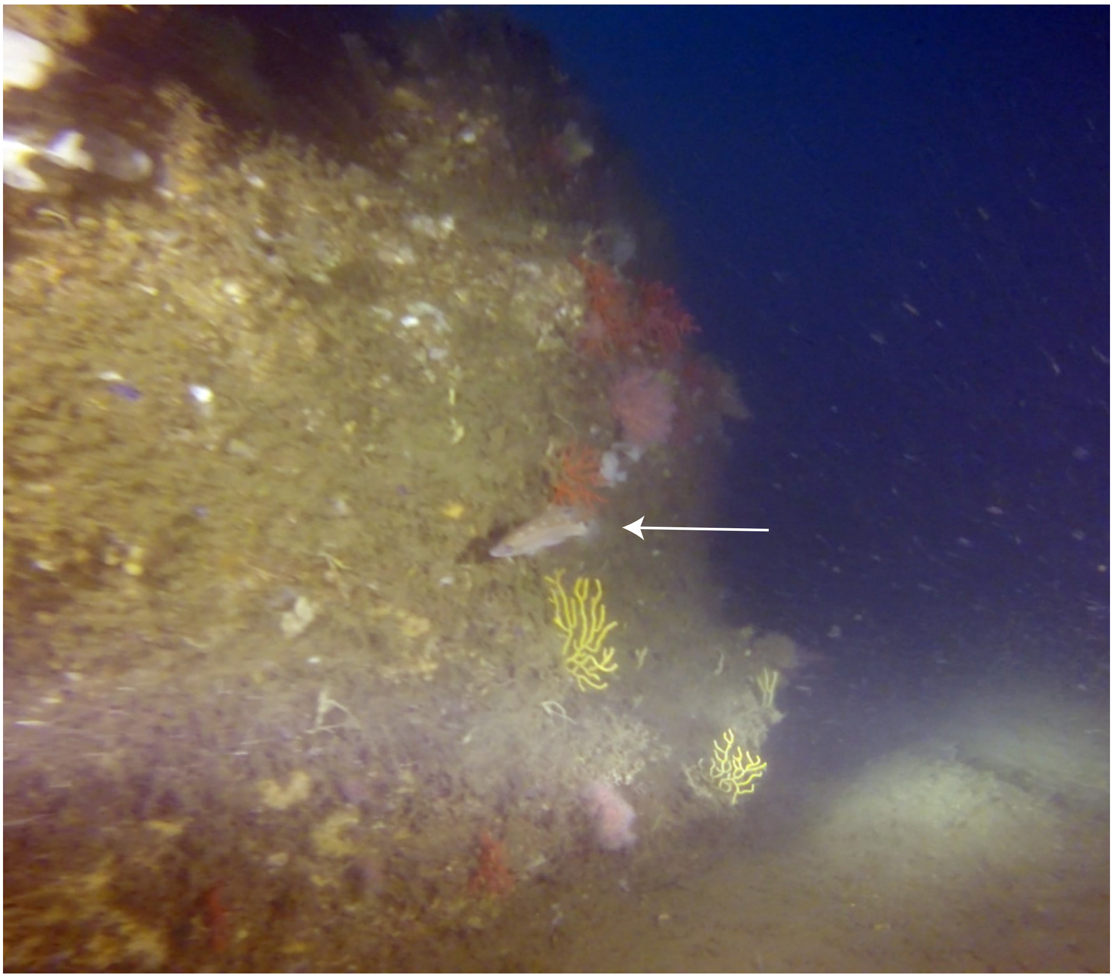

Anthias anthias

Gregarious pink fish with long fins; yellow and blue colors are visible in males (present longer pelvic and caudal fins); form large schools.

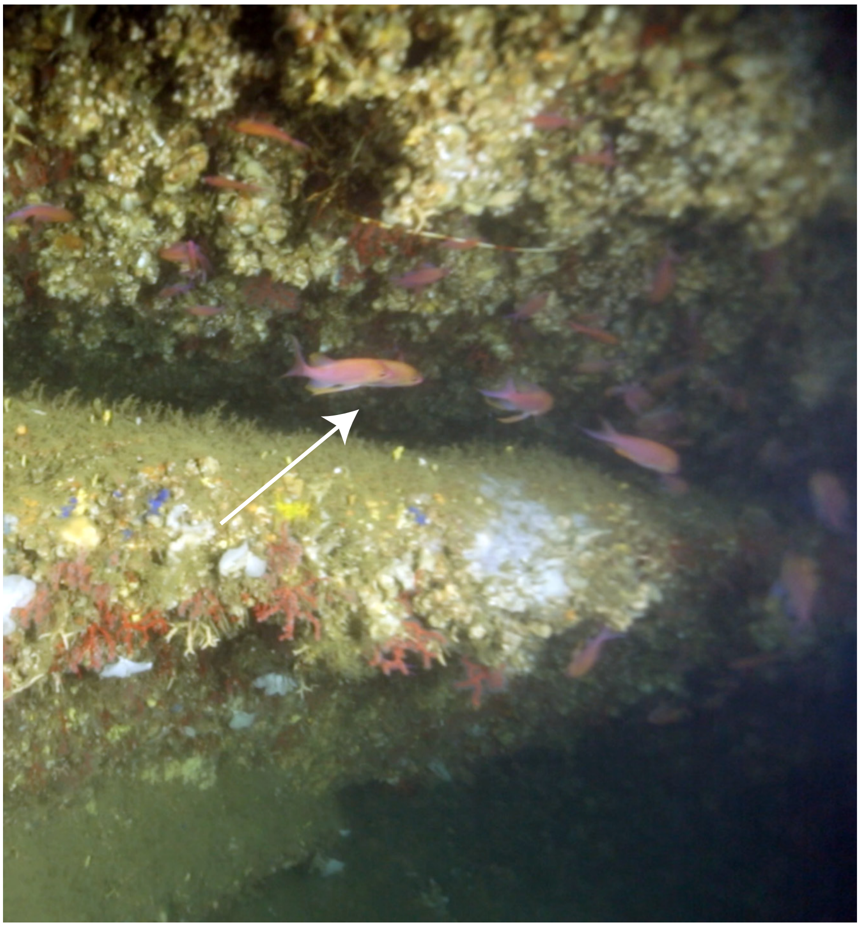

Scorpaena sp.

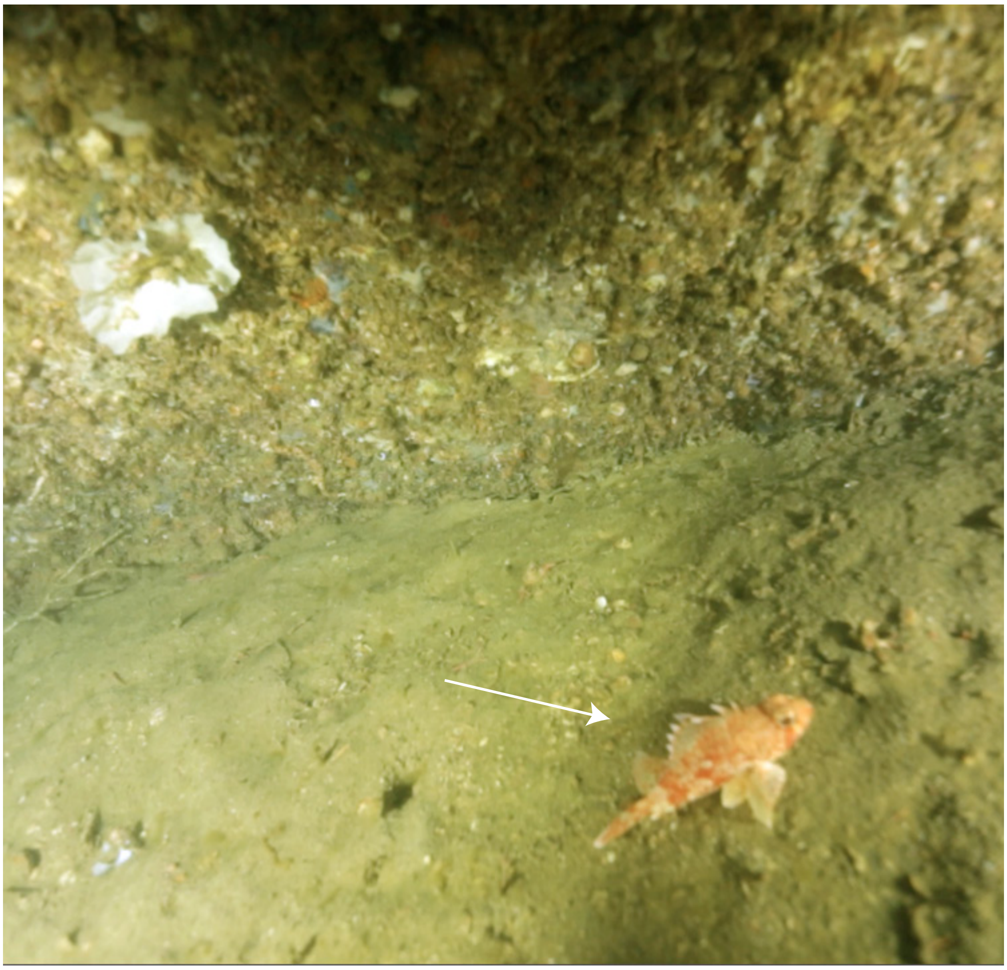

Serranus cabrilla

Comber; presents a distinctive stripped pattern in the flanks with one to three clearly visible white longitudinal bands and several vertical lines alternating between darker and lighter brown.

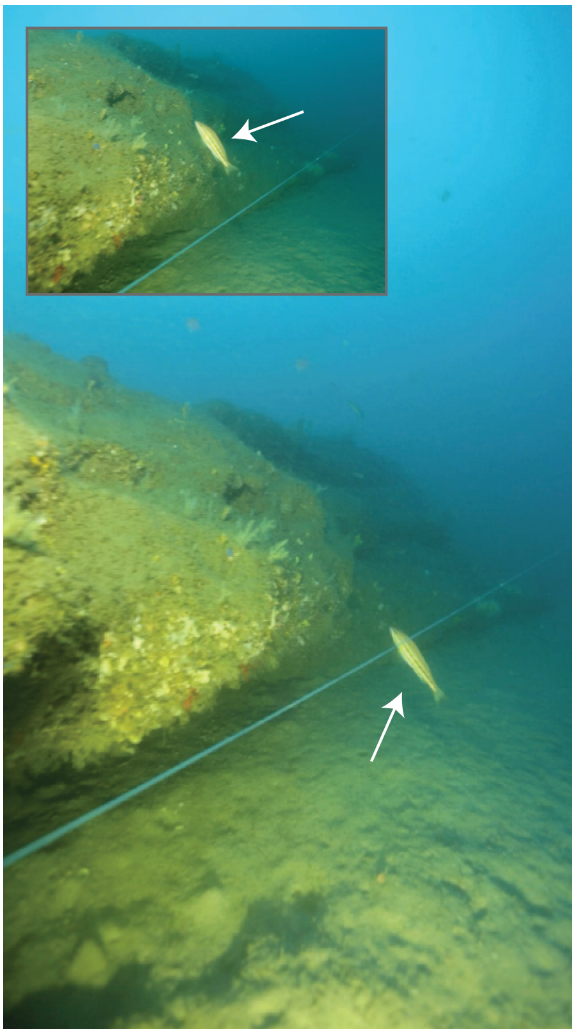

Trisopterus luscus

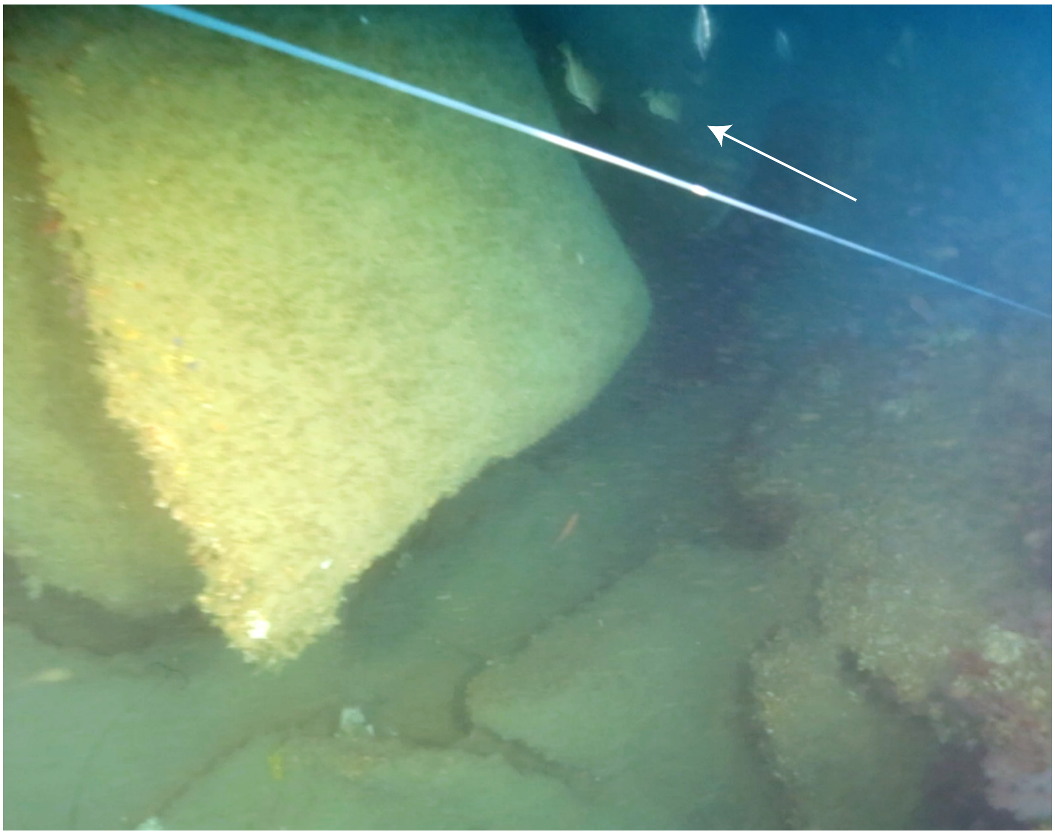

Diplodus vulgaris

Easily identified by the two vertical dark bands, one closer to the head and the other near the caudal peduncule.

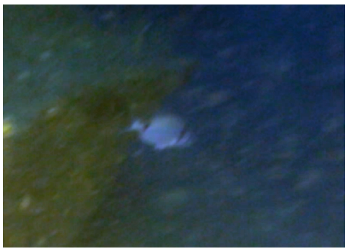

Actinopteri

Unidentified, solitary fish. Some are likely *Lapanella fasciata*, which is a wrasses known to occur at these depths in the region (e.g. the fish indicated in the image by the top arrow).

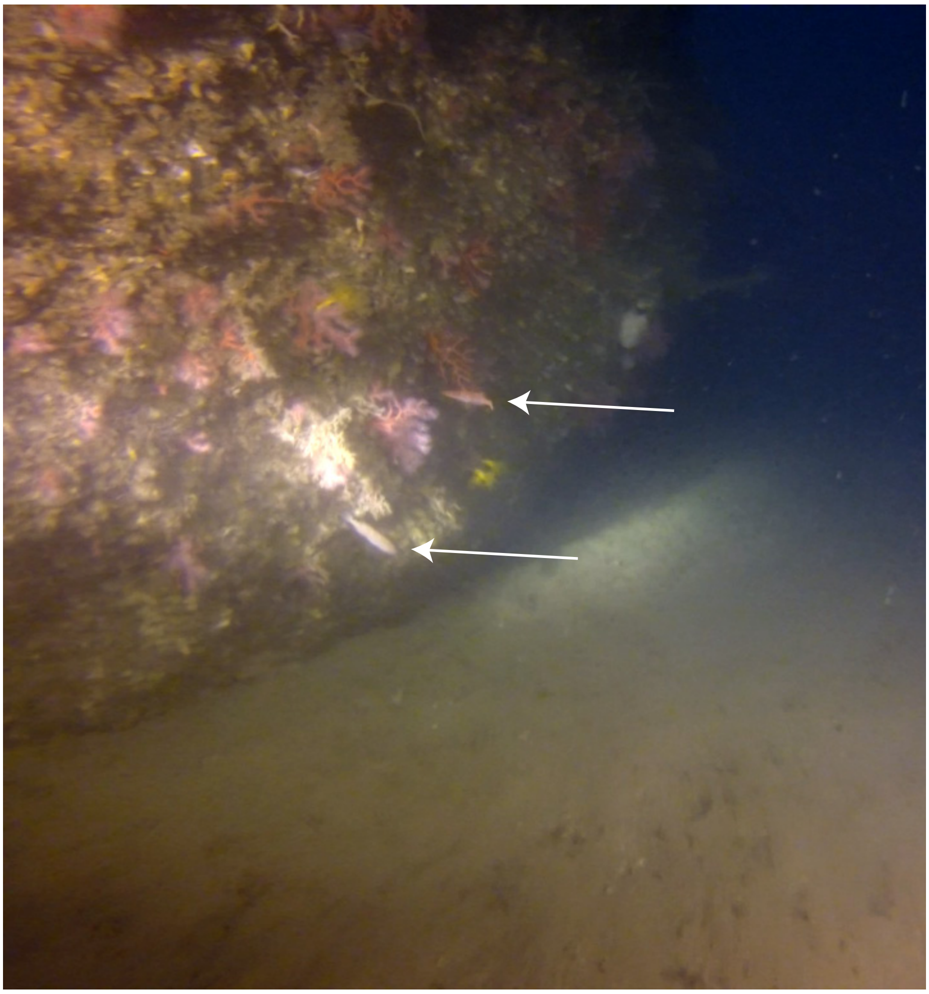

Clupeidae

Forms dense schools from the bottom (swarming the reefs) to the surface.

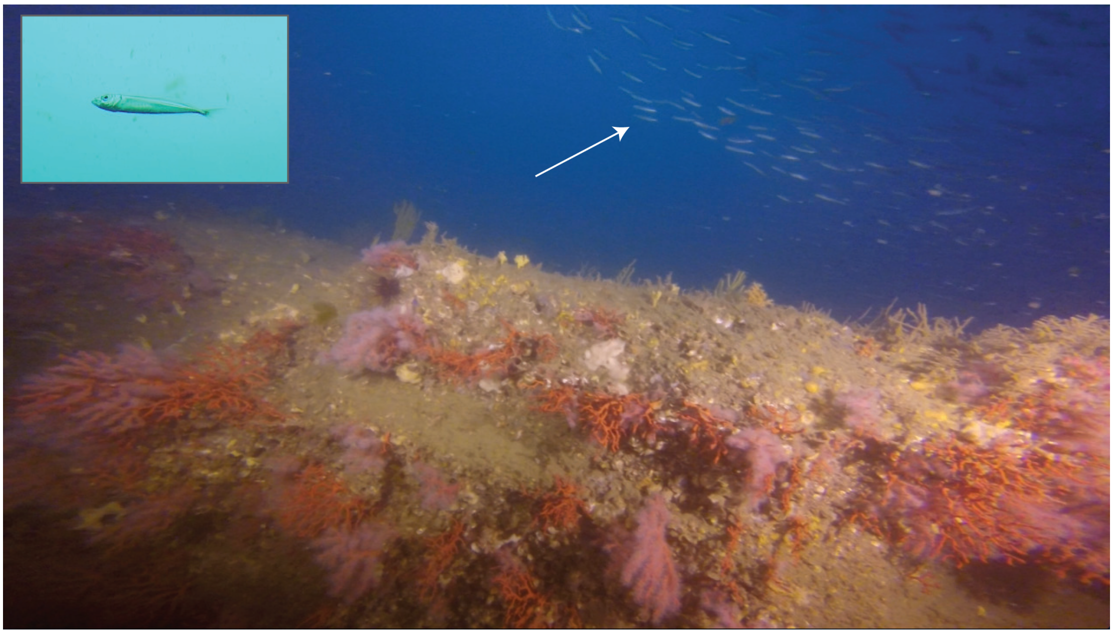

Supplement: S1 File — Names correspond to names in the manuscript. Whenever possible a description of major characteristics was included. Images edges present distortion of the field of view caused by the wide-angle camera and motion. Arrows in the images indicate the taxa of interest. Images were extracted from the underwater video frames obtained during field surveys in the Global Underwater Expedition 2014 (http://projectbaseline.org; http://globalsubdive.com/expeditions) in collaboration with project Deep Reefs (http://www.deepreefs.com). Document has 44 pages (>100MB). Can be found at http://www.figshare.com under Doi: 10.6084/m9.figshare.1544524. URL: https://figshare.com/s/8350279a5bb411e5b61406ec4b8d1f61. (PDF) [file pone.0147228.s005.pdf]
